# Computationally Assisted Lead Optimization of Novel Potent and Selective MAO-B Inhibitors

**DOI:** 10.3390/biomedicines9101304

**Published:** 2021-09-24

**Authors:** Vedanjali Gogineni, Manal A. Nael, Narayan D. Chaurasiya, Khaled M. Elokely, Christopher R. McCurdy, John M. Rimoldi, Stephen J. Cutler, Babu L. Tekwani, Francisco León

**Affiliations:** 1 Department of BioMolecular Sciences, Division of Medicinal Chemistry, University of Mississippi, Oxford, MS 38677, USA; gogineni@musc.edu (V.G.); cmccurdy@cop.ufl.edu (C.R.M.); jrimoldi@olemiss.edu (J.M.R.); sjcutler@cop.sc.edu (S.J.C.); 2 Department of Drug Discovery, Biomedical Sciences and Public Health, College of Pharmacy, Medical University of South Carolina, Charleston, SC 29425, USA; 3 Department of Chemistry, Institute for Computational Molecular Science, Temple University, Philadelphia, PA 19122, USA; manal.nael@temple.edu (M.A.N.); kelokely@temple.edu (K.M.E.); 4 Department of Pharmaceutical Chemistry, Faculty of Pharmacy, Tanta University, Tanta 31527, Egypt; 5 Division of Drug Discovery, Southern Research, Birmingham, AL 35205, USA; nchaurasiya@southernresearch.org; 6 National Center for Natural Products Research, Research Institute of Pharmaceutical Sciences, University of Mississippi, Oxford, MS 38677, USA; 7 Department of Medicinal Chemistry, College of Pharmacy, University of Florida, Gainesville, FL 32610, USA; 8 Department of Drug Discovery and Biomedical Sciences, College of Pharmacy, University of South Carolina, Columbia, SC 29208, USA

**Keywords:** flavonoids, Parkinson’s Disease, monoamine oxidases A and B, acacetin, docking

## Abstract

A series of dietary flavonoid acacetin 7-*O*-methyl ether derivatives were computationally designed aiming to improve the selectivity and potency profiles against monoamine oxidase (MAO) B. The designed compounds were evaluated for their potential to inhibit human MAO-A and -B. Compounds **1c**, **2c**, **3c**, and **4c** were the most potent with a Ki of 37 to 68 nM against MAO-B. Compounds **1c**–**4c** displayed more than a thousand-fold selectivity index towards MAO-B compared with MAO-A. Moreover, compounds **1c** and **2c** showed reversible inhibition of MAO-B. These results provide a basis for further studies on the potential application of these modified flavonoids for the treatment of Parkinson’s Disease and other neurological disorders.

## 1. Introduction

Parkinson’s Disease (PD) is one of the most prevalent neurodegenerative disorders [1], affecting over four million people worldwide. The causes of PD remain unknown. However, the disease is known to arise from interaction between environmental and genetic factors, resulting in progressive degeneration of neurons in the brain. Despite decades of research, the molecular pathways involved in neurodegeneration, the nature of the interaction, and the identity of the factors are poorly understood [2]. PD is characterized by the progressive loss of dopaminergic neurons. Most of the PD cases are sporadic, but rare familial forms have also been recognized. The common pathways underlying the pathogenesis of PD include oxidative stress, mitochondrial quality control, and protein degradation processes. Understanding the possible reasons behind these common processes provides various targets, which are therapeutically relevant to the discovery of disease-modifying treatments [1].

The etiology of PD remains obscure, but oxidative stress is thought to play a significant role in dopaminergic neurotoxicity. At the cellular level, PD is associated with excessive production of reactive oxygen species (ROS), resulting in metabolic alteration of catecholamine, the mitochondrial electron-transporter chain (METC), or enhancement of the deposition of iron in the substantia nigra pars compacta (SNpc) [3]. Recently, special focus has been directed to the role of oxidative stress in neurological abnormalities, including PD. Excessive production of ROS has been considered to be a significant cause of neuronal death [4]. The most effective pharmacological treatment to date is levodopa–dopamine substitution therapy. Recently, attention has been shifted towards the enzyme monoamine oxidase B (MAO-B) as a secondary drug target in PD [5].

MAO-B is one of the two subtypes of the monoamine oxidases (MAO-A and MAO-B). It is a major monoamine-metabolizing enzyme that is known to oxidize the neurotransmitter dopamine, along with other amines. MAO-B is primarily localized in astrocytes, while MAO-A is largely located in the neurons of the brain [5]. Selective, irreversible MAO-B inhibitors are devoid of the severe side effect called the “cheese effect” resulting from the non-selective MAOs that potentiate the effects of tyramine. Since MAO-B is significantly involved in the degradation of dopamine, it is considered to be a first-line therapy for PD [6]. Selegiline [5], rasagiline [5], and safinamide [7] are the only approved MAO-B inhibitors used in the treatment of PD [5,6,7,8]. Selegiline and rasagiline are examples of irreversible MAO-B inhibitors [8], while safinamide is a potent reversible MAO-B inhibitor [6]. Therefore, there is a need to develop novel selective MAO-B inhibitors as PD drugs.

Our previous research on the Central American medicinal plant, *Calea urticifolia*, yielded acacetin, a prominent dietary flavonoid [9,10] as a bioactive compound using bioassay-guided fractionation [9]. Acacetin exhibited good inhibitory activities against MAO-A (0.121 µM) and -B (0.049 µM). Later, acacetin 7-*O*-methyl ether proved to be a selective MAO-B inhibitor with a selectivity index (SI) of 505-fold for MAO A/B [11], which inspired further exploration of new MAO-B inhibitors using acacetin 7-*O*-methyl ether as the lead compound. Flavonoids are known for their admirable safety profile with no known notable adverse effects. Flavonoids possess various pharmacological properties, including anticancer, antioxidant, antimicrobial, cardioprotective, anti-inflammatory, and hepatoprotective activity [12].

In the current study, a series of acacetin 7-*O*-methyl ether derivatives were computationally designed using fragment-based drug design [13], synthesized, and evaluated as monoamine oxidase B inhibitors. Furthermore, molecular modeling studies were performed to understand the binding interactions.

## 2. Materials and Methods

### 2.1. General

^1^H and ^13^C NMR spectra were obtained using Bruker model AMX 400 and 500 NMR spectrometers operating at 400 and 500 MHz in ^1^H and 100 and 125 MHz in ^13^C, respectively. The chemical shift values were reported from tetramethylsilane (TMS) in parts per million (ppm) using known solvent chemical shifts. Standard pulse sequences were used for DEPT, COSY, HSQC, HMBC, NOESY, and TOCSY. Coupling constants are documented in hertz (Hz). The high-resolution mass spectra (HRMS) were recorded on a Waters Micromass Q-Tof Micro mass spectrometer with a lock spray source. The mass spectra (MS) were recorded on a WATERS ACQUITY Ultra Performance LC with a ZQ detector in ESI mode. If needed, the purity was determined using high-performance liquid chromatography (HPLC). The purity of all the final compounds was greater than 95%. Column chromatography was carried out on Sephadex LH-20 (GE Healthcare, Uppsala, Sweden) and silica gel (70–230 mesh, Merck, Billerica, MA, USA). The fractions from column chromatography were monitored using TLC (silica gel 60 F254, Merck, Billerica, MA, USA). Preparative TLC was carried out with silica gel 60 PF254 + 366 plates (20 × 20 cm, 1 mm thick, Analtech, Newark, DE, USA). Human recombinant monoamine oxidase A and monoamine oxidase B enzymes were obtained from BD Biosciences (Bedford, MA, USA). Kynuramine, clorgyline, deprenyl, acacetin, and DMSO were purchased from Sigma Chemical (St. Louis, MO, USA). All the acacetin 7-*O*-methyl ether analogs were synthesized in the Department of BioMolecular Sciences, Division of Medicinal Chemistry, School of Pharmacy, University of Mississippi, Oxford, MS, USA.

### 2.2. Synthesis of Derivatives

#### 2.2.1. Procedure for Preparation of Compounds **1**–**4**

##### Synthesis of (E) 4-Propyloxy-4′,6′-Dimethoxy-2′-Hydroxychalcone (**1a**)

IUPAC.: (*E*)-1-(2-Hydroxy-4,6-dimethoxyphenyl)-3-(4-propoxyphenyl)prop-2-en-1-one. A mixture of equimolar amounts of 2′-hydroxy-4′,6′-dimethoxyacetophenone (300 mg, 1.53 mmol) and 4-*n*-propoxybenzaldehyde (251 mg, 1.53 mmol) was taken in a round-bottomed flask (RBF) and 10 mL of 50% NaOH in ethanol for 16 h at rt. The resulting yellow precipitate was filtered and washed with Hex and CH_2_Cl_2_ to yield **1a** (342 mg, 65%). ^1^H NMR (400 MHz, CDCl_3_) δ_H_ 14.41 (s, C-2′-OH), 7.79 (s br, 2H, H-α and H-β), 7.55 (d, *J* = 8.5 Hz, 1H, H-2 and H-6), 6.91 (d, *J* = 8.5 Hz, 2H, H-3 and H-5), 6.10 (d, *J* = 1.8 Hz, 1H, H-3′), 5.96 (d, *J* = 1.8 Hz, 1H, H-5′), 3.97 (t, *J* = 6.5, 2H, H_2_-1″), 3.93 (s, 3H, OCH_3_), 3.85 (s, 3H, OCH_3_), 1.83 (dt, *J* = 6.5, 7.4 Hz, 2H, H_2_-2″), 1.05 (t, *J* = 7.4 Hz, 3H, CH_3_-3″). ^13^C NMR (100 MHz, CDCl_3_) δ_C_ 192.6 (C=O), 168.3 (C-2′), 166.0 (C-4′), 162.4 (C-6′), 161.0 (C-4), 142.6 (C-β), 130.1 (C-2 and C-6), 128.1 (C-1), 124.9 (C-α), 114.8 (C-3 and C-5), 106.3 (C-1′), 93.8 (C-3′), 91.2 (C-5′), 69.6 (C-1″), 55.8 (OCH_3_), 55.5 (OCH_3_), 22.5 (C-2″), 10.5 (C-3″). HRESIMS *m*/*z* 343.0897 [M+H]^+^ (calcd C_20_H_22_O_5_ 342.1467).

##### Synthesis of 5,7-Dimethoxy-4′-Propyloxyflavone (**1b**)

IUPAC.: 5,7-Dimethoxy-2-(4-propoxyphenyl)-4*H*-chromen-4-one. The chalcone **1a** (200 mg, 0.584 mmol) was treated with 3–4 mol% of iodine crystals with a minimal amount of DMSO at 140 °C for 4–6 h. The resulting white precipitate was extracted with CH_2_Cl_2_ and was removed by filtration. The filtrate was transferred into a separatory funnel where the layers were separated as aqueous and organic. The organic layer was washed with sodium thiosulfate (Na_2_S_2_O_3_), dried with sodium sulfate (Na_2_SO_4_), and concentrated. The intermediate flavonoid **1b** was then purified in a silica column using 100% CH_2_Cl_2_, 50:1 CH_2_Cl_2_-MeOH, 9:1 CH_2_Cl_2_-MeOH, 1:1 CH_2_Cl_2_-MeOH, and 100% MeOH (128 mg, 64%). ^1^H NMR (400 MHz, CDCl_3_) δ_H_ 7.80 (d, *J* = 8.9 Hz, 2H, H-2′ and H-6′), 6.98 (d, *J* = 8.9 Hz, 2H, H-3′ and H-5′), 6.59 (s, 1H, H-3), 6.57 (d, *J* = 2.3 Hz, 1H, H-6), 6.37 (d, *J* = 2.3 Hz, 1H, H-8), 3.99 (t, *J* = 6.6 Hz, 2H, H_2_-1″), 3.96 (s, OCH_3_), 3.91 (s, OCH_3_), 1.84 (dt, *J* = 6.5, 7.4 Hz, 2H, H_2_-2″), 1.06 (t, *J* = 7.4 Hz, 3H CH_3_-3″). ^13^C NMR (100 MHz, CDCl_3_) δ_C_ 177.7 (C-4), 163.9 (C-2), 161.6 (C-5), 160.9 (C-4′), 160.7 (C-9), 159.8 (C-7), 127.5 (C-2′ and C-6′), 123.5 (C-1′), 114.8 (C-3′ and C-5′), 109.2 (C-10), 107.6 (C-3), 96.0 (C-8), 92.8 (C-6), 69.7 (C-1″), 56.4 (OCH_3_), 55.7 (OCH_3_), 22.5 (C-2″), 10.5 (C-3″). HRESIMS *m*/*z* 341.0738 [M+H]^+^ (calcd C_20_H_20_O_5_ 340.1311).

##### Synthesis of 7-Methoxy-4′-Propyloxy-5-Hydroxyflavone (**1c**)

IUPAC.: 5-Hydroxy-7-methoxy-2-(4-propoxyphenyl)-4*H*-chromen-4-one. The intermediate **1b** (128 mg, 0.377 mmol) was treated with 1N boron tribromide (189 mg, 0.754 mmol) in the presence of CH_2_Cl_2_ at rt for 1–3 h. The reactant solution was diluted with 10:1 CH_2_Cl_2_:MeOH and then washed with brine and water. Na_2_SO_4_ was added to the solution and left overnight. The solution was then filtered, dried, and concentrated. The flavonoid **1c** was purified in a silica column using 30:1 CH_2_Cl_2_-MeOH (35 mg, 29%). ^1^H NMR (400 MHz, CDCl_3_) δ_H_ 12.83 (s, C-5-OH), 7.82 (d, *J* = 8.9 Hz, 2H, H-2′ and H-6′), 7.00 (d, *J* = 8.9 Hz, 2H, H-3′ and H-5′), 6.56 (s, 1H, H-3), 6.47 (d, *J* = 2.2 Hz, 1H, H-6), 6.35 (d, *J* = 2.2 Hz, 1H, H-8), 4.00 (t, *J* = 6.6 Hz, 2H, H_2_-1″), 3.88 (s, OCH_3_), 1.86 (ddd, *J* = 6.6, 7.4 Hz, 2H, H_2_-2″), 1.08 (t, *J* = 7.4 Hz, 3H, CH_3_-3″). ^13^C NMR (100 MHz, CDCl_3_) δ_C_ 182.4 (C-4), 165.4 (C-7), 164.0 (C-2), 162.2 (C-4′), 162.1 (C-5), 157.6 (C-9), 127.7 (C-2′ and C-6′), 123.5 (C-1′), 115.0 (C-3′ and C-5′), 105.5 (C-10), 104.2 (C-3), 98.0 (C-8), 92.5 (C-6), 69.8 (C-1″), 55.8 (OCH_3_), 22.5 (C-2″), 10.5 (C-3″). HRESIMS *m*/*z* 327.1038 [M+H]^+^ (calcd C_19_H_18_O_5_ 326.1154).

##### Synthesis of (E) 4-Isopropyloxy-4′,6′-Dimethoxy-2′-Hydroxychalcone (**2a**)

IUPAC.: (*E*)-1-(2-Hydroxy-4,6-dimethoxyphenyl)-3-(4-isopropoxyphenyl)prop-2-en-1-one. The procedure for the preparation of the chalcone **2a** is similar to that for the preparation of the chalcone **1a** except for the use of 4-isopropoxybenzaldehyde (251 mg, 1.53 mmol). The chalcone **2a** was purified in a silica column using 4:1 Hex-EtOAc, 3:1 Hex-EtOAc, 4:1 EtOAc-Hex, 100% EtOAc, and 100% MeOH (411 mg, 79%). ^1^H NMR (400 MHz, CDCl_3_) δ_H_ 14.43 (s, C-2′-OH), 7.80 (s br, 2H, H-α and H-β), 7.55 (d, *J* = 8.7 Hz, 2H, H-2 and H-6), 6.91 (d, *J* = 8.7 Hz, 2H, H-3 and H-5), 6.12 (d, *J* = 2.3, 1H, H-3′), 5.97 (d, *J* = 2.3, 1H, H-5′), 4.62 (sept, *J* = 6.0 Hz, 1H, H-1″), 3.93 (s, 3H, OCH_3_), 3.84 (s, 3H, OCH_3_), 1.37 (d, *J* = 6.0 Hz, 6H, CH_3_-2″ and CH_3_-3″). ^13^C NMR (100 MHz, CDCl_3_) δ_C_ 192.6 (C=O), 168.3 (C-2′), 166.0 (C-4′), 162.4 (C-6′), 159.8 (C-4), 142.6 (C-β), 130.1 (C-2 and C-6), 127.9 (C-1), 124.9 (C-α), 115.9 (C-3 and C-5), 106.4 (C-1′), 93.8 (C-3′), 91.2 (C-5′), 70.0 (C-1″), 55.8 (OCH_3_), 55.6 (OCH_3_), 22.0 (C-2″ and C-3″). HRESIMS *m*/*z* 343.0876 [M+H]^+^ (calcd C_20_H_22_O_5_ 342.1467).

##### Synthesis of 5,7-Dimethoxy-4′-Isopropyloxyflavone (**2b**)

IUPAC.: 2-(4-Isopropoxyphenyl)-5,7-dimethoxy-4*H*-chromen-4-one. This procedure is similar to that for the preparation of the intermediate flavonoid **1b**. The intermediate flavonoid **2b** was purified in a silica column using 1:1 Hex-EtOAc, 100% EtOAc, 20:1 EtOAc-MeOH, and 100% MeOH (78 mg, 52%). ^1^H NMR (400 MHz, CDCl_3_) δ_H_ 7.78 (d, *J* = 8.9 Hz, 2H, H-2′ and H-6′), 6.95 (d, *J* = 8.9, 2H, H-3′ and H-5′), 6.56 (s, 1H, H-3), 6.53 (d, *J* = 2.3, 1H, H-6), 6.34 (d, *J* = 2.3, 1H, H-8), 4.62 (sept, *J* = 6.0 Hz, 1H, H-1″), 3.93 (s, 3H, OCH_3_), 3.89 (s, 3H, OCH_3_), 1.36 (d, *J* = 6.0 Hz, 6H, CH_3_-2″ and CH_3_-3″). ^13^C NMR (100 MHz, CDCl_3_) δ_C_ 177.6 (C-4), 163.8 (C-2), 160.8 (C-5), 160.7 (C-4′), 160.5 (C-9), 159.8 (C-7), 127.6 (C-2′ and C-6′), 123.3 (C-1′), 115.8 (C-3′ and C-5′), 109.2 (C-3), 109.2 (C-10), 107.5 (C-3), 96.0 (C-8), 92.8 (C-6), 70.1 (C-1″), 56.4 (OCH_3_), 55.7 (OCH_3_), 21.9 (C-2″ and C-3″). HRESIMS *m*/*z* 341.0796 [M+H]^+^ (calcd C_20_H_20_O_5_ 340.1311).

##### Synthesis of 7-Methoxy-4′-Isopropyloxy-5-Hydroxyflavone (**2c**)

IUPAC.: 5-Hydroxy-2-(4-isopropoxyphenyl)-7-methoxy-4*H*-chromen-4-one. This procedure is similar to that for the preparation of the final flavonoid **1c**. The final flavonoid **2c** was purified in a silica column using 7:3 Hex-EtOAc and 1:1 Hex-EtOAc (6 mg, 10%). ^1^H NMR (400 MHz, CDCl_3_) δ_H_ 12.83 (s, C-5-OH), 7.83 (d, *J* = 8.9 Hz, 2H, H-2′ and H-6′), 6.99 (d, *J* = 8.9 Hz, 2H, H-3′ and H-5′), 6.58 (s, 1H, H-3), 6.49 (d, *J* = 2.2 Hz, 1H, H-6), 6.37 (d, *J* = 2.2 Hz, 1H, H-8), 4.66 (sept, *J* = 6.0 Hz, 1H, H-1″), 3.89 (s, 3H, OCH_3_), 1.40 (d, *J* = 6.0 Hz, 6H, CH_3_-2″ and CH_3_-3″). ^13^C NMR (100 MHz, CDCl_3_) δ_C_ 182.4 (C-4), 165.4 (C-7), 164.1 (C-2), 162.2 (C-4′), 161.1 (C-5), 157.3 (C-9), 128.0 (C-2′ and C-6′), 123.1 (C-1′), 115.9 (C-3′ and C-5′), 105.5 (C-10), 104.2 (C-3), 98.0 (C-8), 92.6 (C-6), 70.2 (C-1″), 55.8 (OCH_3_), 21.9 (C-2″ and C-3″). HRESIMS *m*/*z* 327.1038 [M+H]^+^ (calcd C_19_H_18_O_5_ 326.1154).

##### Synthesis of (E) 4-Isobutyloxy-4′,6′-Dimethoxy-2′-Hydroxychalcone (**3a**)

IUPAC.: (*E*)-1-(2-Hydroxy-4,6-dimethoxyphenyl)-3-(4-isobutoxyphenyl)prop-2-en-1-one. This procedure is similar to that for the preparation of the chalcone **1a** except for the use of 4-isobutoxy benzaldehyde (273 mg, 1.53 mmol). The chalcone **3a** was purified in a silica column using CH_2_Cl_2_ (436 mg, 80%). ^1^H NMR (500 MHz, CDCl_3_) δ_H_ 14.45 (s, C-2′-OH), 7.82 (s, br, 2H, H-α and H-β), 7.57 (d, *J* = 8.8 Hz, 2H, H-2 and H-6), 6.94 (d, *J* = 8.8 Hz, 2H, H-3 and H-5), 6.13 (d, *J* = 2.4 Hz, 1H, H-3′), 5.99 (d, *J* = 2.4 Hz, 1H, H-5′), 3.94 (s, 3H, OCH_3_), 3.86 (s, 3H, OCH_3_), 3.79 (d, *J* = 6.6 Hz, 2H, H_2_-1″), 2.13 (sept, *J* = 6.7 Hz, 1H, H-2″), 1.06 (d, *J* = 6.7 Hz, 6H, CH_3_-3″ and CH_3_-4″). ^13^C NMR (125 MHz, CDCl_3_) δ_C_ 192.6 (C=O), 168.4 (C-2′), 166.0 (C-4′), 162.4 (C-6′), 161.1 (C-4), 142.6 (C-β), 130.1 (C-2 and C-6), 128.0 (C-1), 124.9 (C-α), 114.9 (C-3 and C-5), 106.4 (C-1′), 93.8 (C-3′), 91.2 (C-5′), 74.5 (C-1″), 55.8 (OCH_3_), 55.6 (OCH_3_), 28.2 (C-2″), 19.2 (C-3″ and C-4″). HRESIMS *m*/*z* 357.0610 [M+H]^+^ (calcd C_21_H_24_O_5_ 356.1624).

##### Synthesis of 5,7-Dimethoxy-4′-Isobutyloxyflavone (**3b**)

IUPAC.: 2-(4-Isobutoxyphenyl)-5,7-dimethoxy-4*H*-chromen-4-one. This procedure is similar to that for the preparation of the intermediate flavonoid **1b**. The intermediate flavonoid **3b** was purified in a silica column using 1:1 Hex-EtOAc, 8:2 EtOAc-Hex, 100% EtOAc, 20:1 EtOAc, and 9:1 EtOAc-MeOH (113 mg, 57%). ^1^H NMR (400 MHz, CDCl_3_) δ_H_ 7.73 (d, *J* = 8.6 Hz, 2H, H-2′ and H-6′), 6.92 (d, *J* = 8.4 Hz, 2H, H-3′ and H-5′), 6.52 (s, 1H, H-3), 6.48 (d, *J* = 1.4 Hz, 1H, H-6), 6.29 (d, *J* = 1.6 Hz, 1H, H-8), 3.89 (s, 3H, OCH_3_), 3.85 (s, 3H, OCH_3_), 3.73 (d, *J* = 6.5 Hz, 2H, H_2_-1″), 2.07 (sept, *J* = 6.7 Hz, 1H, H-2″), 1.00 (d, *J* = 6.7 Hz, 6H, CH_3_-3″ and CH_3_-4″). ^13^C NMR (100 MHz, CDCl_3_) δ_C_ 177.6 (C-4), 163.8 (C-2), 161.7 (C-5), 160.7 (C-4′ and C-9), 159.7 (C-7), 127.4 (C-2′ and C-6′), 123.4 (C-1′), 114.8 (C-3′ and C-5′), 109.0 (C-10), 107.4 (C-3), 96.0 (C-8), 92.8 (C-6), 74.5 (C-1″), 56.3 (OCH_3_), 55.7 (OCH_3_), 28.2 (C-2″), 19.2 (C-3″ and C-4″). HRESIMS *m*/*z* 355.0958 [M+H]^+^ (calcd C_21_H_22_O_5_ 354.1467).

##### Synthesis of 7-Methoxy-4′-Isobutyloxy-5-Hydroxyflavone (**3c**)

IUPAC.: 5-Hydroxy-2-(4-Isobutoxyphenyl)7-methoxy-4*H*-chromen-4-one. This procedure is similar to that for the preparation of the final flavonoid **1c**. The final flavonoid **3c** was purified in a silica column using 7:3 EtOAc-Hex and 100% EtOAc (40 mg, 44%). ^1^H NMR (400 MHz, CDCl_3_) δ_H_ 12.79 (s, C-5-OH), 7.81 (d, *J* = 8.9 Hz, 2H, H-2′ and H-6′), 7.00 (d, *J* = 8.9 Hz, 2H, H-3′ and H-5′), 6.56 (s, 1H, H-3), 6.47 (d, *J* = 2.2 Hz, 1H, H-6), 6.36 (d, *J* = 2.2 Hz, 1H, H-8), 3.88 (s, 3H, OCH_3_), 3.80 (d, *J* = 6.6 Hz, 2H, H_2_-1″), 2.14 (sept, *J* = 6.6 Hz, 1H, CH_3_-2″), 1.07 (d, *J* = 6.7 Hz, 6H, CH_3_-3″ and CH_3_-4″). ^13^C NMR (100 MHz, CDCl_3_) δ_C_ 182.4 (C-4), 165.4 (C-7), 164.1 (C-2), 162.3 (C-4′), 162.1 (C-5), 157.6 (C-9), 127.9 (C-2′ and C-6′), 123.1 (C-1′), 114.9 (C-3′ and C-5′), 105.5 (C-10), 104.1 (C-3), 98.0 (C-8), 92.5 (C-6), 74.6 (C-1″), 55.7 (OCH_3_), 28.2 (C-2″), 19.2 (C-3″ and C-4″). HRESIMS *m*/*z* 341.0737 [M+H]^+^ (calcd C_20_H_20_O_5_ 340.1311).

##### Synthesis of (E) 4-Propargyloxy-4′,6′-Dimethoxy-2′-Hydroxychalcone (**4a**)

IUPAC.: (*E*)-1-(2-Hydroxy-4,6-dimethoxyphenyl)-3-(4-(prop-2-yn-1-yloxy)phenyl)prop-2-en-1-one. This procedure is similar to that for the preparation of the chalcone **1a** except for the use of 4-(prop-2-ynyloxy)benzaldehyde (245 mg, 1.53 mmol). The chalcone 4a was purified in a silica column using 100% CH_2_Cl_2_ (326 mg, 63%). ^1^H NMR (500 MHz, CDCl_3_) δ_H_ 14.38 (s, C-2′-OH), 7.84 (d, *J* = 15.6 Hz, 1H, H-β), 7.79 (d, *J* = 15.6 Hz, 1H, H-α), 7.60 (d, *J* = 8.8 Hz, 2H, H-2 and H-6), 7.03 (d, *J* = 8.8 Hz, 2H, H-3 and H-5), 6.14 (d, *J* = 2.3 Hz, 1H, H-3′), 5.99 (d, *J* = 2.3 Hz, 1H, H-5′), 4.76 (d, *J* = 2.4 Hz, 2H, H_2_-1″), 3.94 (s, 3H, OCH_3_), 3.86 (s, 3H, OCH_3_), 2.57 (t, *J* = 2.4 Hz, 1H, H-3″). ^13^C NMR (125 MHz, CDCl_3_) δ_C_ 192.6 (C=O), 168.4 (C-2′), 166.1 (C-4′), 162.5 (C-6′), 159.1 (C-4), 142.1 (C-β), 130.0 (C-2 and C-6), 129.2 (C-1), 125.7 (C-α), 115.3 (C-3 and C-5), 106.4 (C-1′), 93.8 (C-3′), 91.3 (C-5′), 78.1 (C-2″), 75.9 (C-3″), 55.9 (2C, OCH_3_, C-1″), 55.6 (OCH_3_). HRESIMS *m*/*z* 339.0599 [M+H]^+^ (calcd C_20_H_18_O_5_ 338.1154).

##### Synthesis of 5,7-Dimethoxy-4′-Propargyloxyflavone (**4b**)

IUPAC.: 5,7-Dimethoxy-2-(4-(prop-2-yn-1-yloxy)phenyl)-4*H*-chromen-4-one. This procedure is similar to that for the preparation of the intermediate flavonoid **1b**. The intermediate flavonoid **4b** was purified in a silica column using 1:1 Hex-EtOAc, 100% EtOAc, 20:1 EtOAc-MeOH, 9:1 EtOAc-MeOH, 1:1 EtOAc-MeOH, and 100% MeOH (87 mg, 54%). ^1^H NMR (400 MHz, CDCl_3_) δ_H_ 7.82 (d, *J* = 8.9 Hz, 2H, H-2′ and H-6′), 7.07 (d, *J* = 8.9 Hz, 2H, H-3′ and H-5′), 6.58 (s, 1H, H-3), 6.54 (d, *J* = 2.3 Hz, 1H, H-6), 6.36 (d, *J* = 2.3 Hz, 1H, H-8), 4.76 (d, *J* = 2.4 Hz, 2H, H_2_-1″), 3.94 (s, 3H, OCH_3_), 3.90 (s, 3H, OCH_3_), 2.57 (t, *J* = 2.4 Hz, 1H, H-3″). ^13^C NMR (100 MHz, CDCl_3_) δ_C_ 177.6 (C-4), 163.9 (C-2), 160.9 (C-5), 160.4 (C-4′), 159.8 (C-9), 159.8 (C-7), 127.5 (C-2′ and C-6′), 124.7 (C-1′), 115.2 (C-3′ and C-5′), 109.2 (C-10), 107.9 (C-3), 96.1 (C-8), 92.8 (C-6), 77.9 (C-2″), 76.1 (C-3″), 56.4 (C-1″), 55.9 (OCH_3_), 55.7 (OCH_3_). HRESIMS *m*/*z* 337.0495 [M+H]^+^ (calcd C_20_H_16_O_5_ 336.0998).

##### Synthesis of 7-Methoxy, 4′-Propargyloxy-5-Hydroxyflavone (**4c**)

IUPAC.: 5-Hydroxy-7-methoxy-2-(4-(prop-2-yn-1-yloxy)phenyl)-4*H*-chromen-4-one. This procedure is similar to that for the preparation of the final flavonoid **1c**. The final flavonoid **4c** was purified in a silica column with 7:3 Hex-EtOAc, 1:1 Hex-EtOAc, 100% EtOAc, 20:1 EtOAc-MeOH, and 9:1 EtOAc-MeOH (14 mg, 28%). ^1^H NMR (400 MHz, CDCl_3_) δ_H_ 12.79 (s, C-5-OH), 7.88 (d, *J* = 8.9 Hz, 2H, H-2′ and H-6′), 7.12 (d, *J* = 8.9 Hz, 2H, H-3′ and H-5′), 6.60 (s, 1H, H-3), 6.50 (d, *J* = 2.2 Hz, 1H, H-6), 6.39 (d, *J* = 2.2 Hz, 1H, H-8), 4.80 (d, *J* = 2.3 Hz, 2H, H_2_-1″), 3.90 (s, 3H, OCH_3_), 2.59 (t, *J* = 2.3 Hz, 1H, H-3″). ^13^C NMR (100 MHz, CDCl_3_) δ_C_ 182.4 (C-4), 165.5 (C-7), 163.8 (C-2), 162.2 (C-4′), 160.4 (C-5), 157.7 (C-9), 128.0 (C-2′ and C-6′), 124.4 (C-1′), 115.4 (C-3′ and C-5′), 105.6 (C-10), 104.6 (C-3), 98.1 (C-8), 92.6 (C-6), 77.7 (C-2″), 76.2 (C-3″), 55.9 (C-1″), 55.8 (OCH_3_). HRESIMS *m*/*z* 323.0767 [M+H]^+^ (calcd C_19_H_14_O_5_ 322.0841).

#### 2.2.2. Procedure for Preparation of Compounds **5**

##### Synthesis of 4-(Cyclopropyl Methoxy)Benzaldehyde (Compound IV)

IUPAC.: 4-(Cyclopropylmethoxy)benzaldehyde. (Bromomethyl)cyclopropane (400 mg, 2.96 mmol) was added to 4-hydroxy benzaldehyde (724 mg, 5.92 mmol) and was dissolved in 15 mL of acetone and potassium carbonate (1.6 g, 11.9 mmol). The mixture was then refluxed for 36 h, then diluted with EtOAc and washed with water. This compound IV was purified in a silica column using 95:5 Hex-EtOAc (380 mg, 73%). ^1^H NMR (400 MHz, CDCl_3_) δ_H_ 9.82 (s, 1H, CHO), 7.76 (d, *J* = 8.8 Hz, 2H, H-2 and H-6), 6.94 (d, *J* = 8.8 Hz, 2H, H-3 and H-5), 3.84 (dd, *J* = 8.1, 10.9 Hz, 2H, H_2_-1′), 1.27–1.21 (m, 1H, H-2′), 0.64 – 0.61 (m, 2H, H-3′a and H-4′a), 0.34 – 0.32 (m, 2H, H-3′b and H-4′b). ^13^C NMR (100 MHz, CDCl_3_) δ_C_ 190.7 (CHO), 164.0 (C-4), 131.9 (C-2 and C-6), 129.8 (C-1), 114.8 (C-3 and C-5), 73.0 (C-1′), 10.0 (C-2′), 3.2 (C-3′ and C-4′).

##### Synthesis of (E) 4-(Cyclopropyl Methoxy)-4′,6′-Dimethoxy-2′-Hydroxychalcone (**5a**)

IUPAC.: (*E*)-3-(4-(Cyclopropylmethoxy)phenyl)-1-(2-hydroxy-4,6-dimethoxyphenyl)prop-2-en-1-one. This procedure is similar to that for the preparation of the chalcone **1a** except for the use of 4-(Cyclopropylmethoxy)benzaldehyde (compound IV) (270 mg, 1.53 mmol). The chalcone **5a** was purified in a silica column with 4:1 CH_2_Cl_2_-Hex, 100% CH_2_Cl_2_, 20:1 CH_2_Cl_2_-MeOH, and 9:1 CH_2_Cl_2_-MeOH (287 mg, 53%). ^1^H NMR (400 MHz, CDCl_3_) δ_H_ 14.42 (s, C-2′-OH), 7.84 (s, br, 2H, H-α and H-β), 7.55 (d, *J* = 8.7 Hz, 2H, H-2 and H-6), 6.93 (d, *J* = 8.7 Hz, 2H, H-3 and H-5), 6.11 (d, *J* = 2.3 Hz, 1H, H-3′), 5.97 (d, *J* = 2.3 Hz, 1H, H-5′), 3.86 (s, 2H, H_2_-1″), 3.92 (s, 3H, OCH_3_), 3.84 (s, 3H, OCH_3_), 1.31 – 1.27 (m, 1H, H-2″), 0.67 (dd, *J* = 4.9, 12.9 Hz, 2H, H-3″a and H-4″a), 0.38 (dd, *J* = 4.9, 10.3 Hz, 2H, H-3″b and H-4″b). ^13^C NMR (100 MHz, CDCl_3_) δ_C_ 192.6 (C=O), 168.3 (C-2′), 166.0 (C-4′), 162.4 (C-6′), 160.8 (C-4), 142.5 (C-β), 130.1 (C-2 and C-6), 128.2 (C-1), 125.0 (C-α), 114.9 (C-3 and C-5), 106.3 (C-1′), 93.8 (C-3′), 91.2 (C-5′), 72.9 (C-1″), 55.8 (OCH_3_), 55.5 (OCH_3_), 10.2 (C-2″), 3.2 (C-3″ and C-4″). HRESIMS *m*/*z* 355.0575 [M+H]^+^ (calcd C_21_H_22_O_5_ 354.1467).

##### Synthesis of 5,7-Dimethoxy-4′(Cyclopropyl Methoxy)Flavone (**5b**)

IUPAC.: 2-(4-(Cyclopropylmethoxy)phenyl)-5,7-dimethoxy-4*H*-chromen-4-one. This procedure is similar to that for the preparation of the intermediate flavonoid 1b. The intermediate flavonoid **5b** was purified in a silica column using 1:1 Hex-EtOAc, 100% EtOAc, 20:1 EtOAc-MeOH, 9:1 EtOAc-MeOH, and 1:1 EtOAc-MeOH (120 mg, 78%). ^1^H NMR (400 MHz, CDCl_3_) δ_H_ 7.78 (d, *J* = 9.0 Hz, 2H, H-2′ and H-6′), 6.97 (d, *J* = 9.0 Hz, 2H, H-3′ and H-5′), 6.56 (s, 1H, H-3), 6.53 (d, *J* = 2.3 Hz, 1H, H-6), 6.34 (d, *J* = 2.3 Hz, 1H, H-8), 3.93 (s, 3H, OCH_3_), 3.89 (s, 3H, OCH_3_), 3.85 (d, *J* = 6.9 Hz, 2H, H_2_-1″), 1.25 (t, *J* = 7.2, 1H, H-2″), 0.66 (dd, *J* = 4.8, 12.9 Hz, 2H, H-3″a and H-4″a), 0.37 (dd, *J* = 4.8, 10.6 Hz, 2H, H-3″b and H-4″b). ^13^C NMR (100 MHz, CDCl_3_) δ_C_ 177.6 (C-4), 163.9 (C-2), 161.5 (C-5), 160.8 (C-4′), 160.7 (C-9), 159.8 (C-7), 127.5 (C-2′ and C-6′), 123.6 (C-1′), 114.9 (C-3′ and C-5′), 109.1 (C-10), 107.5 (C-3), 96.0 (C-8), 92.8 (C-6), 72.9 (C-1″), 56.4 (OCH_3_), 55.7 (OCH_3_), 10.1 (C-2″), 3.2 (C-3″ and C-4″). HRESIMS *m*/*z* 353.0805 [M+H]^+^ (calcd C_21_H_20_O_5_ 352.1311).

#### 2.2.3. Procedure for Preparation of Compounds **6**–**8**

##### Synthesis of (E) 4-Bromo-4′,6′-Dimethoxy-2′-Hydroxychalcone (**6a**)

IUPAC.: (*E*)-3-(4-Bromophenyl)-1-(2-hydroxy-4,6-dimethoxyphenyl)prop-2-en-1-one. A mixture of equimolar amounts of 2′-hydroxy-4′,6′-dimethoxyacetophenone (300 mg, 1.53 mmol) and 4-bromobenzaldehyde (285 mg, 1.53 mmol) was taken in a RBF and 10 mL of 50% NaOH in ethanol was added and left for 16 h at rt. The resulting yellow precipitate was then suction filtered and washed with hexanes and CH_2_Cl_2_. The brominated chalcone **6a** was then purified in a silica column using 10% EtOAc and hexanes (361 mg, 65%). ^1^H NMR (400 MHz, CDCl_3_) δ_H_ 14.23 (s, C-2′-OH), 7.89 (d, *J* = 15.6 Hz, 1H, H-β), 7.71 (d, *J* = 15.6 Hz, 1H, H-α), 7.55 (d, *J* = 8.4 Hz, 2H, H-2 and H-6), 7.47 (d, *J* = 8.4 Hz, 2H, H-3 and H-5), 6.13 (d, *J* = 2.2 Hz, 1H, H-3′), 5.98 (d, *J* = 2.2 Hz, 1H, H-5′), 3.93 (s, 3H, OCH_3_), 3.86 (s, 3H, OCH_3_). ^13^C NMR (100 MHz, CDCl_3_) δ_C_ 192.3 (C=O), 168.4 (C-4′), 166.4 (C-6′), 162.5 (C-2′), 140.8 (C-β), 134.5 (C-1), 132.1 (C-3 and C-5), 129.7 (C-2 and C-6), 128.1 (C-α), 124.2 (C-4), 106.3 (C-1′), 93.8 (C-3′), 91.3 (C-5′), 55.9 (OCH_3_), 55.6 (OCH_3_). HRESIMS *m*/*z* 363.1418 [M+H]^+^ (calcd C_17_H_15_BrO_4_ 362.0154).

##### Synthesis of 5,7-Dimethoxy-4′-Bromoflavone (**6b**)

IUPAC.: 2-(4-Bromophenyl)-5,7-dimethoxy-4*H*-chromen-4-one. The brominated chalcone (**6a**) (200 mg, 0.551 mmol) was treated with 3–4 mol% of iodine crystals with a minimal amount of DMSO at 140 °C for 4–6 h. The resulting white precipitate was extracted with CH_2_Cl_2_ and was removed by filtration. The filtrate was transferred into a separatory funnel, where the layers were separated as aqueous and organic. The organic layer was washed with Na_2_S_2_O_3_, dried with Na_2_SO_4_, and concentrated. The intermediate flavonoid **6b** was purified in a silica column with 1:1 Hex-EtOAc, 3:2 EtOAc-Hex, 4:1 EtOAc-Hex, and 9:1 EtOAc-Hex (134 mg, 67%). ^1^H NMR (400 MHz, CDCl_3_) δ_H_ 7.72 (d, *J* = 8.7 Hz, 2H, H-3′ and H-5′), 7.62 (d, *J* = 8.7 Hz, 2H, H-2′ and H-6′), 6.64 (s, 1H, H-3), 6.55 (d, *J* = 2.3 Hz, 1H, H-6), 6.37 (d, *J =* 2.3 Hz, 1H, H-8), 3.95 (s, 3H, OCH_3_), 3.91 (s, 3H, OCH_3_). ^13^C NMR (100 MHz, CDCl_3_) δ_C_ 177.3 (C-4), 164.2 (C-2), 160.9 (C-5), 159.8 (C-9), 159.5 (C-7), 132.2 (C-3′ and C-5′), 130.4 (C-1′), 127.3 (C-2′ and C-6′), 125.8 (C-4′), 109.2 (C-10 and C-3), 96.3 (C-8), 92.8 (C-6), 56.4 (OCH_3_), 55.8 (OCH_3_). HRESIMS *m*/*z* 361.1324 [M+H]^+^ (calcd C_17_H_13_BrO_4_ 359.9997).

##### Synthesis of 5,7-Dimethoxy-4′-Aminopropylflavone (**6c**)

IUPAC.: 5,7-Dimethoxy-2-(4-(propylamino)phenyl)-4*H*-chromen-4-one. Sodium *tert*-butoxide (17 mg, 0.18 mmol), tris(dibenzylideneacetone)dipalladium (11 mg, 0.012 mmol), and 1,1′-binaphthalene-2,2′-diylbis(diphenylphosphine) (8 mg, 0.012 mmol) were dissolved in 5 mL of toluene. To this, the brominated intermediate flavonoid **6b** (50 mg, 0.138 mmol) and propylamine (11 mg, 0.18 mmol) were added dropwise with stirring at rt, and the mixture was refluxed at 80 °C for 48 h. The nitrogenated intermediate flavonoid was purified in a silica column using 7:3 EtOAc-Hex, 9:1 EtOAc-Hex, 100% EtOAc, and 9:1 EtOAc-MeOH (24 mg, 52%). ^1^H NMR (500 MHz, CDCl_3_) δ_H_ 7.67 (d, *J* = 10.9 Hz, 2H, H-2′ and H-6′), 6.61 (d, *J* = 10.9 Hz, 2H, H-3′ and H-5′), 6.52 (d, *J* = 2.6 Hz, 1H, H-6), 6.50 (s, 1H, H-3), 6.33 (d, *J* = 2.5 Hz, 1H, H-8), 3.92 (s, 3H, OCH_3_), 3.88 (s, 3H, OCH_3_), 3.13 (t, *J* = 9.0 Hz, 2H, H_2_-1″), 1.66 (ddd, *J* = 9.2, 18.2, 9.0 Hz, 2H, H_2_-2″), 1.00 (t, *J* = 9.2 Hz, 3H, CH_3_-3″). ^13^C NMR (125 MHz, CDCl_3_) δ_C_ 177.8 (C-4), 163.6 (C-2), 161.6 (C-5), 160.7 (C-4′), 159.7 (C-9), 150.9 (C-7), 127.5 (C-2′ and C-6′), 119.0 (C-1′), 112.1 (C-3′ and C-5′), 109.2 (C-10), 105.8 (C-3), 95.8 (C-8), 92.8 (C-6), 56.3 (OCH_3_), 55.7 (OCH_3_), 45.2 (C-1″), 22.5 (C-2″), 11.5 (C-3″). HRESIMS *m*/*z* 340.2991 [M+H]^+^ (calcd C_20_H_21_NO_4_ 339.1471).

##### Synthesis of 7-Methoxy-4′-Aminopropyl-5-Hydroxyflavone (**6d**)

IUPAC.: 5-Hydroxy-7-methoxy-2-(4-(propylamino)phenyl)-4*H*-chromen-4-one. The nitrogenated intermediate flavonoid **6c** (15 mg, 0.044 mmol) was treated with double the moles of 1N boron tribromide (22 mg, 0.088 mmol) in the presence of CH_2_Cl_2_ at rt for 1–3 h. The reactant solution was diluted with 10:1 CH_2_Cl_2_:MeOH and then washed with brine and water. Na_2_SO_4_ was added to the solution and left overnight. The solution was then filtered into a RBF, dried, and concentrated. The final compound **6d** was purified in a silica column using 1:1 Hex-EtOAc (11 mg, 77%). ^1^H NMR (500 MHz, CDCl_3_) δ_H_ 12.90 (s, C-5-OH), 7.65 (d, *J* = 8.8 Hz, 2H, H-2′ and H-6′), 6.58 (d, *J* = 8.7 Hz, 2H, H-3′ and H-5′), 6.43 (s, 1H, H-3), 6.39 (d, *J* = 2.2 Hz, 1H, H-6), 6.27 (d, *J* = 2.2 Hz, 1H, H-8), 3.80 (s, 3H, OCH_3_), 3.10 (t, *J* = 7.2 Hz, 2H, H_2_-1″), 1.62 (ddd, *J* = 7.4, 7.2, 7.3 Hz, 2H, H_2_-2″), 0.96 (t, *J* = 7.4 Hz, 3H, CH_3_-3″). ^13^C NMR (125 MHz, CDCl_3_) δ_C_ 182.3 (C-4), 165.2 (C-7), 164.4 (C-2), 162.1 (C-5 and C-4′), 157.6 (C-9), 128.0 (C-2′ and C-6′), 127.5 (C-1′), 114.0 (C-3′ and C-5′), 105.4 (C-10), 103.0 (C-3), 97.9 (C-8), 92.5 (C-6), 55.8 (OCH_3_), 29.7 (C-1″), 22.1 (C-2″), 11.5 (C-3″). HRESIMS *m*/*z* 326.2691 [M+H]^+^ (calcd C_19_H_19_NO_4_ 325.1314).

##### Synthesis of 5,7-Dimethoxy-4′-Aminoisopropylflavone (**7c**)

IUPAC.: 2-(4-(Isopropylamino)phenyl)-5,7-dimethoxy-4*H*-chromen-4-one. This procedure is similar to that for the preparation of the intermediate flavonoid **6c** except for the use of isopropylamine (11 mg, 0.18 mmol). The intermediate flavonoid **7c** was purified in a silica column using 9:1 EtOAc-Hex (37 mg, 79%). ^1^H NMR (400 MHz, CDCl_3_) 7.65 (d, *J* = 8.7 Hz, 2H, H-2′ and H-6′), 6.58 (d, *J* = 8.7 Hz, 2H, H-3′ and H-5′), 6.50 (d, *J* = 2.2 Hz, 1H, H-6), 6.48 (s, 1H, H-3), 6.31 (d, *J* = 2.2 Hz, 1H, H-8), 3.90 (s, 3H, OCH_3_), 3.86 (s, 3H, OCH_3_), 3.67 (sept, *J* = 6.4 Hz, 1H, H-1″), 1.22 (d, *J* = 6.4 Hz, 6H, H-2″ and H-3″). ^13^C NMR (100 MHz, CDCl_3_) δ_C_ 177.7 (C-4), 163.6 (C-2), 161.5 (C-5), 160.7 (C-4′), 159.7 (C-9), 150.0 (C-7), 127.5 (C-2′ and C-6′), 118.8 (C-1′), 112.5 (C-3′ and C-5′), 109.1 (C-10), 105.7 (C-3), 95.8 (C-8), 92.8 (C-6), 56.3 (OCH_3_), 55.6 (OCH_3_), 43.9 (C-1″), 22.7 (C-2″ and C-3″). HRESIMS *m*/*z* 340.3005 [M+H]^+^ (calcd C_20_H_21_NO_4_ 339.1471).

##### Synthesis of 7-Methoxy-4′-Aminoisopropyl-5-Hydroxyflavone (**7d**)

IUPAC.: 5-Hydroxy-2-(4-(Isopropylamino)phenyl)-7-methoxy-4*H*-chromen-4-one. This procedure is similar to that for the preparation of the final compound **6d**. The final compound **7d** was purified in a silica column with 9:1 Hex-EtOAc (16 mg, 83%). ^1^H NMR (500 MHz, CDCl_3_) 13.00 (s, C-5-OH), 7.74 (d, *J* = 8.9 Hz, 2H, H-2′ and H-6′), 6.64 (d, *J* = 8.9 Hz, 2H, H-3′ and H-5′), 6.52 (s, 1H, H-3), 6.48 (d, *J* = 2.3 Hz, 1H, H-6), 6.37 (d, *J* = 2.3 Hz, 1H, H-8), 3.90 (s, 3H, OCH_3_), 3.75 (sept, *J* = 6.3 Hz, 1H, H-1″), 1.29 (d, *J* = 6.3 Hz, 6H, CH_3_-2″ and CH_3_-3″). ^13^C NMR (125 MHz, CDCl_3_) δ_C_ 182.4 (C-4), 165.1 (C-7), 164.9 (C-2), 162.1 (C-4′), 157.6 (C-5), 150.5 (C-9), 128.1 (C-2′ and C-6′), 118.6 (C-1′), 112.6 (C-3′ and C-5′), 105.5 (C-10), 102.4 (C-3), 97.8 (C-8), 92.5 (C-6), 55.7 (OCH_3_), 44.1 (C-1″), 22.8 (C-2″ and C-3″). HRESIMS *m*/*z* 326.2691 [M+H]^+^ (calcd C_19_H_19_NO_4_ 325.1314).

##### Synthesis of 5,7-Dimethoxy-4′-(Cyclopropyl Methylamino)Flavone (**8c**)

IUPAC.: 2-(4-((Cyclopropylmethyl)amino)phenyl)-5,7-dimethoxy-4*H*-chromen-4-one. This procedure is similar to that for the preparation of the intermediate flavonoid **6c** except for the use of (aminomethyl)cyclopropane (13 mg, 0.18 mmol). The intermediate flavonoid **8c** was purified in a silica column using 7:3 EtOAc-Hex, 100% EtOAc, 10:1 EtOAc-MeOH, and 100% MeOH (40 mg, 83%). ^1^H NMR (500 MHz, CDCl_3_) 7.66 (d, *J* = 8.5 Hz, 2H, H-2′ and H-6′), 6.66 (d, *J* = 8.7 Hz, 2H, H-3′ and H-5′), 6.56 (s, 1H, H-3), 6.41 (d, *J* = 2.2 Hz, 1H, H-6), 6.38 (d, *J* = 2.2 Hz, 1H, H-8), 3.97 (s, 3H, OCH_3_), 3.92 (s, 3H, OCH_3_), 3.05 (d, *J* = 6.9 Hz, 2H, H_2_-1″), 1.12 (m, 1H, H-2″), 0.61 (dd, *J* = 6.0, 13.6 Hz, 2H, H-3″a and H-4″a), 0.30 (dd, *J* = 5.5, 10.5 Hz, 2H, H-3″b and H-4″b). ^13^C NMR (125 MHz, CDCl_3_) δ_C_ 177.8 (C-4), 163.7 (C-2), 161.5 (C-5), 160.8 (C-4′), 160.9 (C-9), 159.8 (C-7), 127.5 (C-2′ and C-6′), 119.3 (C-1′), 112.3 (C-3′ and C-5′), 109.3 (C-10), 106.0 (C-3), 96.3 (C-8), 92.8 (C-6), 56.5 (OCH_3_), 55.7 (OCH_3_), 48.5 (C-1″), 10.7 (C-2″), 3.6 (C-3″ and C-4″). HRESIMS *m*/*z* 352.2586 [M+H]^+^ (calcd C_21_H_21_NO_4_ 351.1471).

##### Synthesis of 7-Methoxy-4′-(Cyclopropyl Methylamino)-5-Hydroxyflavone (**8d**)

2-(4-((Cyclopropylmethyl)amino)phenyl)-5-hydroxy-7-methoxy-4*H*-chromen-4-one. This procedure is similar to that for the preparation of the final compound **6d**. The final compound **8d** was purified in a sephadex column with 1:1 Hex-EtOAC (2 mg, 7%). ^1^H NMR (500 MHz, CDCl_3_) 13.00 (s, C-5-OH), 7.75 (d, *J* = 8.8 Hz, 2H, H-2′ and H-6′), 6.67 (d, *J* = 8.8 Hz, 2H, H-3′ and H-5′), 6.53 (s, 1H, H-3), 6.48 (d, *J* = 2.1 Hz, 1H, H-6), 6.37 (d, *J* = 2.1 Hz, 1H, H-8), 3.90 (s, 3H, OCH_3_), 3.07 (d, *J* = 6.9 Hz, 2H, H_2_-1″), 1.14 (m, 1H, H-2″), 0.63 (dd, *J* = 5.9, 13.0 Hz, 2H, H-3″a and H-4″a), 0.31 (dd, *J* = 4.9, 9.9 Hz, 2H, H-3″b and H-4″b). ^13^C NMR (125 MHz, CDCl_3_) δ_C_ 182.4 (C-4), 165.1 (C-7), 164.9 (C-2), 162.1 (C-4′), 157.6 (C-5), 151.3 (C-9), 128.1 (C-2′ and C-6′), 118.9 (C-1′), 112.3 (C-3′ and C-5′), 105.5 (C-10), 102.5 (C-3), 97.8 (C-8), 92.5 (C-6), 55.7 (OCH_3_), 48.4 (C-1″), 10.7 (C-2″), 3.6 (C-3″ and C-4″). HRESIMS *m*/*z* 338.2880 [M+H]^+^ (calcd C_20_H_19_NO_4_ 337.1314).

### 2.3. Evaluation of Pan Assay Interference Compounds (PAINS)

All the designed MAO-B inhibitors were analyzed per the recently published editorial [14] using the False Positive Remover [15] and the ZINC Pattern Identifier [16]. All compounds passed the filter and were not reported as covalent inhibitors or potential PAINS by any of these algorithms.

### 2.4. Monoamine Oxidase Inhibition Assay and Determination of IC_50_ Values for Synthesized Compounds

To investigate the inhibitory effect of the acacetin 7-*O*-methyl ether analogs on human recombinant MAO-A and MAO-B, the kynuramine oxidation deamination assay was performed in 384-well plates as previously reported, with minor modifications [17]. A fixed single concentration of kynuramine substrate and varying concentrations of the test inhibitor were used to determine the IC_50_ values. The kynuramine concentrations for MAO-A and -B were 80 μM and 50 μM, respectively. These concentrations of kynuramine were twice the apparent K_M_ value for substrate binding [9,18]. The acacetin 7-*O*-methyl ether analogs were tested at the concentrations ranging from 0.001 μM to 100 μM for MAO-A and -B inhibition assays. Enzymatic reactions were performed in 50 μL of the assay mixture containing 0.1 M potassium phosphate buffer, pH 7.4. The inhibitors and acacetin 7-*O*-methyl ether analogs were dissolved in DMSO, diluted in the buffer solution, and pre-incubated at 37 °C for 10 min (the final concentration of DMSO was <1.0%). Reactions were initiated by the addition of 12.50 μL of MAO-A (to 5 μg/mL) or -B (to 10 μg/mL). The plate was incubated for 20 min at 37 °C, and the enzymatic reaction was terminated by the addition of 18.8 μL of 2N NaOH. Formation of the enzyme product, 4-hydroxyquinoline, was measured fluorometrically using a SpectraMax M5 fluorescence plate reader (Molecular Devices, Sunnyvale, CA, USA) with excitation (320 nm) and emission (380 nm) wavelengths and the Soft Max Pro program. The inhibition effects of enzyme activity were calculated as percent of product formation compared to the corresponding control (enzyme–substrate reaction) without inhibitors. Controls included the assays where the enzyme or the substrate was added after terminating the reaction to determine the interference with the fluorescence measurements. The IC_50_ values for MAO-A and -B inhibition were calculated from the concentration-dependent inhibition curves using XLFit^®^ software.

The enzyme assay was performed at a fixed concentration of the substrate kynuramine (80 μM for MAO-A and 50 μM for MAO-B) and varying concentrations of the inhibitor/test analogs (0.01 μM to 100 μM) for MAO-A and (0.01 μM to 100 μM) for MAO-B. The dose–response curves were generated using Microsoft^®^ Excel and the IC_50_ values were calculated using XLfit software [9].

### 2.5. Enzyme Kinetics, Mechanism Studies, Analysis of Reversibility, and Binding Assays of Acacetin 7-O-Methyl Ether Analogs

For determination of the binding affinity of the inhibitor (*Ki*) with MAO-B, the enzyme assays were carried out at different concentrations of kynuramine substrate (1.90 μM to 500 μM) and varying concentrations of the test compound. Acacetin 7-*O*-methyl ether analogs: **1c**, **2c**, **3c**, and **4c** were tested at 0.015–0.500 μM for MAO-B to determine the *K_m_* and *V_max_* values of the enzymes in the presence of the inhibitor. The controls without inhibitor were also run simultaneously. Three sets of assays were performed at varying concentrations of the substrate for each experiment: one control without inhibitor and the others at two fixed concentrations of the inhibitor. The data were analyzed by double reciprocal Lineweaver–Burk plots to determine *Ki* (i.e., inhibition/binding affinity) values and the kinetic data, namely *K*m, *V*max, and *Ki* values, were computed by SigmaPlot 12 [9].

Most of the inhibitors produce inhibition of the target enzyme activity through formation of an enzyme–inhibitor complex. Formation of the enzyme–inhibitor complex may be accelerated in the presence of a high concentration of the inhibitor. The reversibility/irreversibility of binding of acacetin 7-*O*-methyl ether analogs with MAO-B was determined from the formation of the complex by incubating the enzyme with a high concentration of the inhibitor followed by extensive equilibrium-dialysis of the enzyme–inhibitor complex. Recovery of the catalytic activity of the enzyme was determined by assay of the enzyme activity before and after equilibrium-dialysis. MAO-B (0.2 mg/mL protein) enzyme was incubated with each analog, namely acacetin (0.5 μM), **1c** (1.5 μM), **2c** (1.5 μM), **3c** (1.5 μM), **4c** (1.5 μM), and deprenyl (0.5 μM), in a total volume of 1 mL of potassium phosphate buffer (100 mM, pH 7.4). After 20 min of incubation at 37 °C, the reaction was stopped by chilling the tubes in the ice bath. All the samples were dialyzed against potassium phosphate buffer (25 mM; pH 7.4) at 4 °C for 14–18 h (with three buffer changes). The control enzyme (without inhibitor) was also run simultaneously using the same procedure and the activity of the enzyme was determined before and after the dialysis.

To analyze if the nature of MAO-B binding to the inhibitor was time-dependent, the enzyme was pre-incubated for different time periods (0–15 min) with the inhibitor concentrations, which exhibited approximately 70–80% inhibition. The inhibitor concentrations used to test time-dependent inhibition were acacetin (0.080 μM), **1c** (0.100 μM), **2c** (0.300 μM), **3c** (0.300 μM), **4c** (0.400 μM), and deprenyl (0.080 μM) with MAO-B (12.5 μg/mL). The controls, without inhibitors, were also run simultaneously. Activities of the enzyme were determined as described above, and the percentage of the remaining enzyme activity was plotted against the pre-incubation time to determine time-dependent inhibition.

### 2.6. Molecular Modeling Studies

The crystal structures of MAO-A (PDB ID: 2Z5Y with an atomic resolution of 2.17 Å) and MAO-B (PDB ID: 4A79 with an atomic resolution of 1.89 Å) were downloaded from the protein databank (www.rcsb.org, accessed on 12 January 2017). The protein structural files were prepared using the protein preparation wizard of Schrödinger suite [19]. Standard procedures were followed for protein preparation through assigning bond orders, adding hydrogens and removing original ones, creating zero-order bonds to metals, filling in the missing side chains, and deleting waters beyond 5 Å from the ligand. The ionization states were generated at pH 7.4 using Epik [20,21,22]. The next step was sampling water orientations and checking for possible protonation and tautomerization states at pH 7.4. Water molecules that did not display at least two hydrogen bonds with no-water residues were deleted. Finally, the structure was relaxed to remove all atomic clashes using OPLS3 force field [23].

The receptor grid was generated using Glide [24,25,26,27]. The binding pocket was identified by the ligand coordinates. Because of the importance of the FAD cofactor in directing the right pose in the substrate binding pocket [28,29,30], it was kept during the construction of the docking grids. The dimensions of the docking grid of MAO-A are: a grid center of −40.89 × −26.66 × −14.85, an inner box of 10 × 10 × 10, and an outer box of 22.59 × 22.59 × 22.59 (Å). The dimensions of the MAO-B grid are: a grid center of 14.02 × 130.51 × 25.58, an inner box of 10 × 10 × 10, and an outer box of 26.74 × 26.74 × 26.74 (Å). The soft docking protocol was followed by softening the potential of nonpolar parts of the receptor (i.e., decreasing the van der Waals radius of nonpolar atoms to accommodate ligands). Ligands were prepared for docking using LigPrep [31]. This step was performed for acacetin 7-*O*-methyl ether and for the designed compounds as well. Possible protonation and tautomerization states were generated at a pH of 7.4 and the specified chiralities were retained. The lowest energy conformer of each ligand was kept. OPLS force field was employed in the preparation step. Acacetin 7-*O*-methyl ether and the designed compounds were docked into the receptor grids of MAO-A/B. The soft docking protocol [32] was followed by using flexible ligand sampling and the soft receptor grid. Standard docking precision was used to generate the best five poses for acacetin 7-*O*-methyl ether and the best pose for the other compounds.

The two most diverse poses of acacetin 7-*O*-methyl ether were used for molecular dynamics simulations and mapping of the active site water molecules. Each complex was prepared and solvated in an orthorhombic box of the TIP4P water solvation model using Desmond system builder [33,34]. The net charge on the protein was neutralized by adding the appropriate number of sodium ions. MD simulations were conducted using OPLS3 force field. The FAD cofactor was included in the MD studies and it was prepared by OPLS3 force field and charges. The cofactor showed a state penalty of 0.65 kcal/mol and a hydrogen bond count of 16 at a negative charge of −2. Desmond’s minimization algorithm was used to relax the MD system for 2000 iterations. Desmond’s algorithm involves a series of energy minimizations and short MD simulations to further relax the solvated protein before the MD production step, including two minimization steps and 4 short MD simulation steps for 12 ps, 12 ps, 24 ps, and 24 ps at 10 K, 10 K, 300 K, and 300 K, respectively, using the NPT ensemble. For the production step of the MD simulations, the NPT ensemble, the Nose–Hoover chain thermostat, and the Martyna–Tobias–Klein barostat [33,34] were used. The coordinates were saved at intervals of 25 ps, ending with 8000 frames, and the MD simulations were sampled over 200 ns. The active site water structure and thermodynamic stability were examined for the protein–ligand complexes using SZMAP [35,36,37]. Positive (unfavorable) and negative (favorable) free energy hydration sites of the protein–ligand complexes were identified by implementing the semi-continuous solvation model of SZMAP to map the surface of the protein-binding pocket.

## 3. Results and Discussion

### 3.1. Comparison of Acacetin and Acacetin 7-O-Methyl Ether with Known Flavonoids

**Figure 1:**
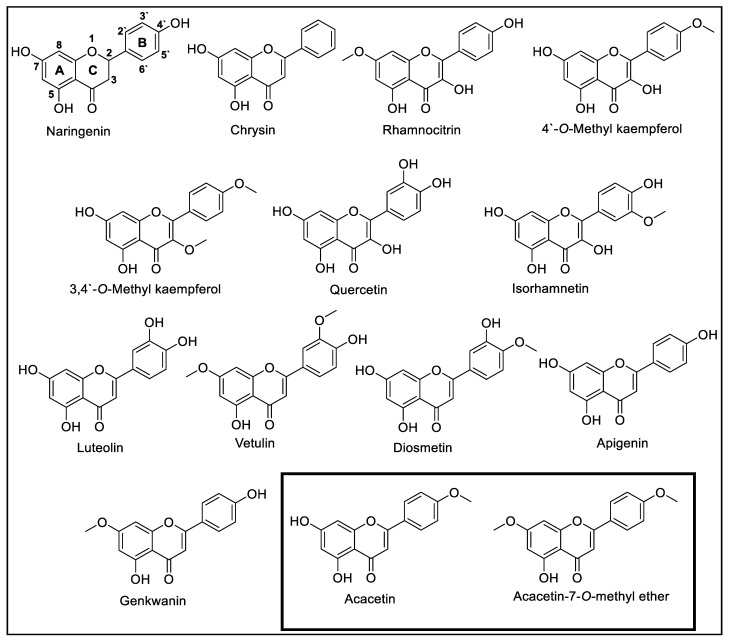
Comparison of acacetin and acacetin 7-*O*-methyl ether with known flavonoids.

The structural requirements of the flavonoid skeleton are provided in Figure 1 and a simple comparison of the IC_50_ values of the related flavonoids (Table 1) gave more insight into our computational approach.

The lack of a double bond between the C-2 and C-3 positions resulted in the depletion of MAO inhibition as seen in the flavanone naringenin [38]. The absence of substituents on ring B showed a slight preference for MAO-A in a manner similar to the flavone chrysin [39]. Monosubstitution on ring B of the flavonol scaffold decreased the SI of MAO-A versus MAO-B, improving the activity towards MAO-A in a manner comparable to the flavonols rhamnocitrin [40], 4′-*O*-methyl kaempferol, and 3, 4′-*O*-methyl kaempferol [41], while disubstitution of ring B resulted in the loss of MAO-A activity as seen with the flavonols quercetin [39] and isorhamnetin [42]. Dioxygenation at positions C-3′ and C-4′ on ring B resulted in the loss of selectivity towards MAO-B in a manner similar to the flavone luteolin [43], while methoxylation of the same positions gained MAO-B selectivity in a manner similar to vetulin [11] and diosmetin [42], respectively. Monosubstitution of ring B on the flavone scaffold at C-4′ resulted in no selectivity towards any MAO as seen with apigenin [44], genkwanin [40], and acacetin [9]. Methylation of the C-7 position increased selectivity towards MAO-B in a manner similar to acacetin-7-*O*-methyl ether [11]. Based on this SAR analysis, oxygenation at the C-5 and C-7 positions of ring A is significant for MAO inhibition. Oxygenation of the C-3 position resulted in MAO-A selectivity, while substitution on C-4′ resulted in MAO-B selectivity.

### 3.2. In Silico Optimization and Design

#### 3.2.1. Molecular Dynamics (MD) Simulations of the Binding Modes of Acacetin and Acacetin 7-*O*-Methyl Ether

Acacetin 7-*O*-methyl ether was recently discovered to be a selective MAO-B inhibitor with 0.198 µM inhibition of MAO-B versus MAO-A (>100 µM) [11]. The binding characteristics of acacetin 7-*O*-methyl ether were studied to understand the basis of target selectivity. Molecular constraints were imposed on the system during docking simulations based on our previous investigations [9]. The chemistry of the acacetin 7-*O*-methyl ether was mapped with the binding site environment using molecular dynamics (MD) simulations and thermodynamic calculations to better define the most abundant binding mode. The same analysis was carried out for acacetin (Figures S1–S73, see Supplementary Materials).

**Figure 2:**
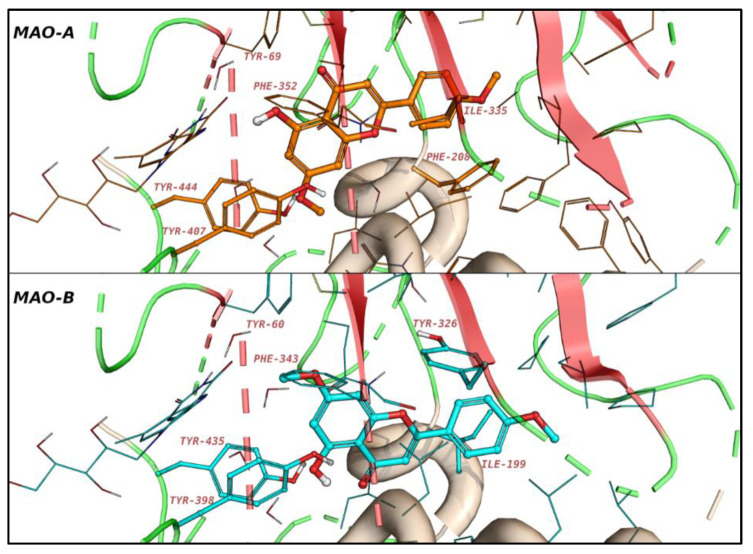
The predicted binding modes of acacetin ether in MAO-A and MAO-B. The gating amino acids (Ile335 and Phe208 of MAO-A, Ile199 and Tyr326 of MAO-B) and the aromatic cages (Tyr407 and Tyr444 of MAO-A, Tyr398 and Ty435 of MAO-B) are shown as sticks.

Acacetin 7-*O*-methyl ether demonstrated a very strong binding interaction with MAO-B and a very weak binding to MAO-A. Figure 2 shows the binding of acacetin 7-*O*-methyl ether with and without constraints to MAO-A and -B. Acacetin 7-*O*-methyl ether was docked preferentially with a docking score of −8.9 kcal/mol into MAO-B and a docking score of −6.7 kcal/mol into MAO-A, without forcing the constraints. The benzopyranone was located between the aromatic cages (Tyr residues) in both cases, and the terminal phenyl group interacted with the aromatic gating amino acids (Phe208 for MAO-A, and Tyr326 for MAO-B). Keeping the water molecules close to the gating amino acids clashed with the phenyl group in the case of MAO-A, while it allowed two possible poses for MAO-B.

**Figure 3:**
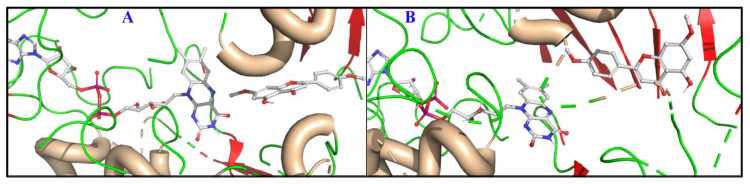
The two binding modes of acacetin 7-*O*-methyl ether. (**A**) Pose 1 with benzopyranone facing the FAD. (**B**) Pose 2 with the methoxyphenyl group facing the FAD. The protein is shown as a cartoon (helices are colored wheat, strands are colored red, and loops are colored green) and the ligands as sticks.

The two possible binding modes for acacetin 7-*O*-methyl ether in the case of MAO-B were visualized and are shown in Figure 3. The first pose of acacetin 7-*O*-methyl ether has benzopyranone facing the cofactor (referred to as pose 1, Figure 3A) and in the second pose, the methoxyphenyl group is facing the cofactor (referred to as pose 2, Figure 3B). Pose 1 has a better docking score compared with pose 2. The average root mean square deviation (RMSD) of pose 1 was 3.2 Å with respect to 1.6 Å for the protein backbone over the course of the MD simulations.

**Figure 4:**
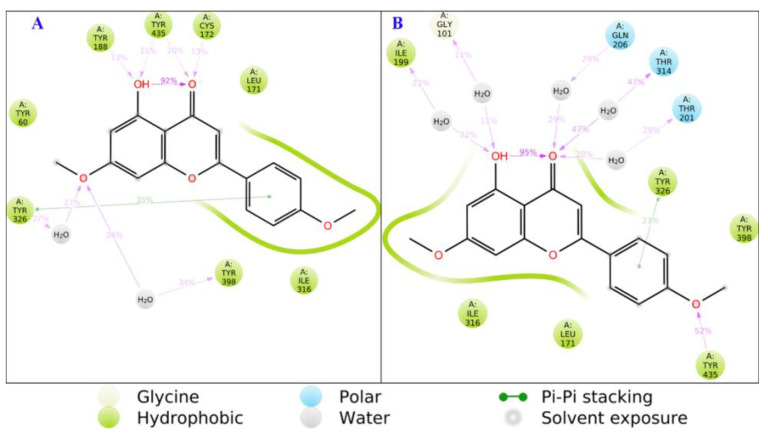
The 2D protein–ligand contacts for pose 1 (**A**) and pose 2 (**B**) for acacetin 7-*O*-methyl ether.

Pose 2 exhibited a RMSD value of ~1.6 Å, while the RMSD of the protein backbone was ~2.3 Å. Large local changes were observed along the protein chains between amino acids 99–102, particularly in the case of pose 2 (Figure S74). These local changes could be attributed to ligand binding (Figure 4 and Figure S75) leading to high fluctuations in the RMSD values (Figure S74).

#### 3.2.2. Active Site Hydration of MAO-B: MD Simulations, Thermodynamics, and Ligand Designs

There are 10 crystallographic water molecules in the binding pocket of MAO-B, which would have significant effects on ligand recognition and ligand design. The exact location of active site water molecules was confirmed by thermodynamic calculations to assess their contributions in ligand binding and target selectivity. Analysis of the MD simulations revealed that an average of three water molecules were fluctuating near the cofactor. In the case of pose 1, the ligand interacts with the hydrogen bond to Pro 102 through a water molecule for ~5% of the MD simulation time. On the other hand, pose 2 showed hydrogen bonds through water molecules to Phe 99 (less than 5%), Gly 101 (11%), and Pro 102 (~5%). More hydrophobic contacts were observed for pose 1, while more hydrogen bonds and water bridges were traced for pose 2 (Figure S75).

**Figure 5:**
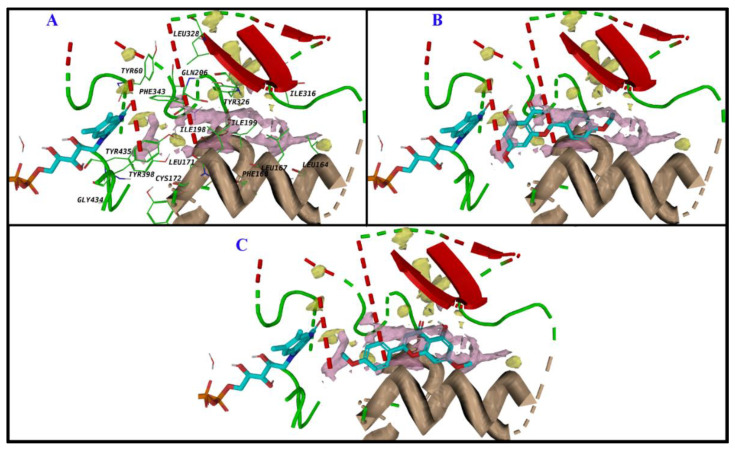
Water map of the MAO-B active site (**A**) and fitting of pose 1 (**B**) and pose 2 (**C**) with site hydration properties. Favorable regions for water molecules and polar groups are shown as yellow surfaces; hydrophobic regions are shown in purple.

**Figure 6:**
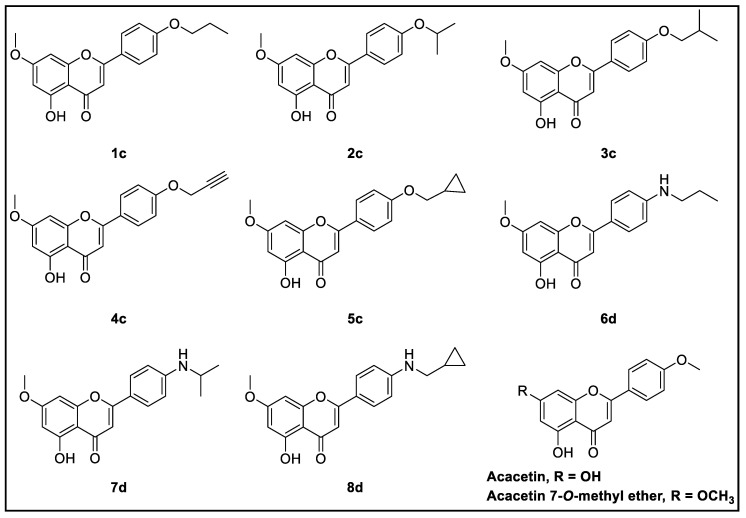
Designed acacetin 7-*O*-methyl ether analogs.

In conclusion, water molecules are more involved in the binding of pose 2 (Figure 4, Figure 5 and Figure 6). Several other important protein–ligand contacts for both poses were sampled over the course of MD simulations, including hydrophobic contacts, π-π stacking, hydrogen bonding, and interactions through water bridges with Leu 171, Leu 316, Tyr 326, Tyr 398, and Tyr 435 (Figure 4).

The active site water molecules (Figure 5A) were also investigated by water mapping calculations to compute their free energy and predict their effect on target selectivity. The binding pocket of MAO-B has a hydration shell near the cofactor (which is similar to what was found during the MD) that overlaps well with the hydroxyl group of the benzopyranone of pose 1 (Figure 5B). Therefore, to design high-affinity ligands, we should keep the hydroxyl group or modify the structure to have more polarity at the same position. A hydrophobic region (a positive free energy region) was noticed at the opposite site of FAD; and it covers the methoxyphenyl group of acacetin 7-*O*-methyl ether. To improve ligand binding, the polarity should be decreased at this position. The ligand chemistry of pose 2 did not fit well with the active site hydration map, while pose 1 showed a perfect match (Figure 5C).

Based on the interaction profile of acacetin 7-*O*-methyl ether as well as the thermodynamic properties of MAO-B’s active site, eight analogs were virtually designed to have small ether and/or amine modifications at C-4′ of the acacetin 7-*O*-methyl ether scaffold (Figure 6) and docked into the active sites of MAO-A and -B using Glide (Table S1).

The designed compounds were evaluated for their absorption, distribution, metabolism, and excretion (ADME) properties. They did not show any violation of the rules of five or three; they have a good balance of lipophilicity/hydrophilicity, and are expected to be orally bioavailable (Table S2). The compounds mapped well with the hydrophobic regions of the binding pocket while their polar chemistry fits with the small hydrophilic regions. Consequently, syntheses of five small ether derivatives were planned: propyl-ether (**1c**), isopropyl-ether (**2c**), isobutyl-ether (**3c**), propargylic-ether (**4c**), and methyl (cyclopropyl)-ether (**5c**). The role of the bioisosteric change of nitrogen from oxygen was also scrutinized. Thus, propyl amine (**6d**), isopropyl amine (**7d**), and methyl (cyclopropyl)-amine (**8d**) were synthesized.

### 3.3. Chemistry

**Scheme 1:**
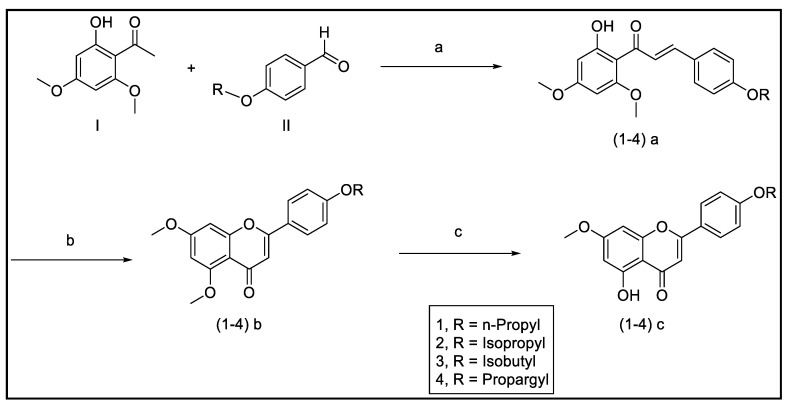
Reagents and conditions: (a) NaOH 50%, EtOH, rt, 16–20 h; (b) I_2_, DMSO, 140 °C, 4–6 h; (c) 1N BBr_3_, CH_2_Cl_2_, rt, 1–3 h.

**Scheme 2:**
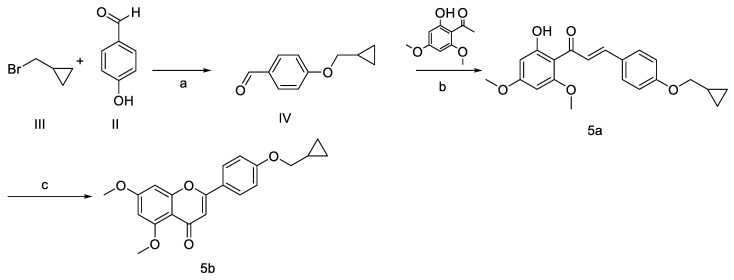
Reagents and conditions: (a) K_2_CO_3_, acetone, reflux; (b) NaOH 50%, EtOH, rt, 16–20 h; (c) I_2_, DMSO, 140 °C, 4–6 h.

**Scheme 3:**
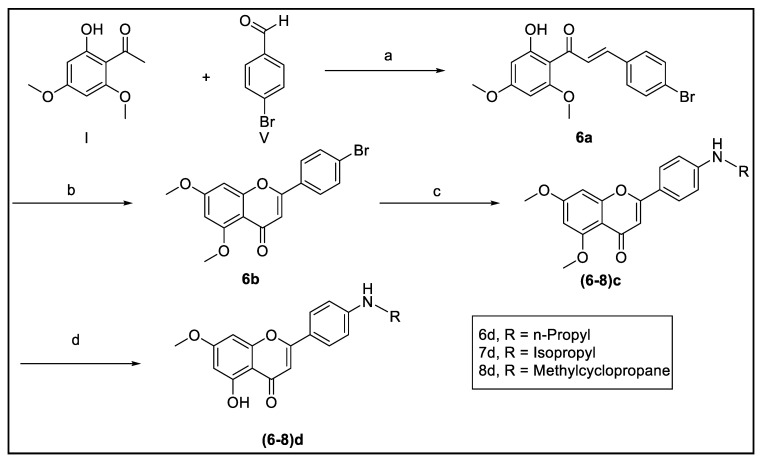
Reagents and conditions: (a) NaOH 50%, EtOH, rt, 16–20 h; (b) I_2_, DMSO, 140 °C, 4–6 h; (c) NaO*t*Bu, Pd_2_(dba)_3_, corresponding amines (propylamine, isopropylamine, and (aminomethyl)cyclopropane), BINAP, toluene, 80 °C, 48 h; (d) 1N BBr_3_, CH_2_Cl_2_, rt, 1–2 h.

The synthetic routes to access the target compounds (**1**–**5**) **c** and (**6**–**b**) **d** were based on reported synthetic procedures [45] (Scheme 1, Scheme 2 and Scheme 3).

#### 3.3.1. Synthesis of Modified Flavonoids **1**–**5**

Chalcones (**1**–**4**) **a** were afforded through Schmidt condensation by reacting 2′-hydroxy-4′,6′-dimethoxyacetophenone I and the corresponding 4-benzaldehydes II with 50% sodium hydroxide in ethanol. Treatment of the chalcones (**1**–**4**) with iodine crystals in a minimal amount of DMSO underwent cyclization to yield the dimethoxylated flavonoids (**1**–**4**) **b**. To obtain the target flavonoids (**1**–**4**) **c**, selective demethylation of position C-5 of (**1**–**4**) **b** was accomplished using boron tribromide (BBr_3_) in CH_2_Cl_2_ (Scheme 1).

Compound **5** was prepared as seen in Scheme 2. 4—(cyclopropylmethoxy) benzaldehyde IV was synthesized by reacting (bromomethyl) cyclopropane III with 4-hydroxybenzaldehyde II in acetone and potassium carbonate. Chalcone **5a** was prepared through Schmidt condensation by treatment of the cyclopropyl aldehyde IV with 2′-hydroxy-4′,6′-dimethoxyacetophenone I and 50% NaOH in ethanol. The intermediate flavonoid **5b** was obtained from the treatment of the chalcone with iodine crystals in a minimal amount of DMSO by undergoing cyclization. Several attempts to demethylate position C-5 were unsuccessful, thereby preventing the demethylation at C-5 and releasing the alkyl group to form the hydroxyl group at C-4′.

#### 3.3.2. Synthesis of Modified Flavonoids **6**–**8**

Compounds **6**–**8** were prepared as shown in Scheme 3 with an extra step involved in the formation of the brominated flavonoid at C-4′. Thus, the brominated chalcone **6a** was produced by treating 2′-hydroxy-4′,6′-dimethoxyacetophenone I with 4-bromobenzaldehyde V through Schmidt condensation with 50% sodium hydroxide in ethanol. The brominated flavonoid **6b** was prepared from the treatment of the chalcone with iodine crystals in a minimal amount of DMSO by cyclization. The flavonoids (**6**–**8**) **c** were prepared by treating compound **6b** with sodium *tert*-butoxide, tris(dibenzylideneacetone) dipalladium, 1,1′-binaphthalene-2,2′-diylbis(diphenylphosphine), and the corresponding amines such as propylamine, isopropylamine, and (aminomethyl)cyclopropane in toluene. Finally, the selective demethylated flavonoids at position 5 (**6**–**8**) **d** were prepared with boron tribromide in dichloromethane (Scheme 3). With the exception of compound **5c**, the evaluation of MAO-A and -B inhibition was performed on the final and intermediate compounds.

### 3.4. Biological Assays

#### 3.4.1. Determination of Inhibitory Effects of Modified Flavonoids and Intermediates on MAO-A and -B

The synthesized acacetin 7-*O*-methyl ether analogs and their intermediates were evaluated against MAO-A and -B inhibition assays. The IC_50_ values of acacetin 7-*O*-methyl ether analogs were prominent against MAO-B compared with MAO-A. Flavonoid **3c** showed > 3000-fold selective inhibition for MAO-B over MAO-A (Table 2). The significant selectivity obtained for the oxygenated analogs clearly validated our design. Bioisosteric replacement of oxygen with nitrogen at position C-4′ diminished the selectivity of MAO-B considerably (see Table 2). The presence of the hydroxyl group at C-5 showed increased selectivity towards MAO-B. Thus, the activity and selectivity are increased for the series as follows: hydroxyls at C-5 (**1c**–**4c**) > protected-methylated flavonoids at C-5 (**1b**–**6b**) > chalcones (**1a**–**6a**) > amine flavonoids at C-4′ (**6c,d**–**8c,d**). Compounds (**1**–**4**) **c** exhibited more than a thousand-fold SI (Table 2), equal to or more potent than safinamide (Table 2), and were considered for further studies to understand their mechanisms of inhibition. Figure S76 shows the inhibition dose–response for the potent analogs (**1**–**4**) **c**.

#### 3.4.2. Evaluation of MAO-B Inhibition Kinetics and Analysis of Binding and Time-Dependent Inhibition of Modified Flavonoids (**1**–**4**) **c**

Modified flavonoids (**1**–**4**) **c** were evaluated against MAO-B at varying concentrations of kynuramine, a nonselective substrate, to investigate the nature of inhibition of the enzymes. Based on dose–concentration inhibition, three to five concentrations (below and above the concentration of IC_50_ values) were selected for the inhibition kinetics experiment. Modified flavonoids (**1**–**4**) **c** inhibited the enzymatic activity of MAO-B with considerably high affinity *Ki* at nanomolar units with a range from 43 to 68 nM (Table 3).

**Figure 7:**
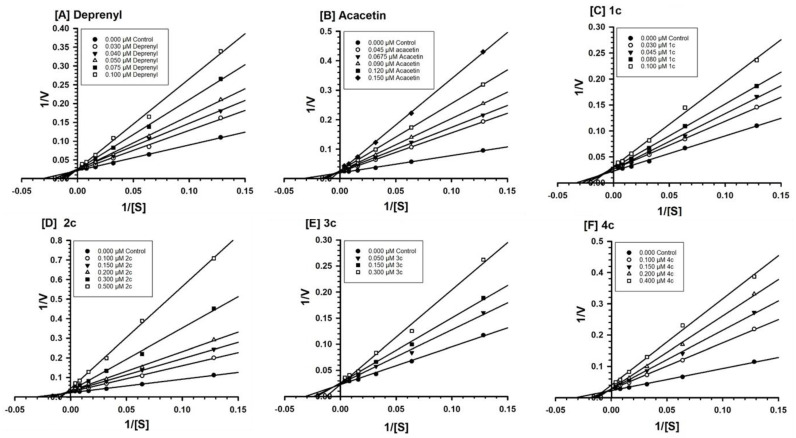
Kinetic characteristics of inhibition of recombinant human MAO-B with [**A**] deprenyl [**B**] acacetin, [**C**] **1c**, [**D**] **2c**, [**E**] **3c**, and [**F**] **4c**; V = nmoles/min/mg protein and S = substrate kynuramine concentration (μM).

Binding of modified flavonoids (**1**–**4**) **c** with human MAO-B affected *K_m_* (i.e., the affinity of the substrate for the enzyme) as well as *V_max_* (maximum enzyme activity) values, indicating the type of MAO-B inhibition by the analogs: flavonoid **1c** (mixed/partially reversible), **2c** (mixed/partially reversible), **3c** (mixed/irreversible), and **4c** (mixed/irreversible) (Table 3, Figure 7).

**Figure 8:**
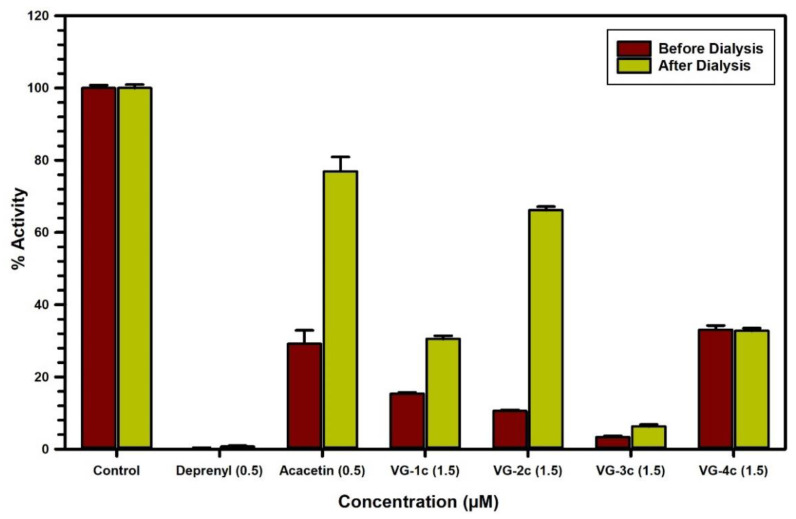
Analysis of the nature of binding of deprenyl (0.5 μM), acacetin (0.5 μM), **1c** (1.5 μM), **2c** (1.5 μM), **3c** (1.5 μM), and **4c** (1.5 μM) with recombinant human MAO-B by recovery of catalytic activity of the enzyme after equilibrium dialysis dissociation. Each bar shows the mean ± S.D. of triplicate values.

The binding characteristics of the modified flavonoids (**1**–**4**) **c** with MAO-B were examined by equilibrium dialysis to measure the dissociation of the enzyme–inhibitor complex (Figure 8). MAO-B enzyme was incubated with the highest concentration of the analogs for 20 min at 37 °C to allow for binding of the inhibitor with the enzyme and formation of the enzyme–inhibitor complex. Then, the mixtures of the enzyme–inhibitor complex were dialyzed overnight at 4 °C using 25 mM KHPO_4_ (pH 7.4) buffer. The enzyme activities were examined before and after dialysis. Incubation of MAO-B with 1.5 µM of modified flavonoids (**1**–**4**) **c** caused more than 70% inhibition of activity and only the enzyme activities of **1c** (30%) and **2c** (66%) were recovered after dialysis. Thus, the binding of **1c** was partially reversible, while **2c** was reversible with MAO-B (Figure 8, Table 3).

To inspect the time-dependent binding inhibition of MAO-B, the enzyme was pre-incubated with the analog for 0 to 15 min at concentrations that caused nearly 40–80% inhibition depending on the analog (Figure S77). For compounds **1c**, **3c**, and **4c**, 70–80% inhibition was seen at the concentrations given in Figure S77. Meanwhile, compound **2c** showed 30–50% of MAO-B enzyme inhibition. The control enzyme without inhibitor was also run concurrently. For validation, the MAO- B standards were run simultaneously for the time-dependent assay.

### 3.5. Computational Analysis of Enzyme–Inhibitor Interactions for Modified Flavonoids (**1**–**4**) **c**

**Figure 9:**
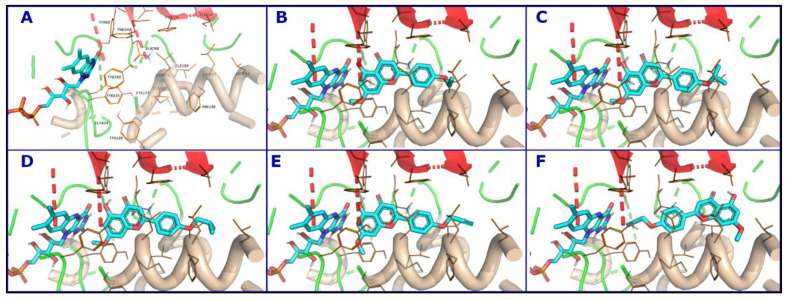
Binding modes of acacetin 7-*O*-methyl ether derivatives and surrounding amino acids. The amino acids that interact with the ligand are shown as lines (**A**), Compounds **1c** (**B**), **2c** (**C**), **3c** (**D**), and **4c** (**E**,**F**). There are two possible orientations for **4c** in the binding site.

Based on the remarkable experimental selectivity of MAO-B towards the designed compounds (**1**–**4**) **c**, further computational analysis was conducted. These analogs did not show good docking poses in MAO-A due to the clashes between the R group and the amino acids in the substrate binding site. The size and nature of the R group are essential for selective targeting. In general, these four acacetin 7-*O*-methyl ether analogs exploited the hydrophobic nature of the amino acids in the binding pocket of MAO-B, particularly Tyr 398 and Tyr 435, near the FAD cofactor by forming π-π stacking with their aromatic functionalities. The non-polar nature of the R group matches the hydrophobic nature of the Leu and Ile amino acids on the other side of the binding site. Considerable hydrophobic contacts were observed between the ligands and Leu 167, Leu 171, Ile 199, Tyr 236, and Phe 343. The ligands placed properly their polar chemistry to form strong hydrogen bonds with the backbones of Leu 171, Ile 199, and the side chain of Gln 206 (Figure 9). There is room in the binding pocket for water molecules to bridge the interactions between the ligands and amino acids, principally near the FAD.

## 4. Conclusions

Based on our understanding of the SAR of the flavonoid skeleton and the molecular modeling studies, eight flavonoid analogs were designed. All the analogs did not demonstrate acceptable docking poses in the active site of MAO-A but they had good binding scores in MAO-B (Table S1). They also fitted well with the thermodynamic properties of the MAO-B active site. The flavonoid compounds with *O*-propyl, *O*-isopropyl, *O*-isobutyl, and *O*-propargyl substituents showed excellent selectivity towards MAO-B, in the range of a 1200- to 3200-fold difference from MAO-A.

Rasagiline and selegiline are FDA-approved irreversible MAO-B inhibitors that exhibited a selectivity of 103- and 127-fold against MAO-A, while safinamide is a reversible MAO-B inhibitor with a selectivity of 1000–1500-fold towards MAO-B [8,9,46]. The synthesized flavonoids *O*-propyl and *O*-isopropyl are reversible MAO-B inhibitors with a selectivity of 1478- and 1921-fold against MAO-A, respectively. *O*-isobutyl was the most selective of all the synthesized compounds with 3225-fold selectivity towards MAO-B, while *O*-propargyl exhibited 1279-fold selectivity. *O*-isobutyl and *O*-propargyl were both irreversible MAO-B inhibitors. *O*-propyl, *O*-isopropyl, *O*-isobutyl, and *O*-propargyl substituents are potential candidates worth pursuing. Based on the results of these derivatives, (**1**–**4**) **c** with *O*-alkyl (a short chain with three to four carbon atoms) substituents at position C-4′ yield high selectivity toward MAO-B. The nitrogen amine series did not display any significant selectivity towards MAO-B. This could be attributed to the high electronegativity of oxygen compared with nitrogen. The study of the longer alkyl chain on C-4′ should be explored. It is also worth pursuing the introduction of halogens at position 7, which might help improve the ADMET properties. Introduction of an electronegative atom such as nitrogen at position 8 would be interesting to understand the SAR of ring A in MAO activity. Another chemical modification that would help us to understand the activity of MAO-B versus MAO-A in the flavonoid scaffold that is worth exploring is the substitution of the B ring, introducing *O*-substituent groups at position C-3′ or C-4′ as on vetulin or diosmetin (see Table 1), which both show a slight preference towards MAO-B. Flavonoids have limited clinical use due to their significant challenges related to the pharmacokinetic and pharmacodynamic profiles. They have poor oral absorption along with extensive hepatic metabolism and low solubility, thereby resulting in poor bioavailability [12]. Modifying the chemical structure by substituting the flavonoid rings can help overcome the solubility issue. For example, the C-7 position on the A ring can be replaced with fluorine to improve its solubility and bioavailability.

## Figures and Tables

**Table 1:** Comparison of the IC_50_ values of acacetin and acacetin 7-O-methyl ether with known flavonoids.

Flavonoids	MAO-A (IC_50_, μM) *	MAO-B (IC_50_, μM) *	Selectivity Index MAO-A/B
Naringenin [38]	955 ± 129	288 ± 18	3.32
Chrysin [39]	0.25 ± 0.04	1.04 ± 0.17	0.24
Rhamnocitrin [40]	0.051 ± 0.001	2.97 ± 2.97	0.02
4′-*O*-Methyl kaempferol [38]	1.350 ± 0.198	>100	0.01
3,4′-Di-*O*-methyl kaempferol [41]	0.033 ± 0.042	9.667 ± 2.39	0.03
Quercetin [39]	1.52 ± 0.09	28.39 ± 5.41	0.05
Isorhamnetin [42]	6.42 ± 7.69	21.2 ± 4.99	0.30
Luteolin [43]	8.57 ± 0.47	>100	0.08
Vetulin [11]	18.79 ± 0.29	0.44 ± 0.01	42.70
Diosmetin [42]	5.74 ± 0.57	1.58 ± 0.88	3.63
Apigenin [44]	0.64 ± 0.11	1.12 ± 0.27	0.30
Genkwanin [40]	0.14 ± 0.01	0.35 ± 0.03	0.40
Acacetin [9]	0.121 ± 0.001	0.049 ± 0.0007	4.28
Acacetin 7-*O*-methyl ether [11]	>100	0.198 ± 0.001	>505.05

* The IC_50_ values computed from the dose–response inhibition curves are mean ± S.D. of triplicate observations. Luteolin data were obtained by chemiluminescent assays using a MAO-Glo kit. All other flavonoid IC_50_ data were obtained by spectrometric/fluorometric techniques similar to those followed here using kynuramine as a substrate and human recombinant MAO A/B (see Section 2.4), except for naringenin where a chromogenic solution was used and MAO A/B where a rat liver mitochondrial fraction was used.

**Table 2:** Inhibition (IC_50_ values) of recombinant human monoamine oxidases A and B by acacetin 7-*O*-methyl ether analogs and intermediates.

Synthesized Analogs	MAO-A (IC_50_, μM) *	MAO-B (IC_50_, μM) *	Selectivity Index MAO-A/B
**1a**	0.515 ± 0.024	0.40 ± 0.024	1.3
**2a**	0.117 ± 0.05	0.049 ± 0.0035	2.4
**3a**	0.42 ± 0.002	0.22 ± 0.0211	1.9
**4a**	9.42 ± 1.78	8.33 ± 0.7871	1.1
**5a**	1.30 ± 0.09	0.90 ± 0.035	1.4
**1b**	>100	1.70 ± 0.076	>58.8
**2b**	>100	2.64 ± 0.0880	>37.9
**3b**	>100	7.23 ± 2.1438	>13.8
**4b**	>100	>100	>1
**4a**	9.42 ± 1.78	8.33 ± 0.7871	1.1
**4b**	>100	>100	>1
**5b**	>100	22.82 ± 2.098	>4.4
**1c**	48.78 ± 2.01	0.033 ± 0.0042	1478.2
**2c**	30.74 ± 0.023	0.016 ± 0.0070	1921.3
**3c**	>100	0.031 ± 0.0070	>3225.8
**4c**	62.70 ± 5.21	0.049 ± 0.0014	1279.6
**6a**	12.349 ± 0.249	>100	>0.1
**6b**	87.830 ± 5.449	28.407 ± 2.639	3.1
**6c**	>100	22.097 ± 2.479	>4.5
**7c**	37.492 ± 0.476	9.447 ± 0.113	4.0
**8c**	39.095 ± 5.144	12.727 ± 0.290	3.1
**6d**	85.484 ± 1.585	1.554 ± 0.137	55.0
**7d**	40.500 ± 2.374	0.417 ± 0.012	97.1
**8d**	39.114 ± 0.555	2.185 ± 0.088	17.9
Acacetin	0.105 ± 0.0014	0.042 ± 0.0021	2.5
Acacetin 7-O-methyl ether [11]	>100	0.198 ± 0.001	>505.05
*Clorgyline*	0.0039 ± 0.0002	2.15 ± 0.212	0.002
*Deprenyl*	33.00 ± 1.411	0.046 ± 0.0014	717.4
*Safinamide* [46]	90.00 ± 2.470	0.060 ± 0.005	1500.0
*Harmine*	0.0031 ± 0.0003	39.000 ± 1.412	0.00008

* The IC_50_ values computed from the dose–response inhibition curves are the mean ± S.D. of triplicate observations.

**Table 3:** Inhibition/binding affinity constant (*Ki*) values for inhibition of recombinant human MAO-B by modified flavonoids (**1**–**4**) **c**, acacetin, acacetin 7-*O*-methyl ether, and deprenyl.

Compound	Monoamine Oxidase A		Monoamine Oxidase B	
	Ki (nM) *	Type of Inhibition	Ki (nM) *	Type of Inhibition
**1c**	-	-	43 ± 3.8	mixed/partially reversible
**2c**	-	-	52 ± 3.1	mixed/reversible
**3c**	-	-	37 ± 9.5	mixed/irreversible
**4c**	-	-	68 ± 7.1	mixed/irreversible
Acacetin	30 ± 1.8	competitive/reversible	21 ± 1.8	competitive/reversible
Acacetin 7-*O*-methyl ether [11]	--		45 ± 3.0	competitive/partially reversible
Deprenyl	-	-	43 ± 4.0	mixed/irreversible

* Values are the mean ± S.D. of triplicate experiments.

## Data Availability

All supporting results are reported in the manuscript.

## Supplementary Materials

The following are available online at https://www.mdpi.com/article/10.3390/biomedicines9101304/s1, Figures S1–S77, and Tables S1 and S2. Additional data, such as ^1^H-NMR spectra, ^13^C-NMR spectra, HRMS spectra, and HPLC analysis of compounds **1**–**8**, computational interactions of acacetin and acacetin 7-*O*-methyl ether and MAO-A and MAO-B, and docking scores and ADME predictions for acacetin 7-*O*-methyl ether analogs (PDF).

## References

[1] Scott L., Dawson V.L., Dawson T.M. (2017). Trumping Neurodegeneration: Targeting Common Pathways Regulated by Autosomal Recessive Parkinson’s Disease Genes. Exp. Neurol..

[2] Pringsheim T., Jette N., Frolkis A., Steeves T.D.L. (2014). The Prevalence of Parkinson’s Disease: A Systematic Review and Meta-Analysis. Mov. Disord..

[3] Blesa J., Trigo-Damas I., Quiroga-Varela A., Jackson-Lewis V.R. (2015). Oxidative Stress and Parkinson’s Disease. Front. Neuroanat..

[4] Sarrafchi A., Bahmani M., Shirzad H., Rafieian-Kopaei M. (2016). Oxidative Stress and Parkinson’s Disease: New Hopes in Treatment with Herbal Antioxidants. Curr. Pharm. Des..

[5] Tong J., Rathitharan G., Meyer J.H., Furukawa Y., Ang L.-C., Boileau I., Guttman M., Hornykiewicz O., Kish S.J. (2017). Brain Monoamine Oxidase B and A in Human Parkinsonian Dopamine Deficiency Disorders. Brain.

[6] Szökő É., Tábi T., Riederer P., Vécsei L., Magyar K. (2018). Pharmacological Aspects of the Neuroprotective Effects of Irreversible MAO-B Inhibitors, Selegiline and Rasagiline, in Parkinson’s Disease. J. Neural Transm..

[7] Liguori C., Stefani A., Mercuri N.B., Pierantozzi M. (2018). Effective Treatment of Restless Legs Syndrome by Safinamide in Parkinson’s Disease Patients. Sleep Med..

[8] Guglielmi P., Carradori S., Ammazzalorso A., Secci D. (2019). Novel Approaches to the Discovery of Selective Human Monoamine Oxidase-B Inhibitors: Is There Room for Improvement?. Expert Opin. Drug Discov..

[9] Chaurasiya N.D., Gogineni V., Elokely K.M., León F., Núñez M.J., Klein M.L., Walker L.A., Cutler S.J., Tekwani B.L. (2016). Isolation of Acacetin from *Calea urticifolia* with Inhibitory Properties against Human Monoamine Oxidase-A and -B. J. Nat. Prod..

[10] Semwal R.B., Semwal D.K., Combrinck S., Trill J., Gibbons S., Viljoen A. (2019). Acacetin–A Simple Flavone Exhibiting Diverse Pharmacological Activities. Phytochem. Lett..

[11] Chaurasiya N.D., Zhao J., Pandey P., Doerksen R.J., Muhammad I., Tekwani B.L. (2019). Selective Inhibition of Human Monoamine Oxidase B by Acacetin 7-Methyl Ether Isolated from *Turnera diffusa* (Damiana). Molecules.

[12] Amawi H., Ashby C.R., Tiwari A.K. (2017). Cancer Chemoprevention Through Dietary Flavonoids: What’s Limiting?. Chin. J. Cancer.

[13] Jin C.-F., Wang Z.-Z., Chen K.-Z., Xu T.-F., Hao G.-F. (2020). Computational Fragment-Based Design Facilitates Discovery of Potent and Selective Monoamine Oxidase-B (MAO-B) Inhibitor. J. Med. Chem..

[14] Aldrich C., Bertozzi C., Georg G.I., Kiessling L., Lindsley C., Liotta D., Merz K.M., Schepartz A., Wang S. (2017). The Ecstasy and Agony of Assay Interference Compounds. J. Med. Chem..

[15] Baell J.B., Holloway G.A. (2010). New Substructure Filters for Removal of Pan Assay Interference Compounds (PAINS) from Screening Libraries and for Their Exclusion in Bioassays. J. Med. Chem..

[16] Sterling T., Irwin J.J. (2015). ZINC 15–Ligand Discovery for Everyone. J. Chem. Inf. Model..

[17] Chaurasiya N.D., León F., Ding Y., Gómez-Betancur I., Benjumea D., Walker L.A., Cutler S.J., Tekwani B.L. (2017). Interactions of Desmethoxyyangonin, a Secondary Metabolite from *Renealmia alpinia*, with Human Monoamine Oxidase-A and Oxidase-B. Evid. Based Complement. Altern. Med..

[18] Parikh S., Hanscom S., Gagne P., Crespi C., Patten C. (2002). A Fluorescent-Based, High-Throughput Assay for Detecting Inhibitors of Human Monoamine Oxidase A and B. BD Biosci. Discov. Labware.

[19] (2017). Small-Molecule Drug Discovery Suite 2017-4.

[20] Greenwood J.R., Calkins D., Sullivan A.P., Shelley J.C. (2010). Towards the Comprehensive, Rapid, and Accurate Prediction of the Favorable Tautomeric States of Drug-Like Molecules in Aqueous Solution. J. Comput. Aided Mol. Des..

[21] Shelley J.C., Cholleti A., Frye L.L., Greenwood J.R., Timlin M.R., Uchimaya M. (2007). Epik: A Software Program for *pK*_a_ Prediction and Protonation State Generation for Drug-Like Molecules. J. Comput. Aided Mol. Des..

[22] (2017). Schrödinger Release 2017-4: Epik.

[23] Harder E., Damm W., Maple J., Wu C., Reboul M., Xiang J.Y., Wang L., Lupyan D., Dahlgren M.K., Knight J.L. (2016). OPLS3: A Force Field Providing Broad Coverage of Drug-like Small Molecules and Proteins. J. Chem. Theory Comput..

[24] Friesner R.A., Murphy R.B., Repasky M.P., Frye L.L., Greenwood J.R., Halgren T.A., Sanschagrin P.C., Mainz D.T. (2006). Extra Precision Glide: Docking and Scoring Incorporating a Model of Hydrophobic Enclosure for Protein-Ligand Complexes. J. Med. Chem..

[25] Halgren T.A., Murphy R.B., Friesner R.A., Beard H.S., Frye L.L., Pollard W.T., Banks J.L. (2004). Glide: A New Approach for Rapid, Accurate Docking and Scoring. 2. Enrichment Factors in Database Screening. J. Med. Chem..

[26] Friesner R.A., Banks J.L., Murphy R.B., Halgren T.A., Klicic J.J., Mainz D.T., Repasky M.P., Knoll E.H., Shelley M., Perry J.K. (2004). Glide: A New Approach for Rapid, Accurate Docking and Scoring. 1. Method and Assessment of Docking Accuracy. J. Med. Chem..

[27] (2017). Schrödinger Release 2017-4: Glide.

[28] Arul Murugan N., Chiotis K., Rodriguez-Vieitez E., Lemoine L., Ågren H., Nordberg A. (2019). Cross-interaction of tau PET tracers with monoamine oxidase B: Evidence from in silico modelling and in vivo imagining. Eur. J. Nucl. Med. Mol. Imagining.

[29] Arul Murugan N., Zaleśny R. (2020). Multiscale modeling of two-photon probes for Parkinson’s diagnostics based on monoamine oxidase B biomarker. J. Chem. Inf. Model..

[30] Arul Murugan N., Muvva C., Jeyarajpandian C., Jeyakanthan J., Subramanian V. (2020). Performance of force-field and machine learning-based scoring functions in ranking MAO-B protein-inhibitor complexes in relevance to developing Parkinson’s therapeutics. Int. J. Mol. Sci..

[31] (2017). Schrödinger Release 2017-4: LigPrep.

[32] Elokely K.M., Doerksen R.J. (2013). Docking Challenge: Protein Sampling and Molecular Docking Performance. J. Chem. Inf. Model..

[33] Bowers K.J., Chow D.E., Xu H., Dror R.O., Eastwood M.P., Gregersen B.A., Klepeis J.L., Kolossvary I., Moraes M.A., Sacerdoti F.D. Scalable Algorithms for Molecular Dynamics Simulations on Commodity Clusters. Proceedings of the SC ’06: Proceedings of the 2006 ACM/IEEE Conference on Supercomputing.

[34] (2021). Desmond Molecular Dynamics System, D.E. Shaw Research, New York, NY, 2021. Maestro-Desmond Interoperability Tools, Schrödinger, New York, NY. https://www.schrodinger.com/citations.

[35] SZMAP 1.2.1.4.

[36] Bayden A.S., Moustakas D.T., Joseph-McCarthy D., Lamb M.L. (2015). Evaluating Free Energies of Binding and Conservation of Crystallographic Waters Using SZMAP. J. Chem. Inf. Model..

[37] Elokely K., Velisetty P., Delemotte L., Palovcak E., Klein M.L., Rohacs T., Carnevale V. (2016). Understanding TRPV1 Activation by Ligands: Insights from the Binding Modes of Capsaicin and Resiniferatoxin. Proc. Natl. Acad. Sci. USA..

[38] Olsen H.T., Stafford G.I., van Staden J., Christensen S.B., Jäger A.K. (2008). Isolation of the MAO-Inhibitor Naringenin from *Mentha aquatica* L. J. Ethnopharmacol..

[39] Larit F., Elokely K.M., Chaurasiya N.D., Benyahia S., Nael M.A., León F., Abu-Darwish M.S., Efferth T., Wang Y.-H., Belouahem-Abed D. (2018). Inhibition of Human Monoamine Oxidase A and B by Flavonoids Isolated from Two Algerian Medicinal Plants. Phytomedicine.

[40] Baek S.C., Park M.H., Ryu H.W., Lee J.P., Kang M.-G., Park D., Park C.M., Oh S.-R., Kim H. (2019). Rhamnocitrin Isolated from *Prunus padus* var. *seoulensis*: A Potent and Selective Reversible Inhibitor of Human Monoamine Oxidase A. Bioorg. Chem..

[41] Chaurasiya N.D., Midiwo J., Pandey P., Bwire R.N., Doerksen R.J., Muhammad I., Tekwani B.L. (2020). Selective Interactions of *O*-Methylated Flavonoid Natural Products with Human Monoamine Oxidase-A and -B. Molecules.

[42] Carradori S., Gidaro M.C., Petzer A., Costa G., Guglielmi P., Chimenti P., Alcaro S., Petzer J.P. (2016). Inhibition of Human Monoamine Oxidase: Biological and Molecular Modeling Studies on Selected Natural Flavonoids. J. Agric. Food Chem..

[43] Park S.E., Paudel P., Wagle A., Seong S.H., Kim H.R., Fauzi F.M., Jung H.A., Choi J.S. (2020). Luteolin, a Potent Human Monoamine Oxidase-A Inhibitor and Dopamine D_4_ and Vasopressin V_1A_ Receptor Antagonist. J. Agric. Food Chem..

[44] Chaurasiya N.D., Ibrahim M.A., Muhammad I., Walker L.A., Tekwani B.L. (2014). Monoamine Oxidase Inhibitory Constituents of Propolis: Kinetics and Mechanism of Inhibition of Recombinant Human MAO-A and MAO-B. Molecules.

[45] Chen H., Mrazek A.A., Wang X., Ding C., Ding Y., Porro L.J., Liu H., Chao C., Hellmich M.R., Zhou J. (2014). Design, Synthesis, and Characterization of Novel Apigenin Analogues that Suppress Pancreatic Stellate Cell Proliferation In vitro and Associated Pancreatic Fibrosis In vivo. Bioorg. Med. Chem..

[46] Pandey P., Chaurasiya N.D., Tekwani B.L., Doerksen R.J. (2018). Interactions of endocannabinoid virodhamine and related analogs with human monoamine oxidase-A and-B. Biochem. Pharmacol..

