# Supplementary material for: Computationally Assisted Lead Optimization of Novel Potent and Selective MAO-B Inhibitors
Source: Biomedicines. 2021 Sep 24;9(10):1304. doi: 10.3390/biomedicines9101304 (PMC8533211; doi:10.3390/biomedicines9101304)
Supplement: Supplementary file 1 [file biomedicines-09-01304-s001.zip › biomedicines-1354093-supplementary.pdf]

Supplementary Materials

Computationally Assisted Lead Optimization of Novel Potent and Selective MAO-B Inhibitors

Vedanjali Gogineni [1,2], Manal A. Nael [3,4,†], Narayan D. Chaurasiya [5,6,†], Khaled M. Elokely [3,4], Christopher R. McCurdy [1,7], John M. Rimoldi [1], Stephen J. Cutler [1,8], Babu L. Tekwani [5,6,*] and Francisco León [1,8,*]

[1] Department of BioMolecular Sciences, Division of Medicinal Chemistry, University of Mississippi, Oxford, MS 38677, USA; gogineni@musc.edu (V.G.); cmccurdy@cop.ufl.edu (C.R.M.); jrimoldi@olemiss.edu (J.M.R.); sjcutler@cop.sc.edu (S.J.C.)
[2] Affiliate Instructor, Drug Discovery and Biomedical Sciences, College of Pharmacy, Medical University of South Carolina, Charleston, SC 29425, USA
[3] Department of Chemistry and ICMS, Temple University, Philadelphia, PA 19122, USA; manal.nael@temple.edu (M.A.N.); kelokely@temple.edu (K.M.E.)
[4] Department of Pharmaceutical Chemistry, Faculty of Pharmacy, Tanta University, Tanta 31527, Egypt
[5] Division of Drug Discovery, Southern Research, Birmingham, AL 35205, USA; nchaurasiya@southernresearch.org
[6] National Center for Natural Products Research, Research Institute of Pharmaceutical Sciences, University of Mississippi, Oxford, MS 38677, USA
[7] Department of Medicinal Chemistry, College of Pharmacy, University of Florida, Gainesville, FL 32610, USA
[8] Department of Drug Discovery and Biomedical Sciences, College of Pharmacy, University of South Carolina, Columbia, SC 29208, USA
* Correspondence: btekwani@southernresearch.org (B.L.T.); jleon@mailbox.sc.edu (F.L.)
† These authors contributed equally to this study.

Table of Contents **Pages**

**Suppl. Figures S1-S3.**	**Spectra for compound 1a**	**S3-S4**
**Suppl. Figures S4-S6.**	**Spectra for compound 1b**	**S4-S5**
**Suppl. Figures S7-S10.**	**Spectra for compound 1c**	**S6-S7**
**Suppl. Figures S11-S13.**	**Spectra for compound 2a**	**S8-S9**
**Suppl. Figures S14-S16.**	**Spectra for compound 2b**	**S9-S10**
**Suppl. Figures S17-S20.**	**Spectra for compound 2c**	**S11-S12**
**Suppl. Figures S21-S23.**	**Spectra for compound 3a**	**S13-S14**
**Suppl. Figures S24-S26.**	**Spectra for compound 3b**	**S14-S15**
**Suppl. Figures S27-S30.**	**Spectra for compound 3c**	**S16-S17**
**Suppl. Figures S31-S33.**	**Spectra for compound 4a**	**S18-S19**
**Suppl. Figures S34-S36.**	**Spectra for compound 4b**	**S19-S20**
**Suppl. Figures S37-S40.**	**Spectra for compound 4c**	**S21-S22**
**Suppl. Figures S41-S43.**	**Spectra for compound 5a**	**S23-S24**
**Suppl. Figures S44-S46.**	**Spectra for compound 5b**	**S24-S25**
**Suppl. Figures S47-S49.**	**Spectra for compound 6a**	**S26-S27**
**Suppl. Figures S50-S52.**	**Spectra for compound 6b**	**S27-S28**
**Suppl. Figures S53-S55.**	**Spectra for compound 6c**	**S29-S30**
**Suppl. Figures S56-S59.**	**Spectra for compound 6d**	**S30-S32**
**Suppl. Figures S60-S62.**	**Spectra for compound 7c**	**S32-S33**
**Suppl. Figures S63-S66.**	**Spectra for compound 7d**	**S34-S35**
**Suppl. Figures S67-S70.**	**Spectra for compound 8d**	**S36-S37**
**Computational interactions of acacetin and MAO A and MAO B**		**S38-S340**
**Suppl. Figure S71. Protein-ligand RMSD**		**S38**
**Suppl. Figure S72. Interaction profile of acacetin in MAO-A**		**S39**
**Suppl. Figure S73. Interaction profile of acacetin in MAO-B**		**S40**
**Suppl Figure S74. Protein and ligand RMSD**		**S41**
**Suppl Figure S75. Protein-ligand contacts and their interaction fractions for pose 1 (A) and pose 2 (B) for acacetin 7-*O*-methyl ether**		**S41**
**Suppl Figure S76. Dose-response profile for in vitro inhibition of recombinant human MAO-B by deprenyl, acacetin, and potent acacetin 7-*O*-methyl ether analogs (1-4) c (% activity vs concentration)**		**S42**
**Suppl Figure S77. Time-dependent inhibition of recombinant human MAO-B by Deprenyl, acacetin, 1c, 2c, 3c and 4c**		**S42**
**Suppl Table S1. Docking scores of acacetin 7-*O*-methyl ether analogs**		**S43**
**Suppl. Table S2. ADME predictions for acacetin analogs**		**S44**

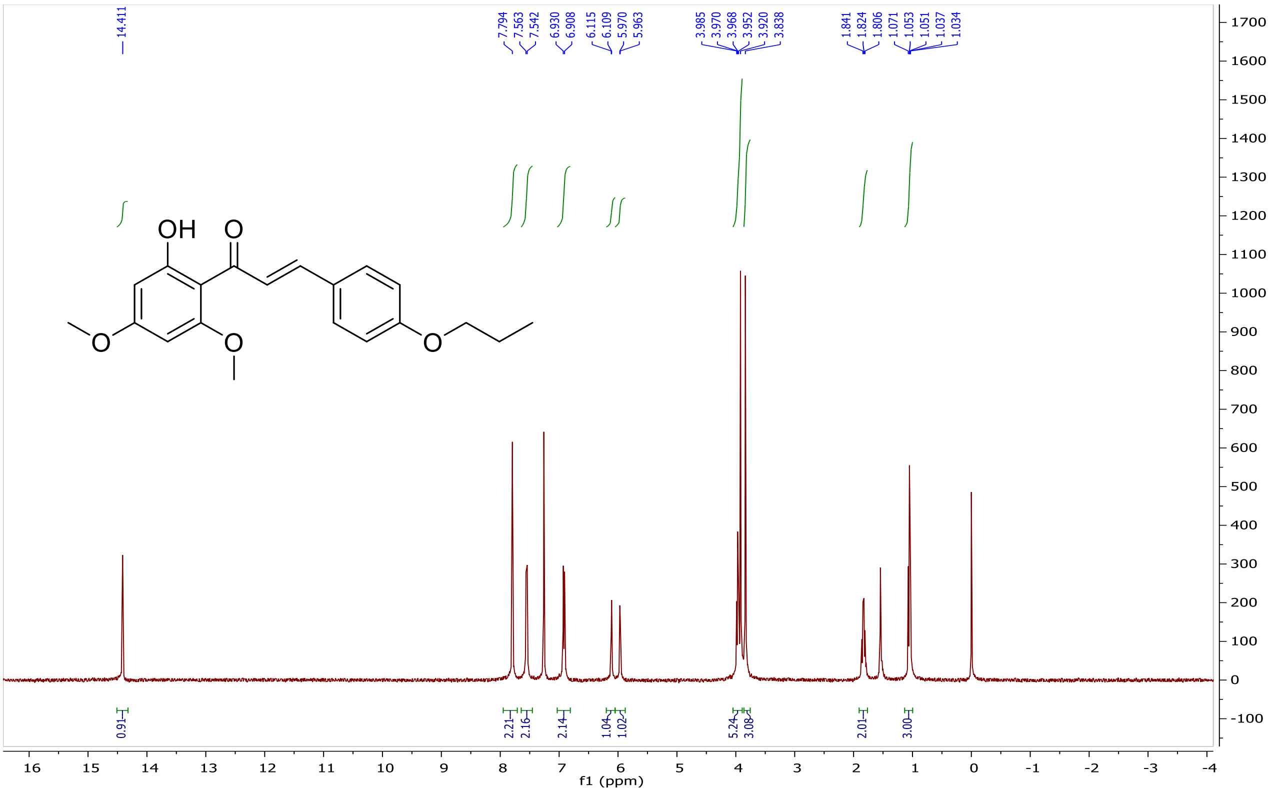

Figure S1. ^{1}**H NMR spectrum of chalcone (1a) in CDCl₃, 400 MHz**

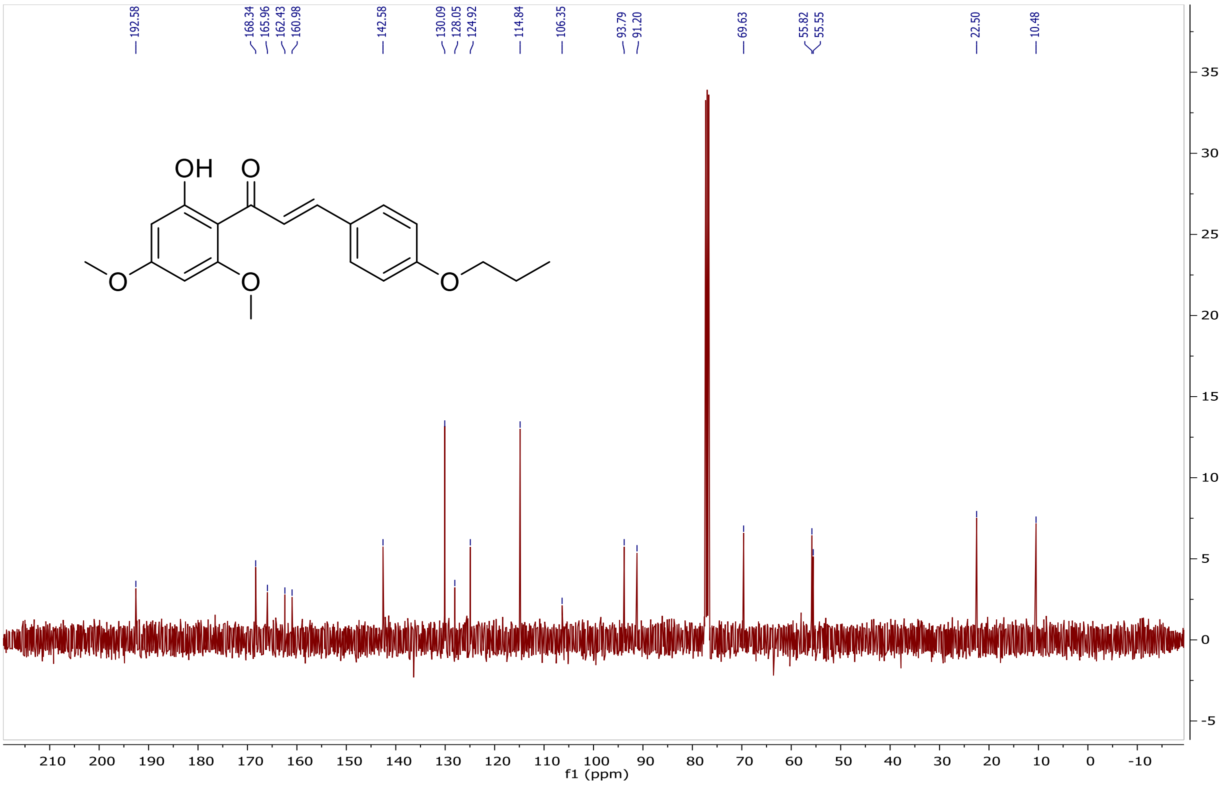

Figure S2. ^{13}**C NMR spectrum of chalcone (1a) in CDCl₃, 100 MHz**

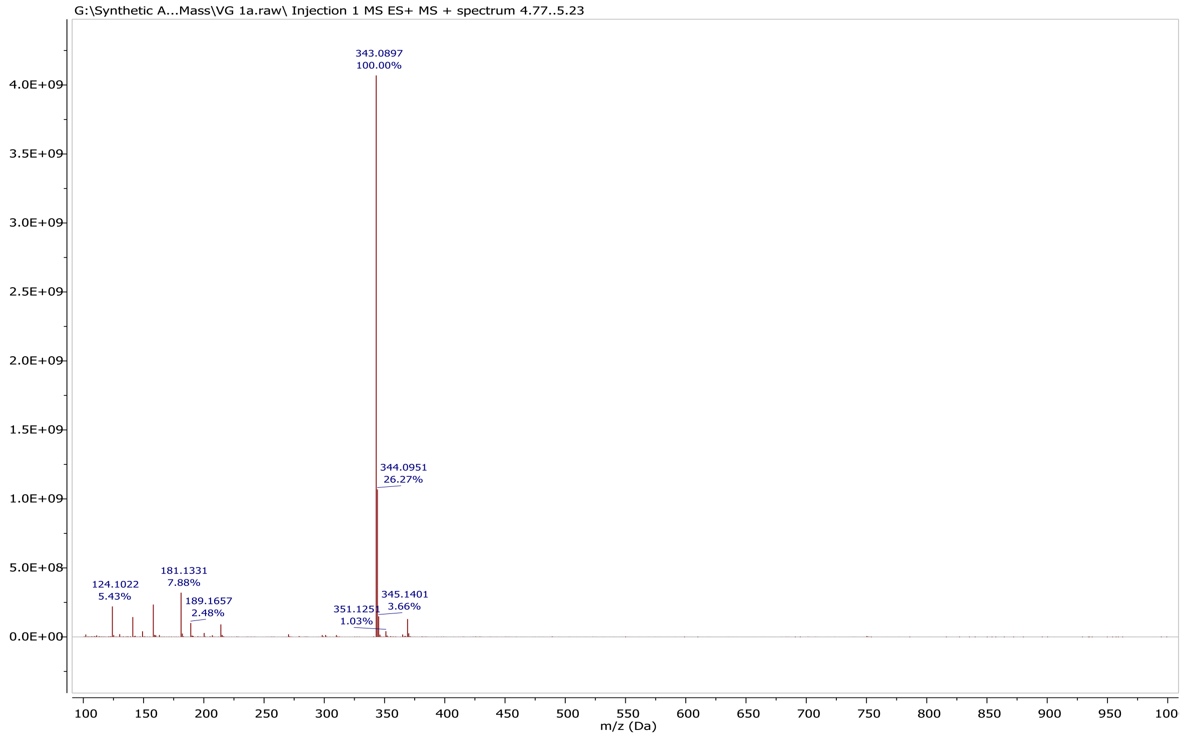

Figure S3. ESIMS spectrum of chalcone (1a).

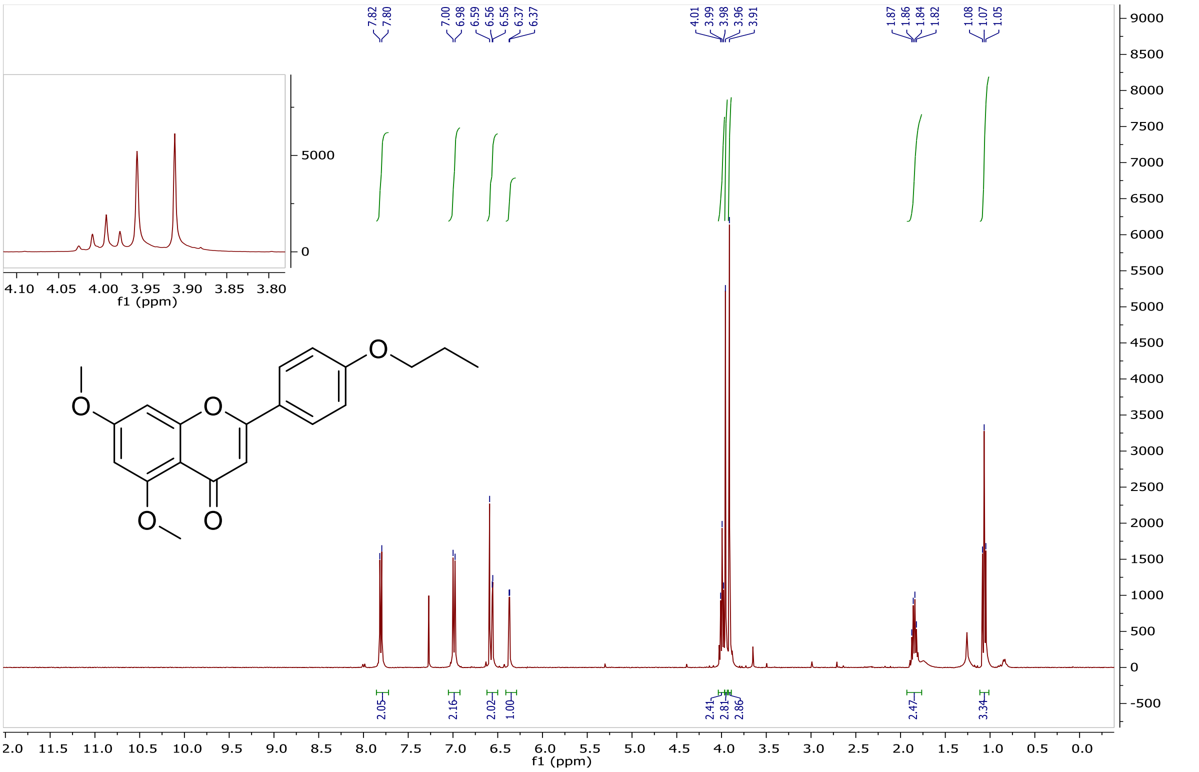

Figure S4. ^{1}H NMR spectrum of flavonoid (1b) in $CDCl_3$, 400 MHz

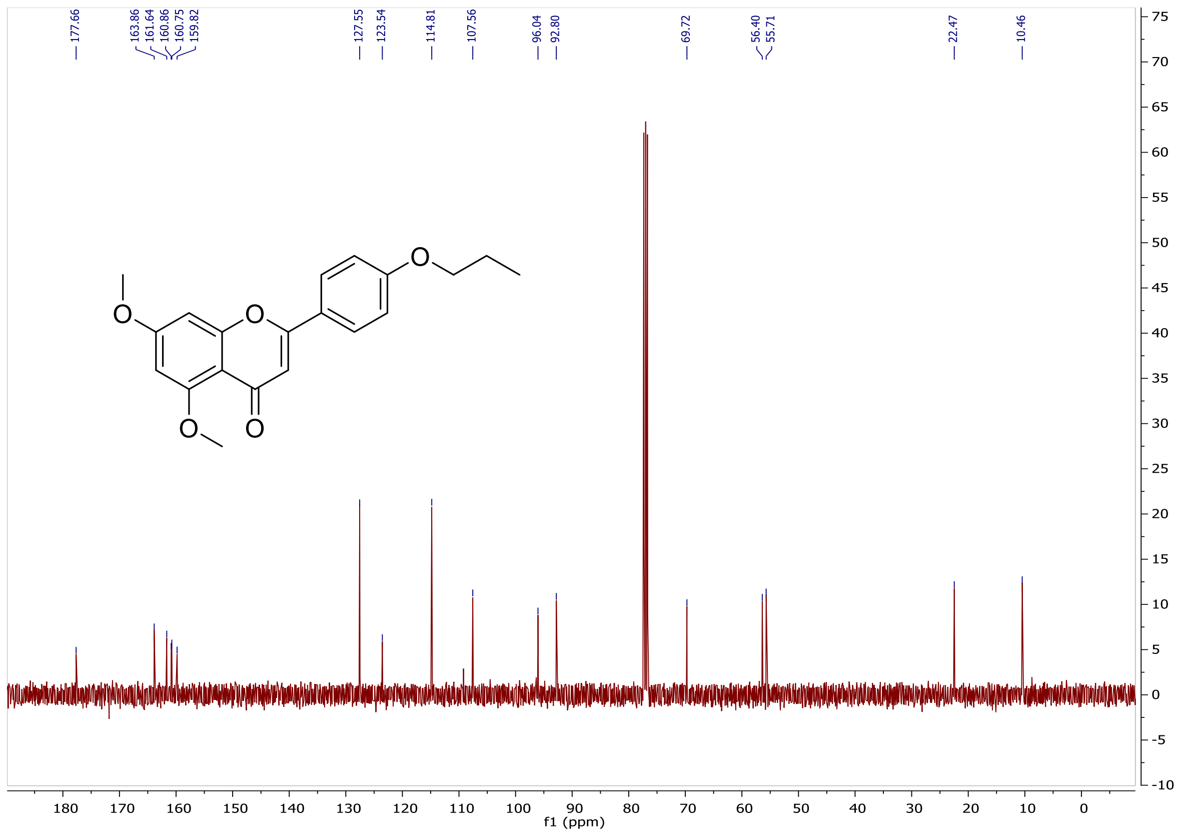

Figure S5. ^{13}C NMR spectrum of flavonoid (1b) in $CDCl_3$, 100 MHz

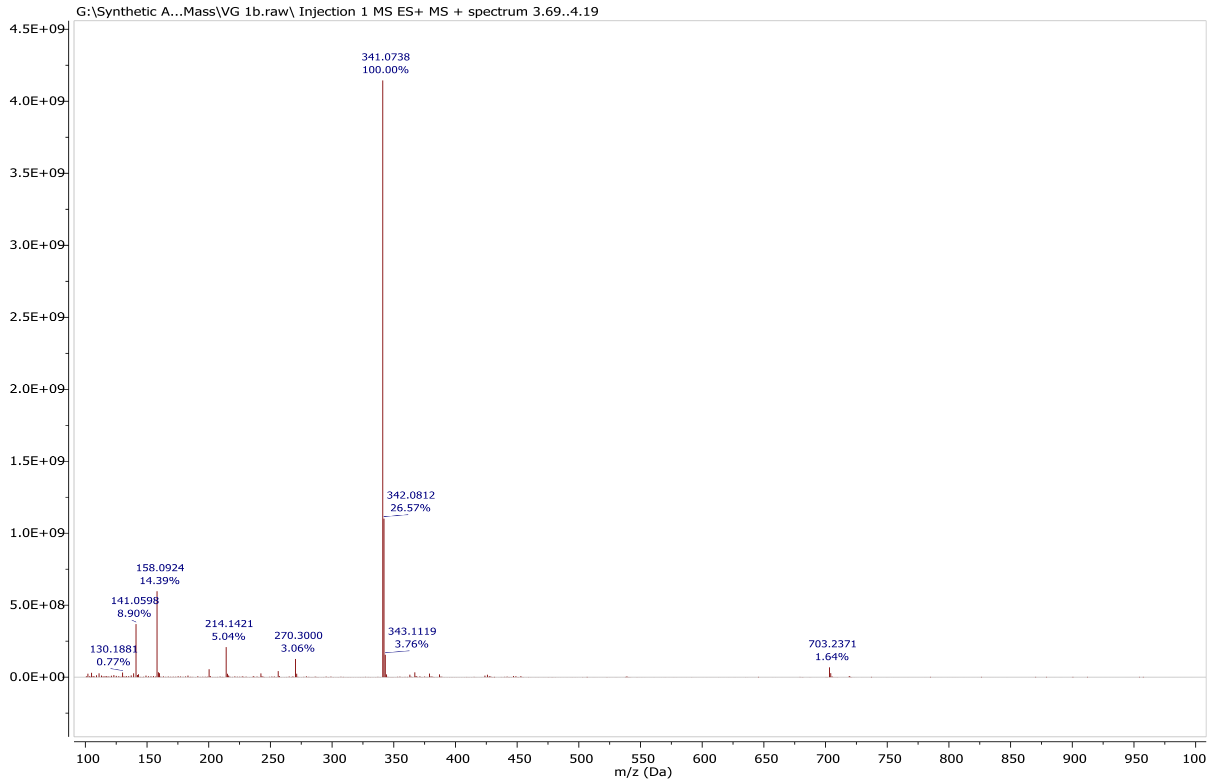

Figure S6. ESIMS spectrum of flavonoid (1b).

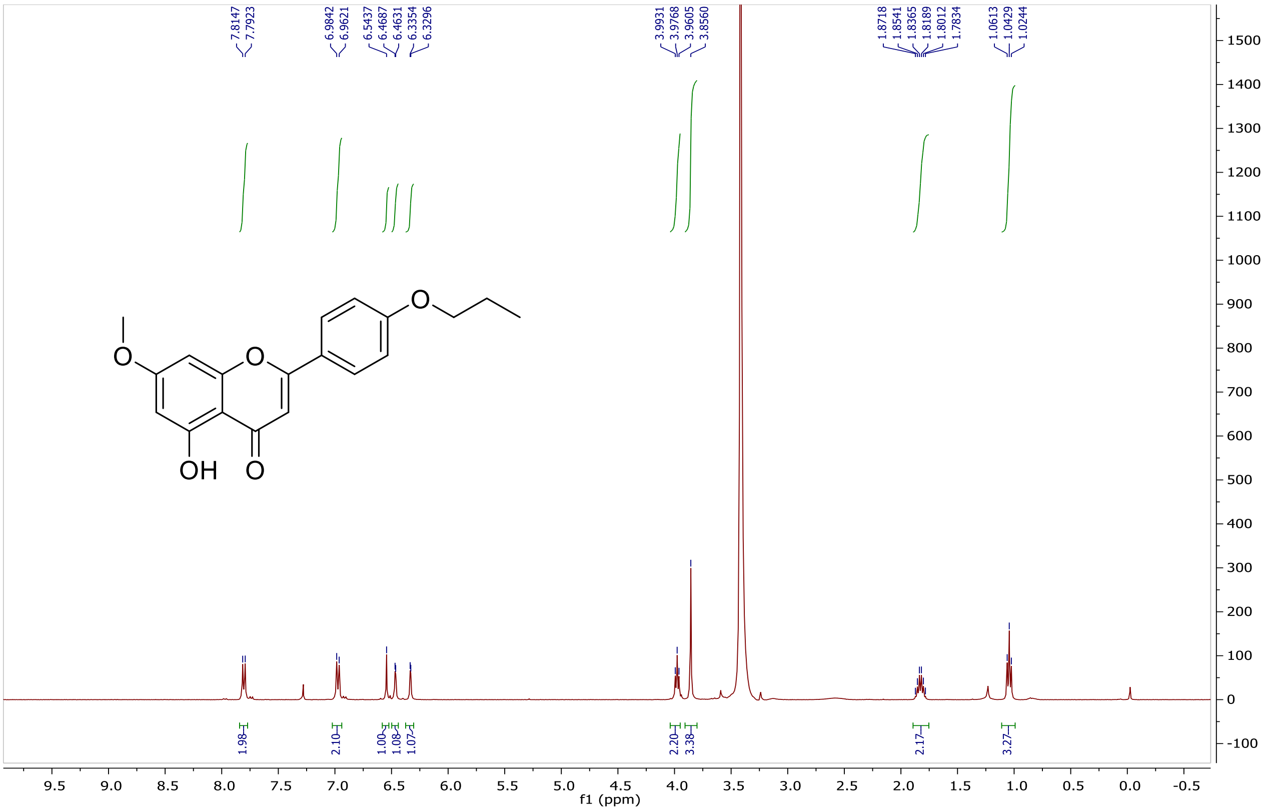

Figure S7. ^{1}H NMR spectrum of flavonoid (1c) in $CDCl_3$, 400 MHz

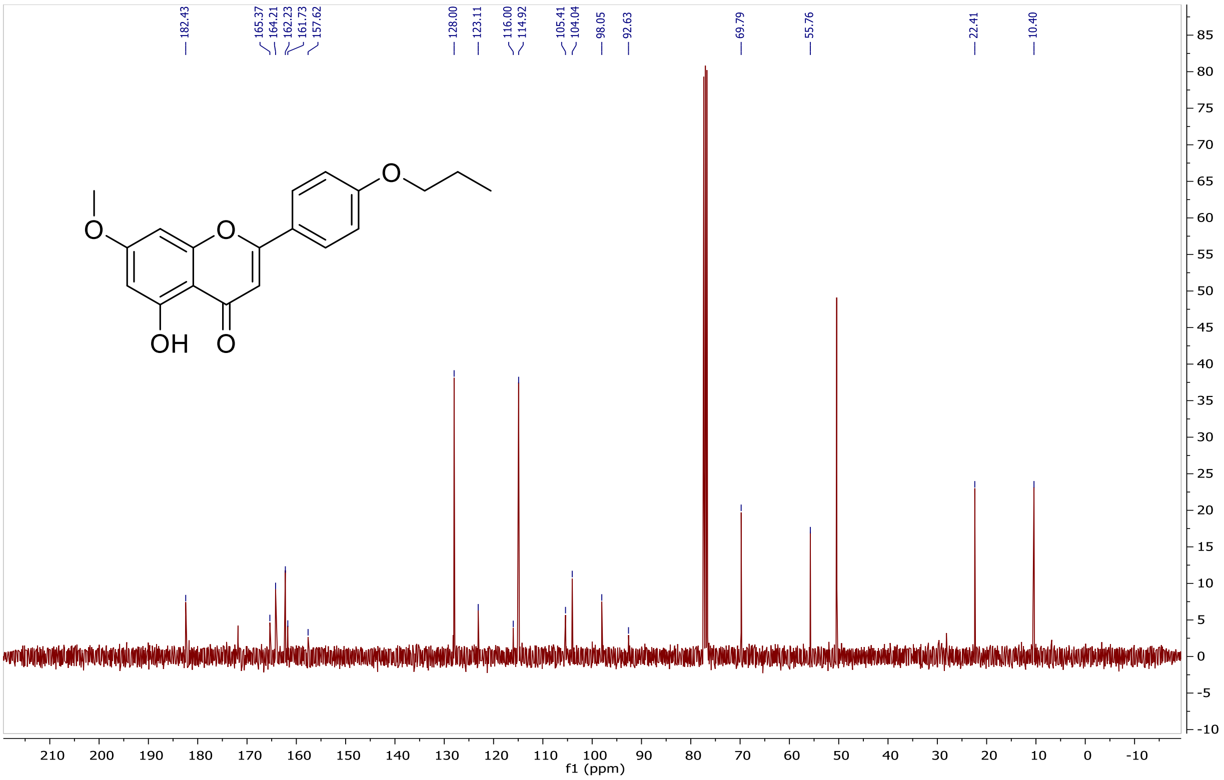

Figure S8. ^{13}C NMR spectrum of flavonoid (1c) in $CDCl_3$, 100 MHz

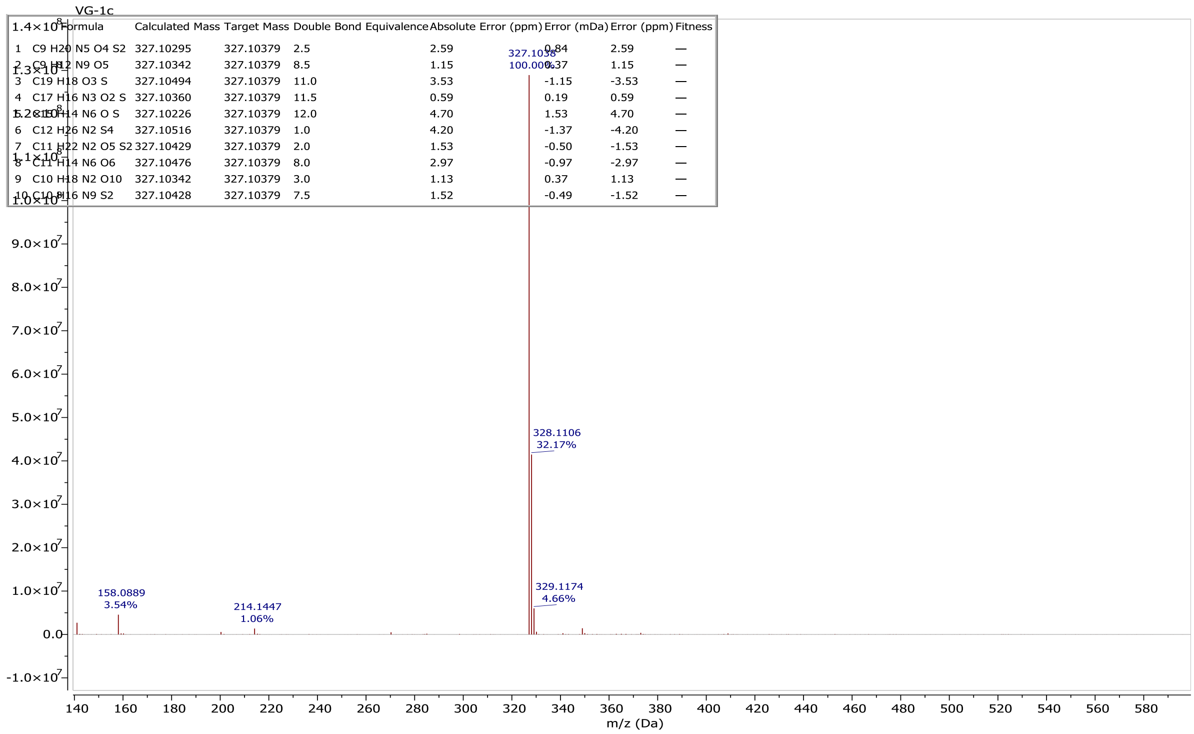

	Formula	Calculated Mass	Target Mass	Double Bond Equivalence	Absolute Error (ppm)	Error (mDa)	Error (ppm)	Fitness
1	C9 H20 N5 O4 S2	327.10295	327.10379	2.5	2.59	0.84	2.59	—
2	C9 H12 N9 O5	327.10342	327.10379	8.5	1.15	0.37	1.15	—
3	C19 H18 O3 S	327.10494	327.10379	11.0	3.53	-1.15	-3.53	—
4	C17 H16 N3 O2 S	327.10360	327.10379	11.5	0.59	0.19	0.59	—
5	C15 H14 N6 O S	327.10226	327.10379	12.0	4.70	1.53	4.70	—
6	C12 H26 N2 S4	327.10516	327.10379	1.0	4.20	-1.37	-4.20	—
7	C11 H22 N2 O5 S2	327.10429	327.10379	2.0	1.53	-0.50	-1.53	—
8	C11 H14 N6 O6	327.10476	327.10379	8.0	2.97	-0.97	-2.97	—
9	C10 H18 N2 O10	327.10342	327.10379	3.0	1.13	0.37	1.13	—
10	C10 H16 N9 S2	327.10428	327.10379	7.5	1.52	-0.49	-1.52	—

Figure S9. ESIMS of flavonoid (1c).

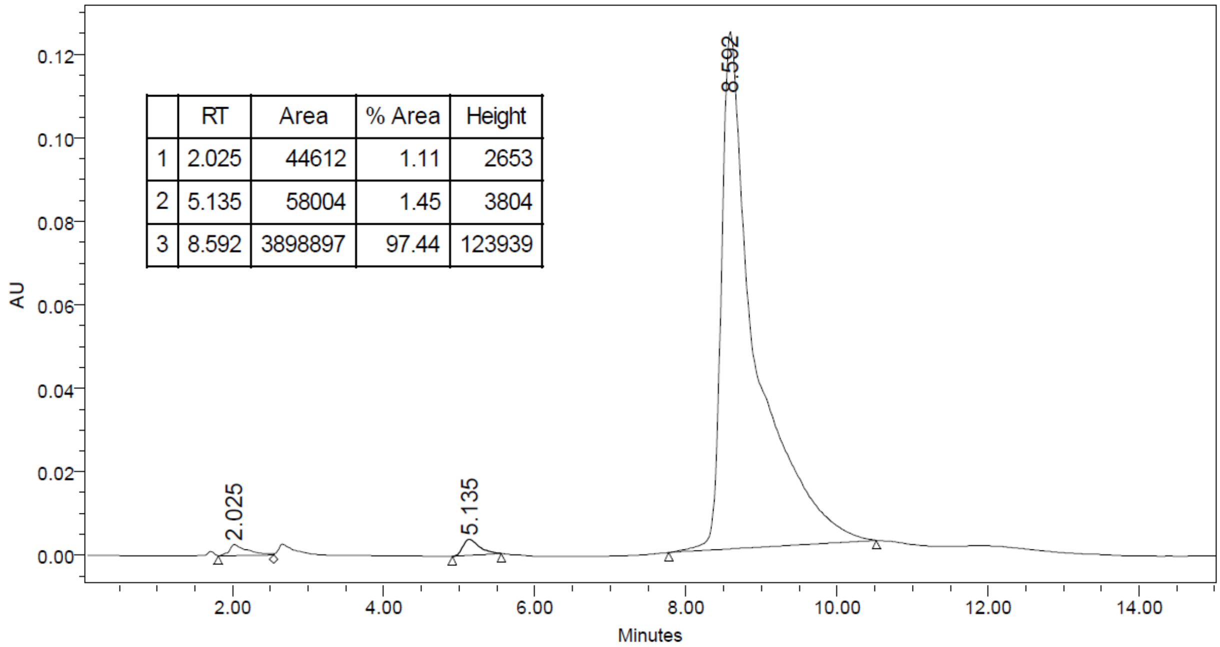

	RT	Area	% Area	Height
1	2.025	44612	1.11	2653
2	5.135	58004	1.45	3804
3	8.592	3898897	97.44	123939

Figure S10. HPLC analysis of flavonoid (1c).

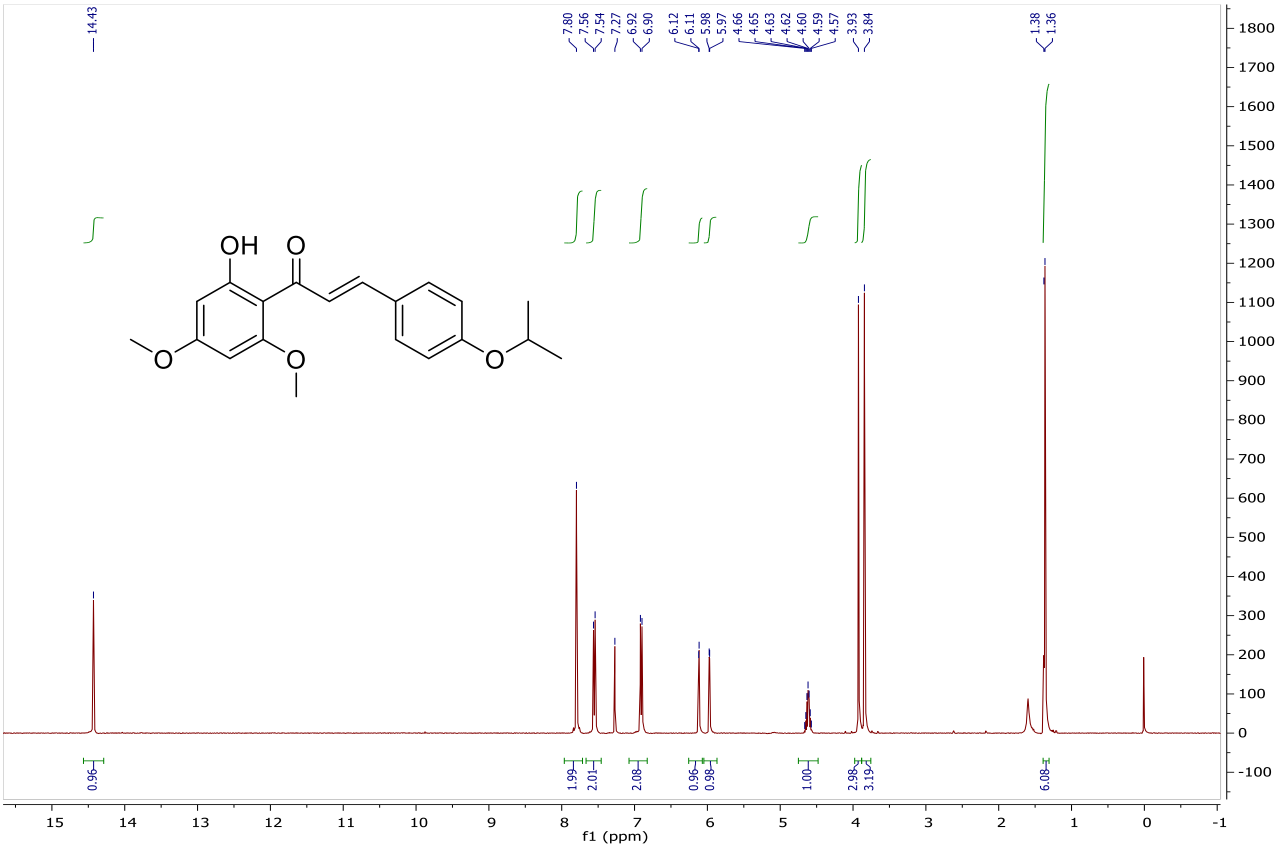

Figure S11. ^{1}H NMR spectrum of chalcone (2a) in $CDCl_3$, 400 MHz

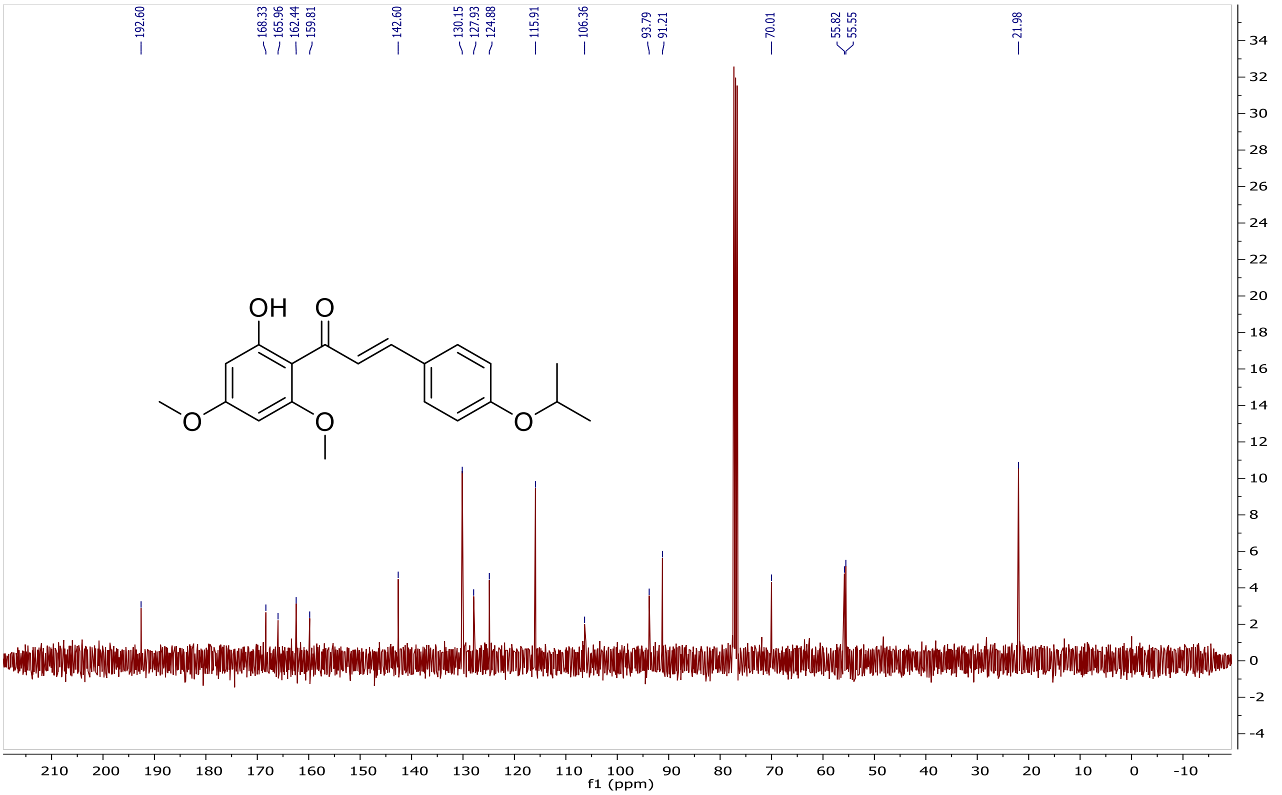

Figure S12. ^{13}C NMR spectrum of chalcone (2a) in $CDCl_3$, 100 MHz

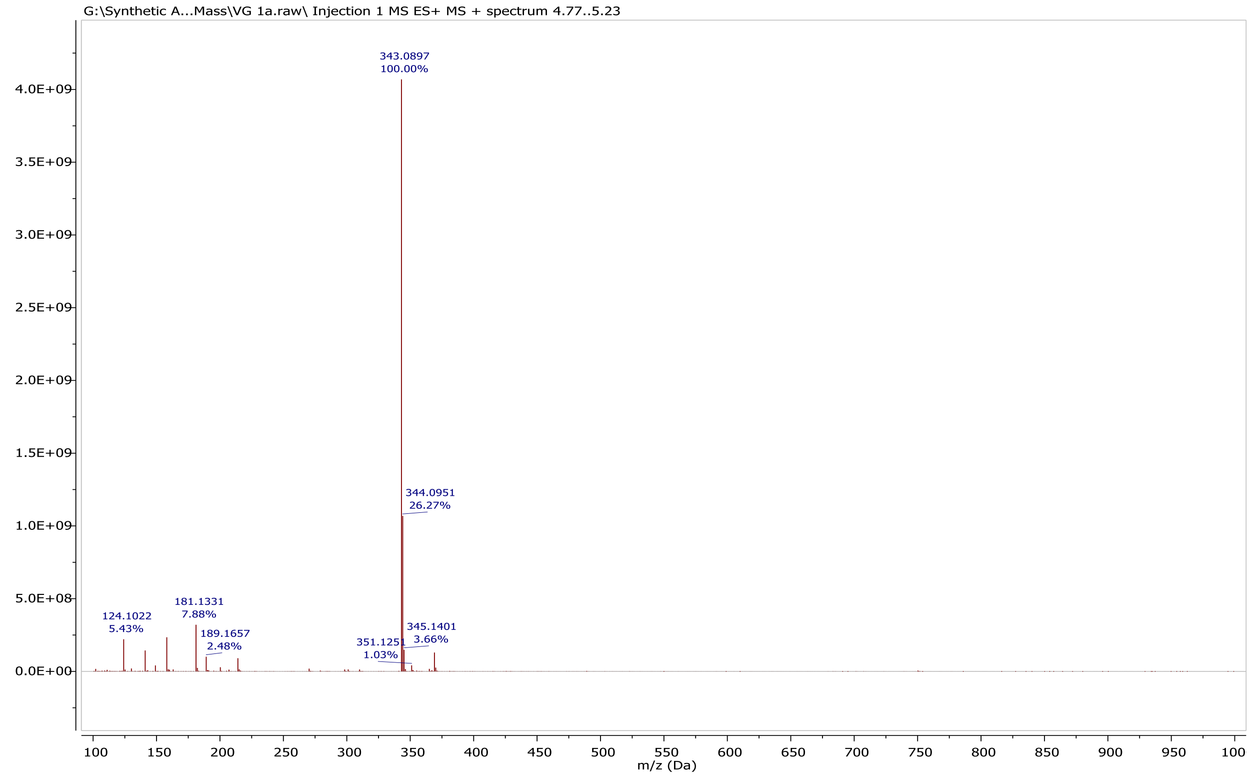

Figure S13. ESIMS spectrum of chalcone (2a).

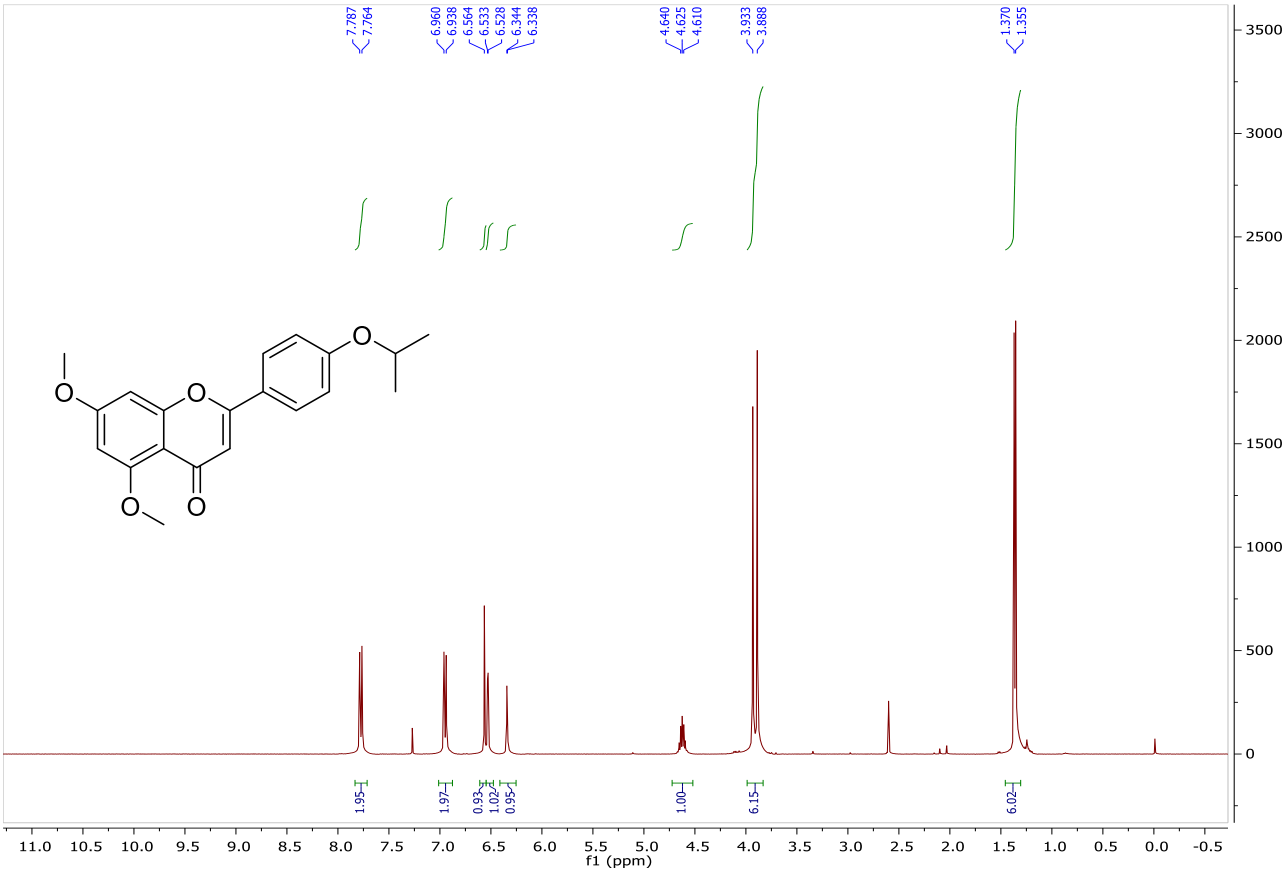

Figure S14. ^{1}H NMR spectrum of flavonoid (2b) in $CDCl_3$, 400 MHz

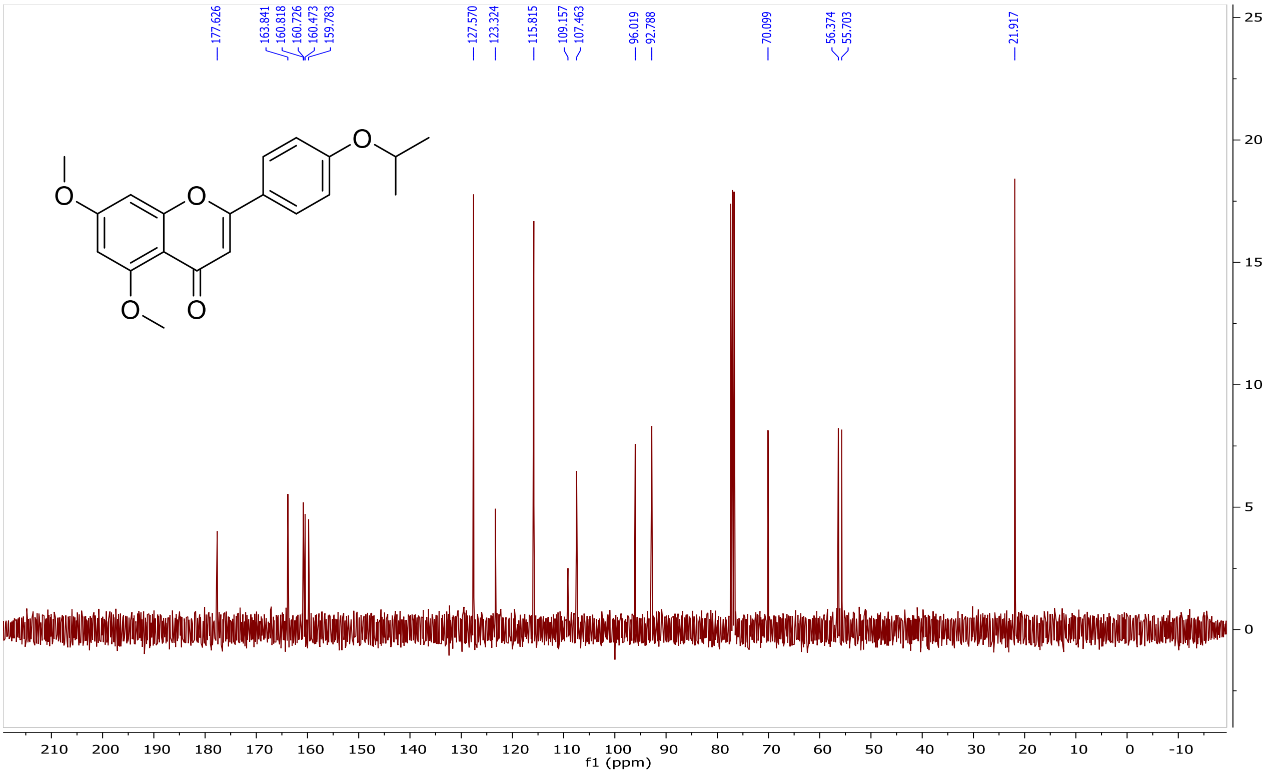

Figure S15. ^{13}C NMR spectrum of flavonoid (2b) in $CDCl_3$, 100 MHz

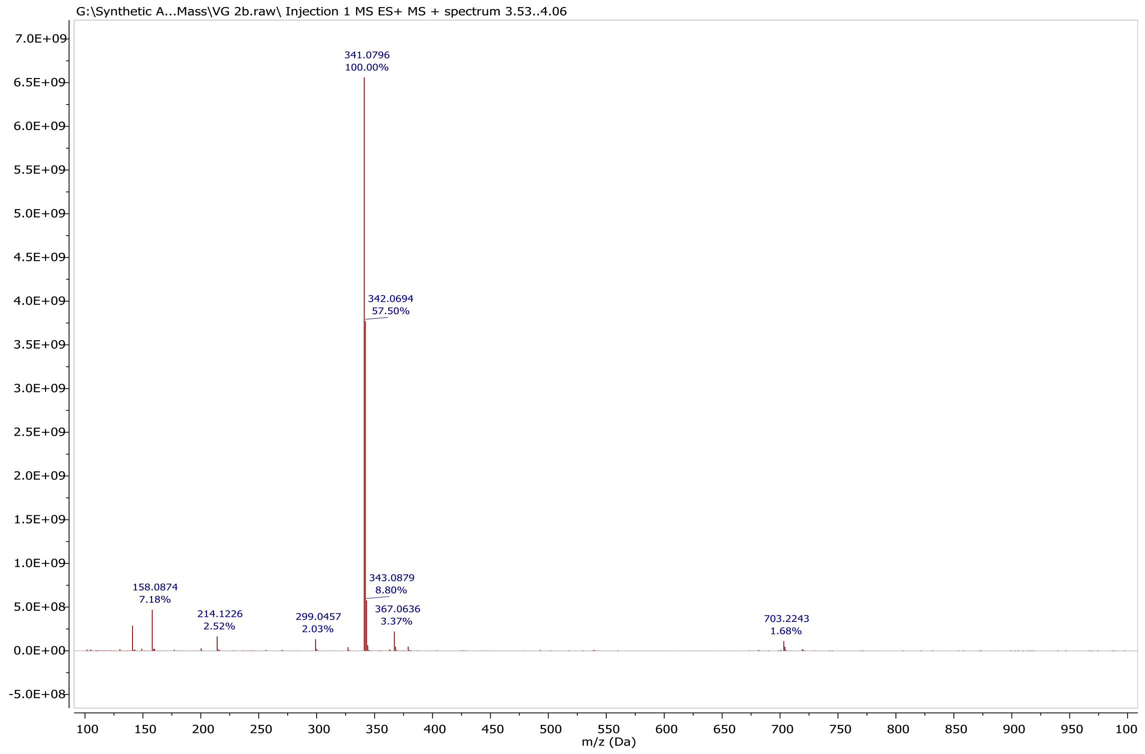

Figure S16. ESIMS spectrum of flavonoid (2b).

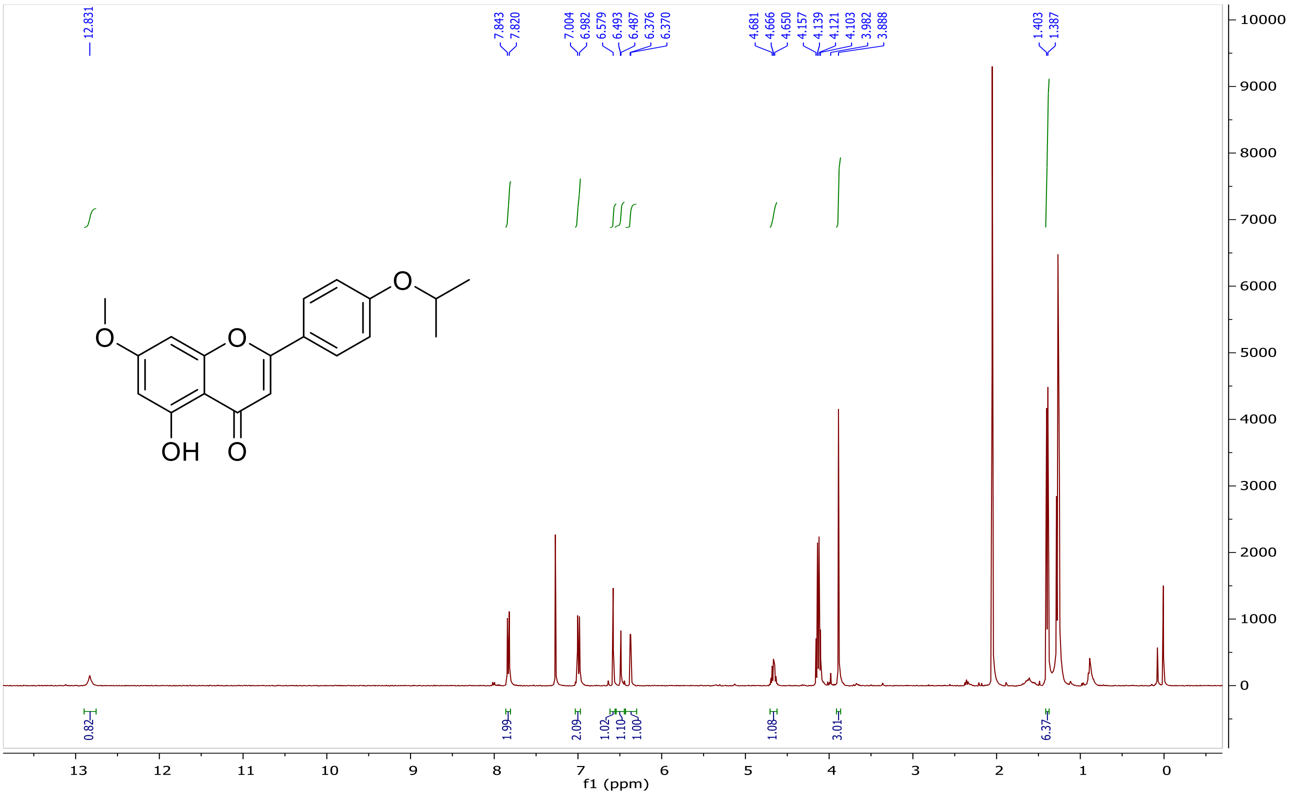

Figure S17. ^{1}**H NMR spectrum of flavonoid (2c) in CDCl$_3$, 400 MHz**

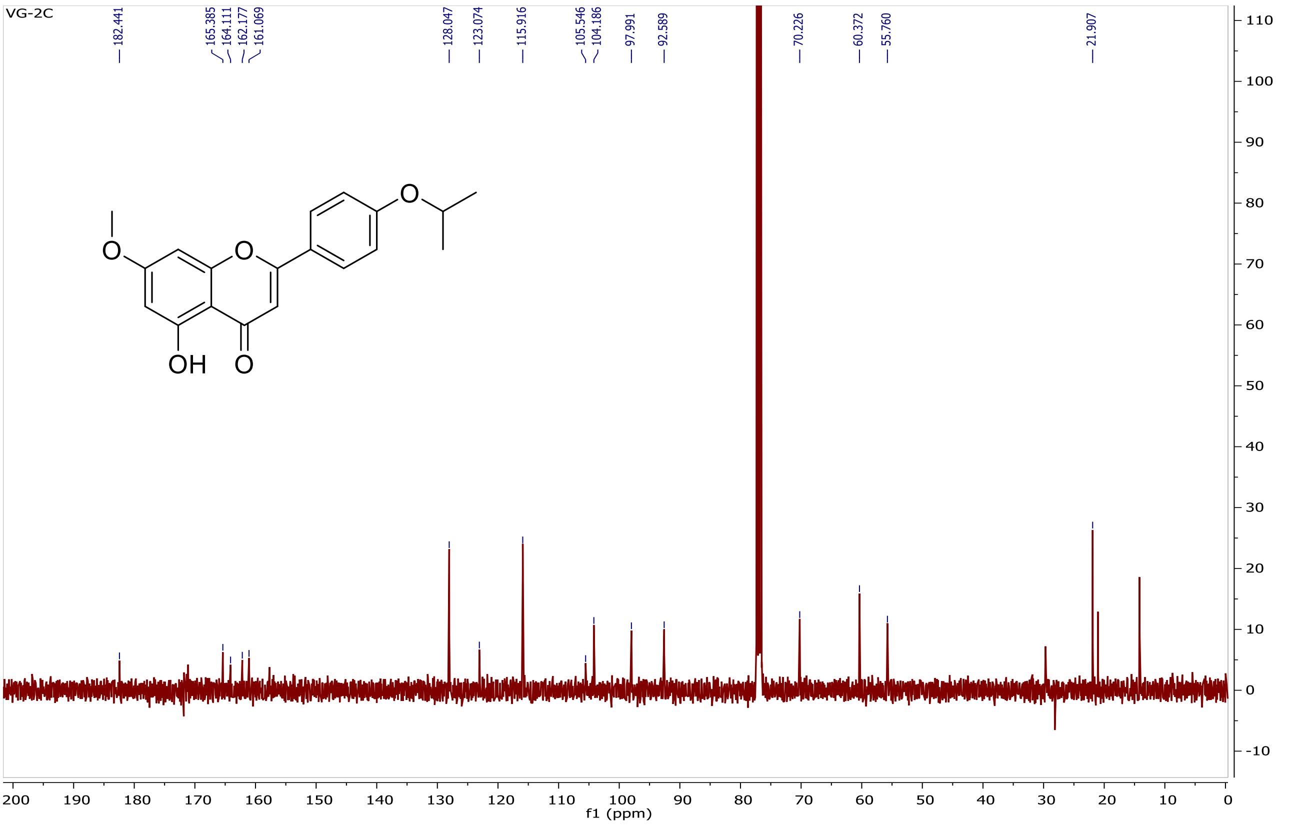

Figure S18. ^{13}**C NMR spectrum of flavonoid (2c) in CDCl$_3$, 100 MHz**

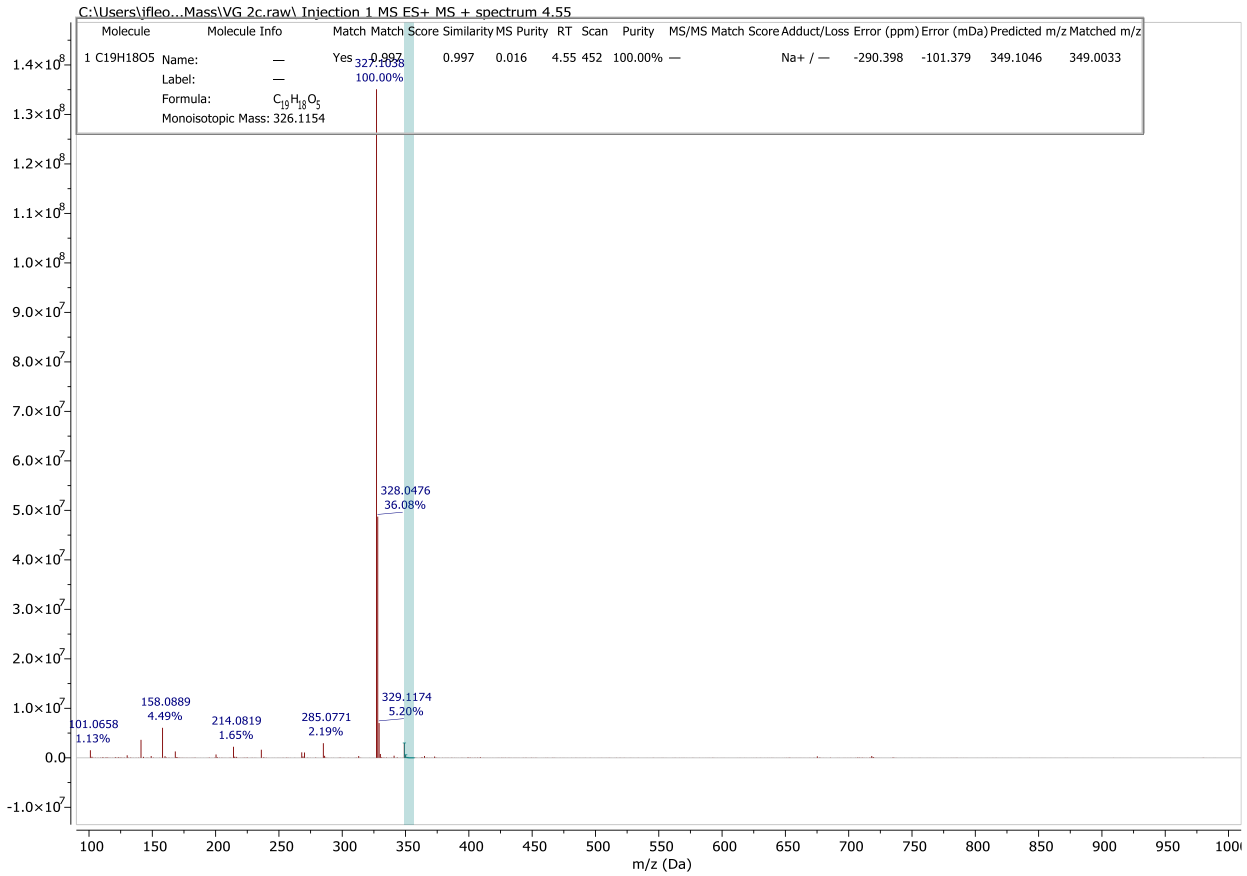

Figure S19. ESIMS spectrum of flavonoid (2c).

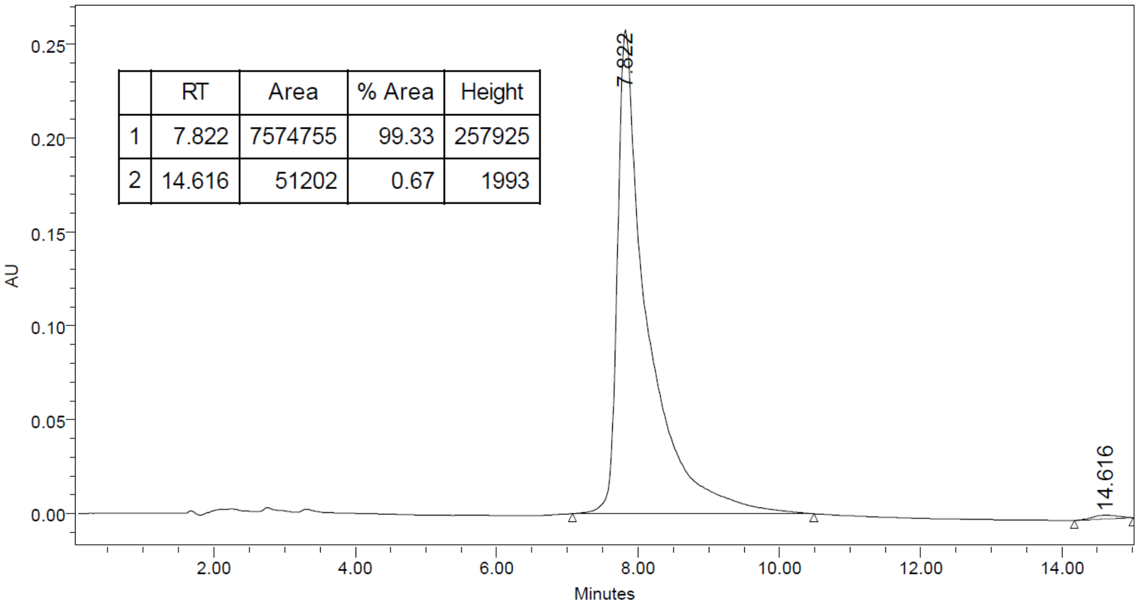

Figure S20. HPLC analysis of flavonoid (2c).

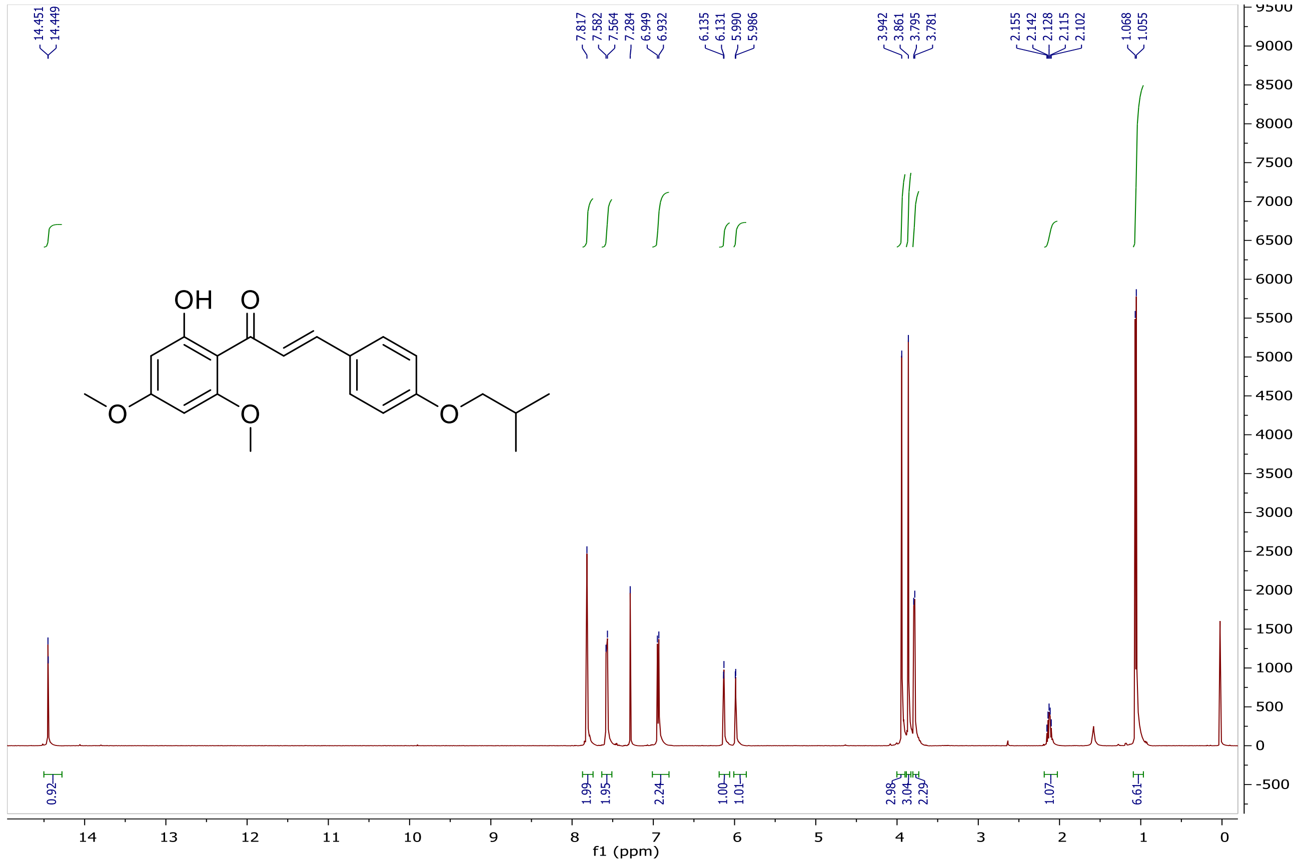

Figure S21. ^{1}H NMR spectrum chalcone (3a) in $CDCl_3$, 500 MHz

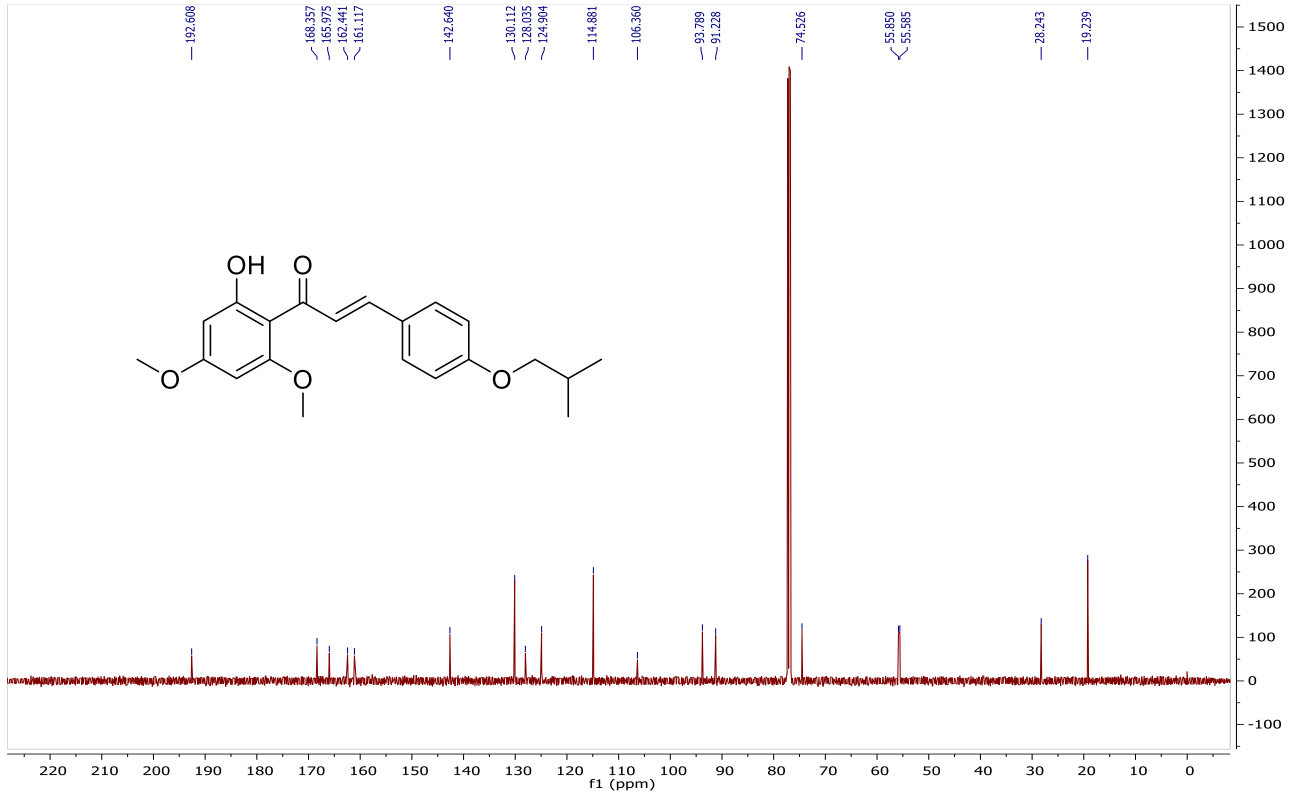

Figure S22. ^{13}C NMR spectrum of chalcone (3a) in $CDCl_3$, 125 MHz

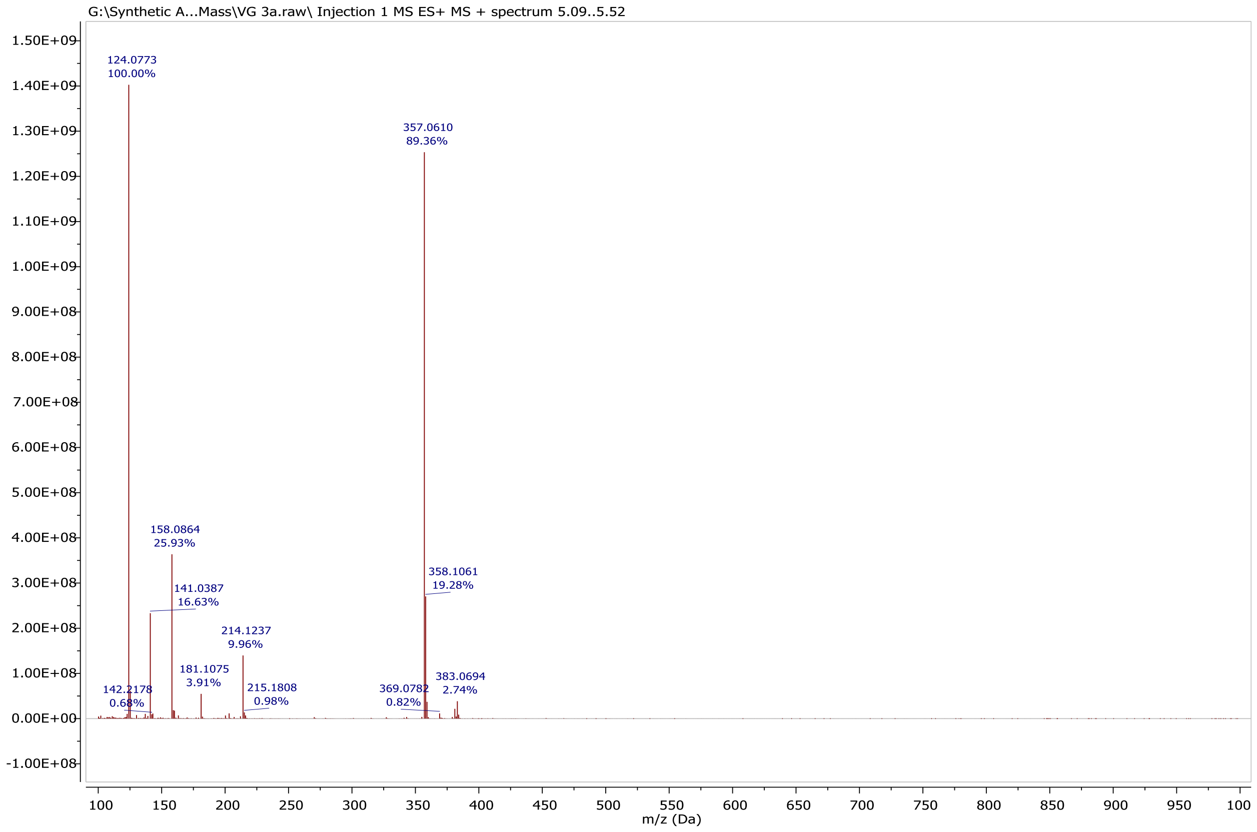

Figure S23. ESIMS spectrum of chalcone (3a).

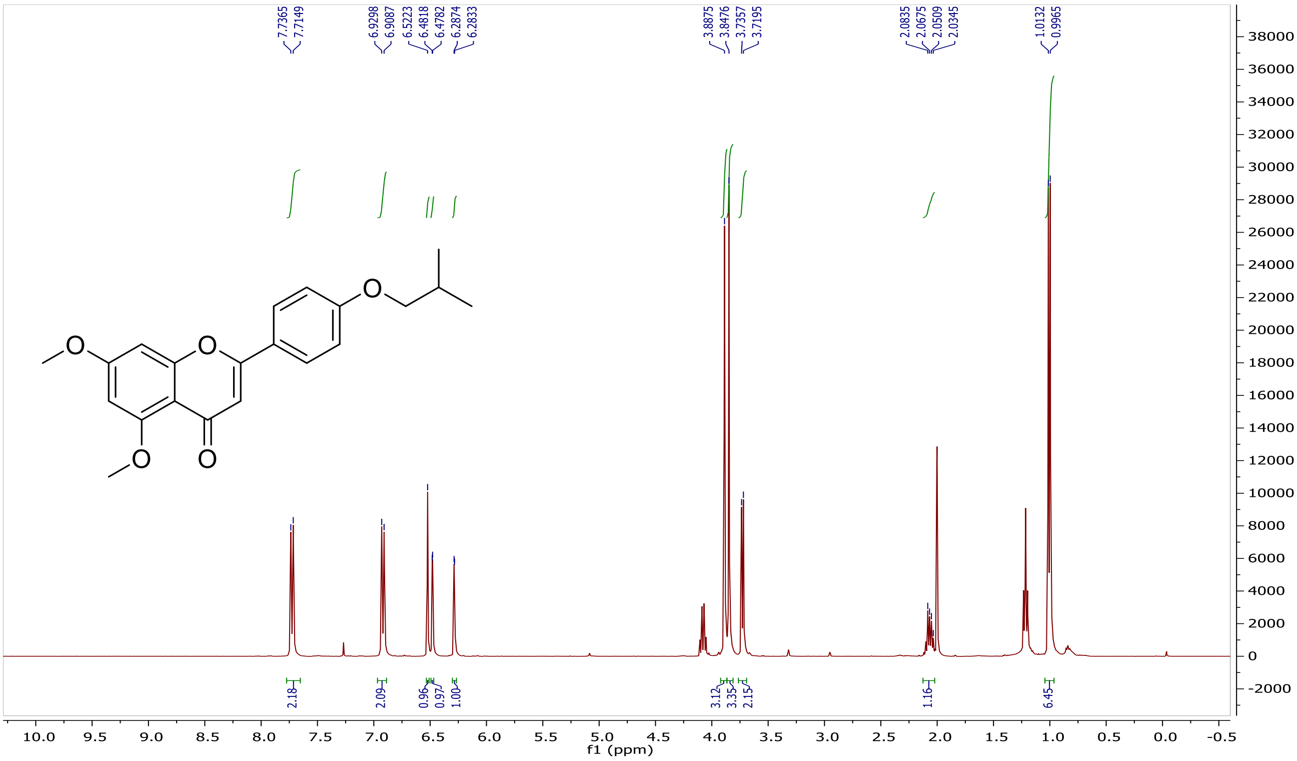

Figure S24. ^{1}H NMR spectrum of flavonoid (3b) in $CDCl_3$, 400 MHz

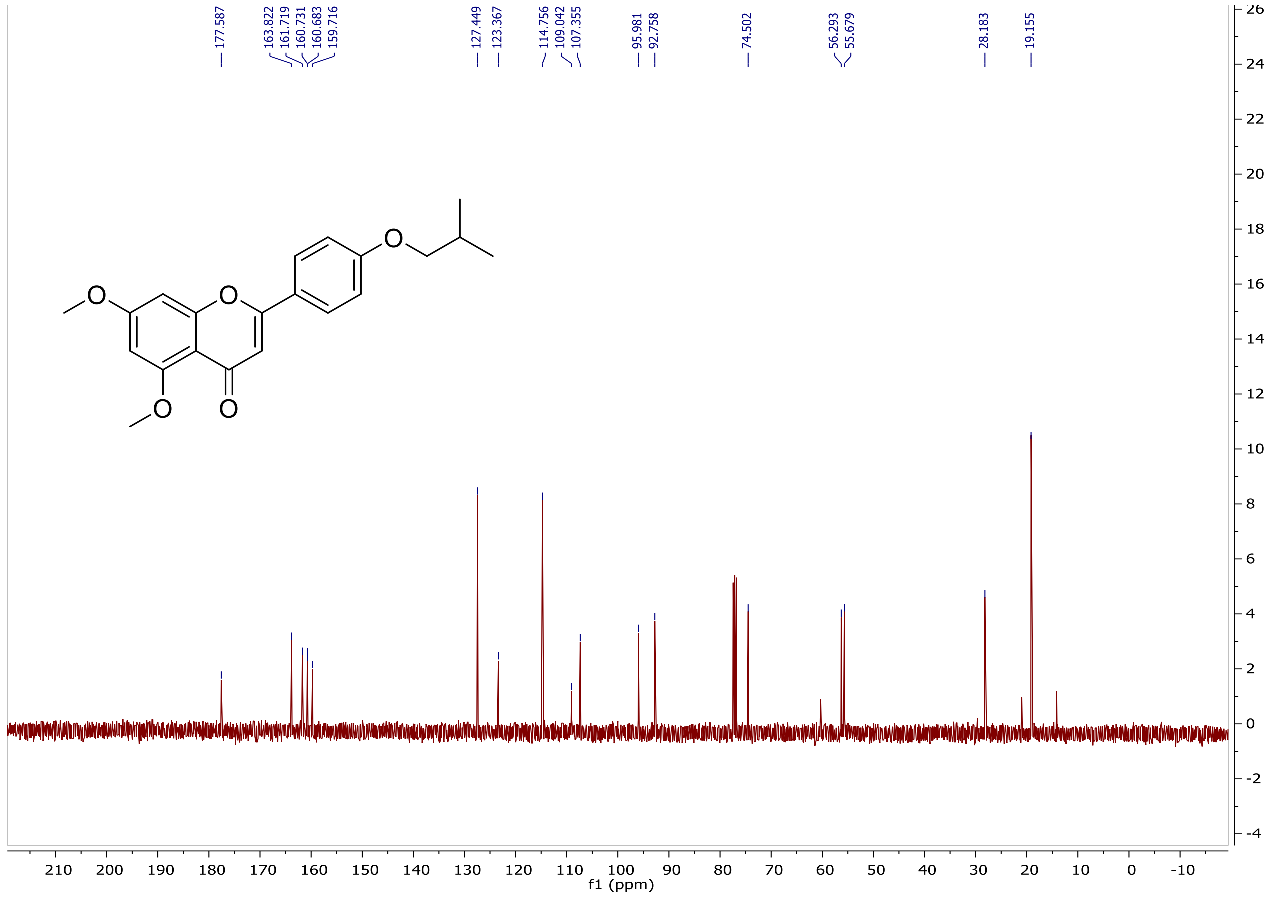

Figure S25. ^{13}C NMR spectrum of flavonoid (3b) in $CDCl_3$, 100 MHz

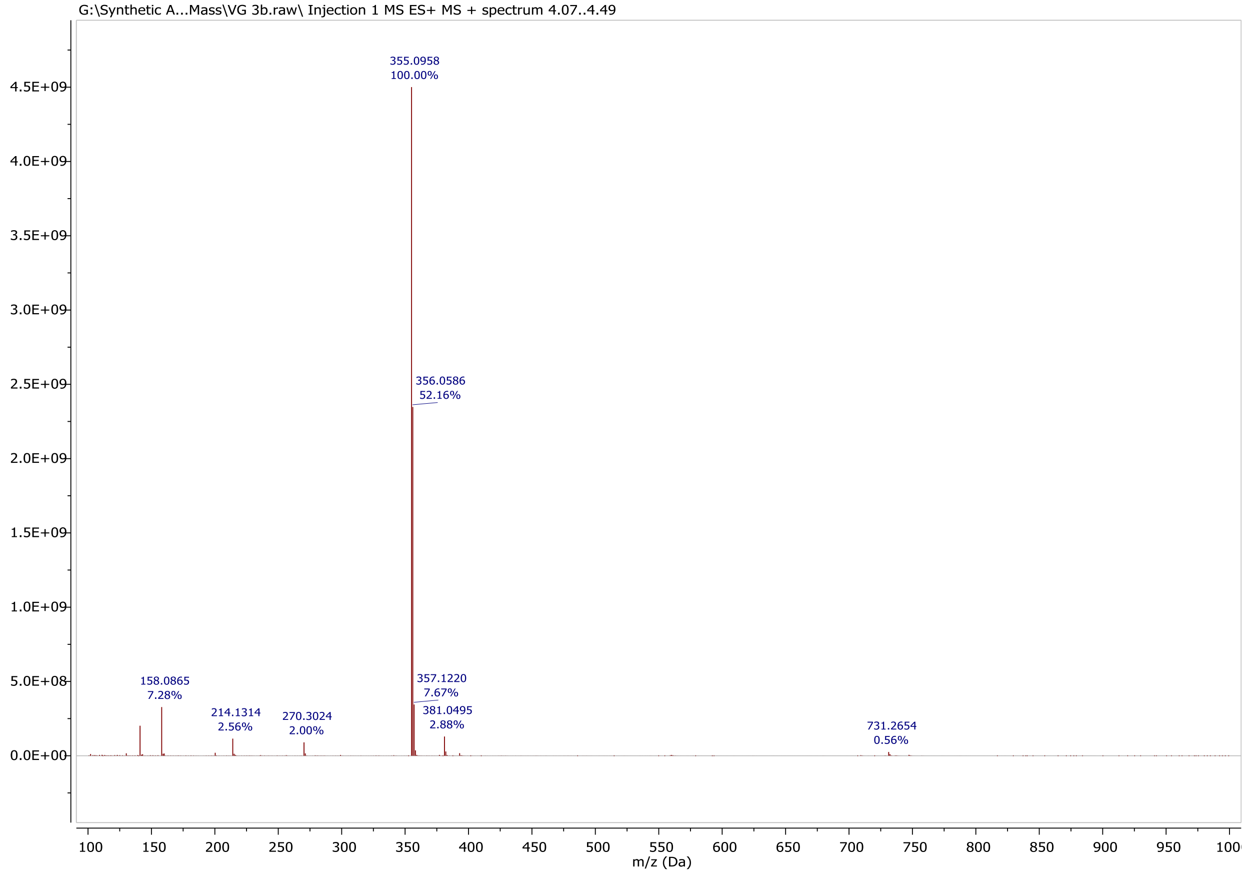

Figure S26. ESIMS spectrum of flavonoid (3b).

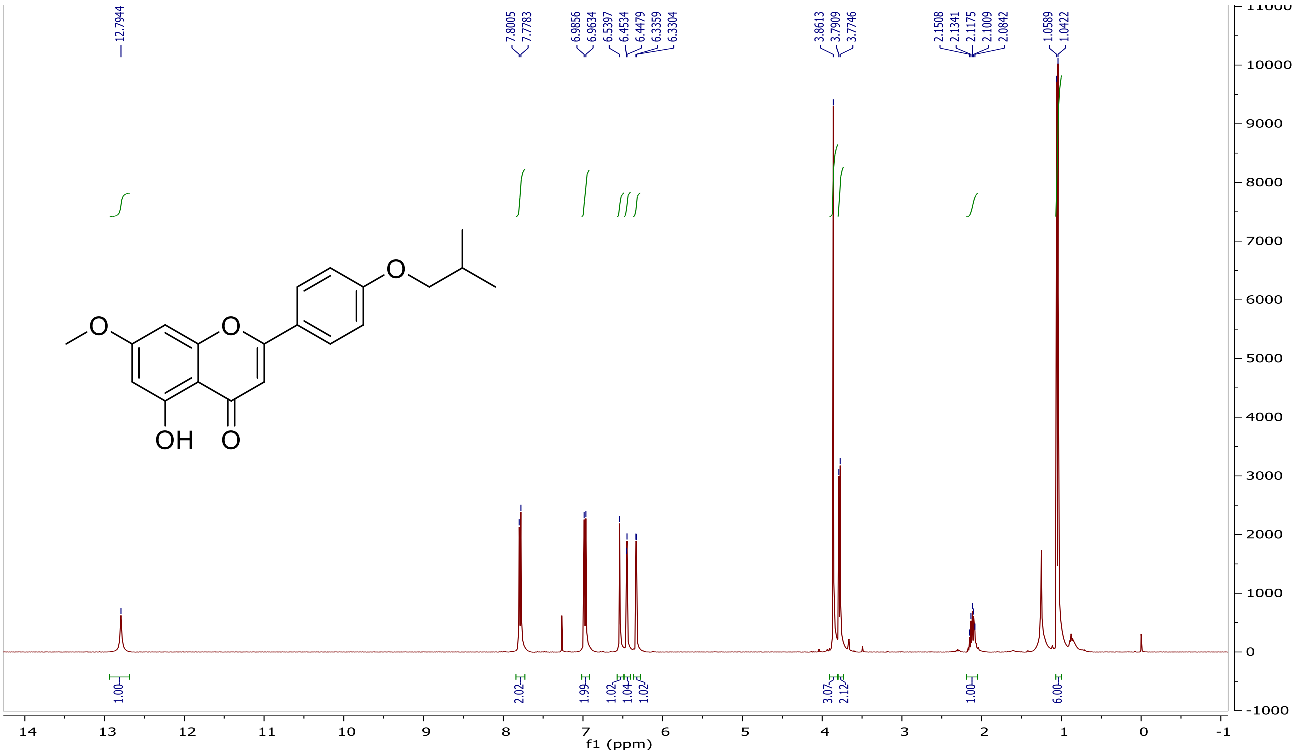

Figure S27. ^{1}H NMR spectrum of flavonoid (3c) in $CDCl_3$, 400 MHz

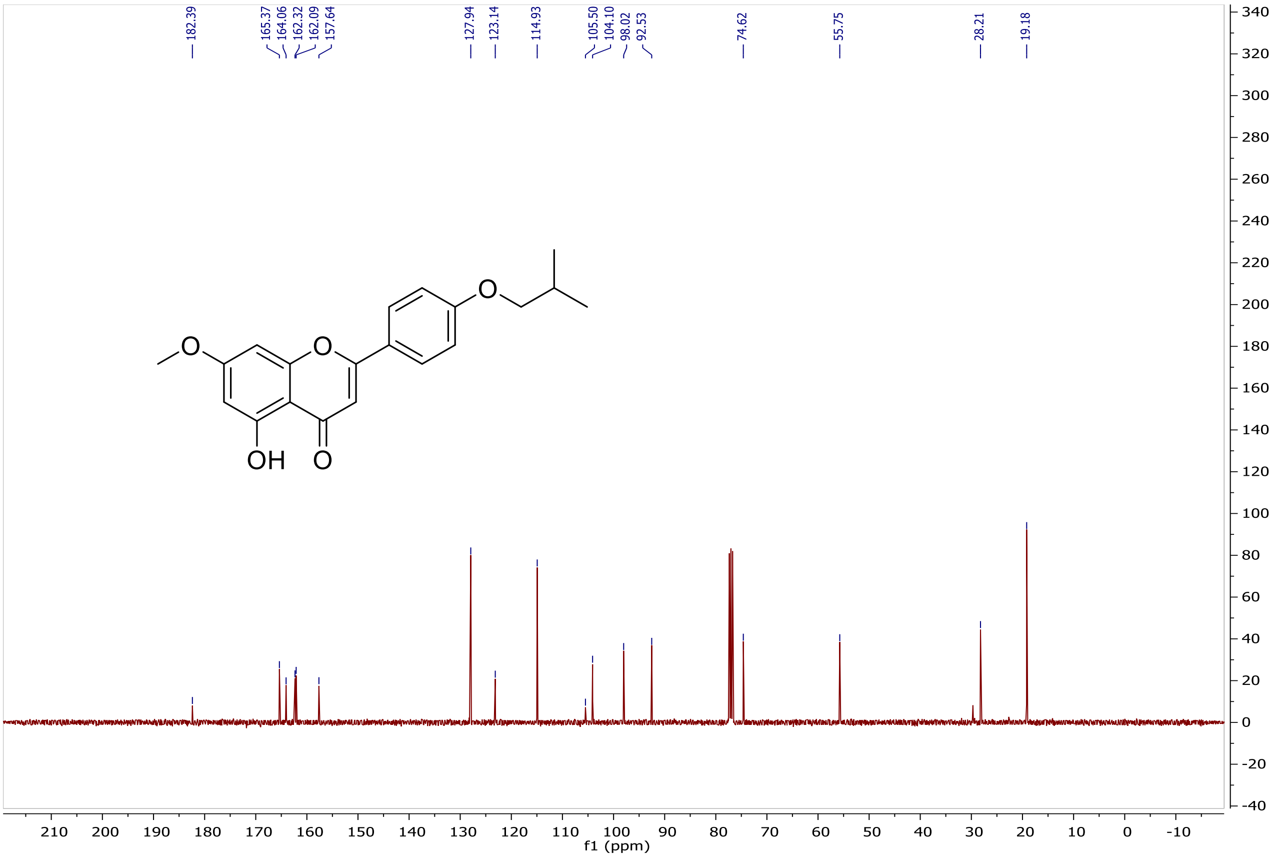

Figure S28. ^{13}C NMR spectrum of flavonoid (3c) in $CDCl_3$, 100 MHz

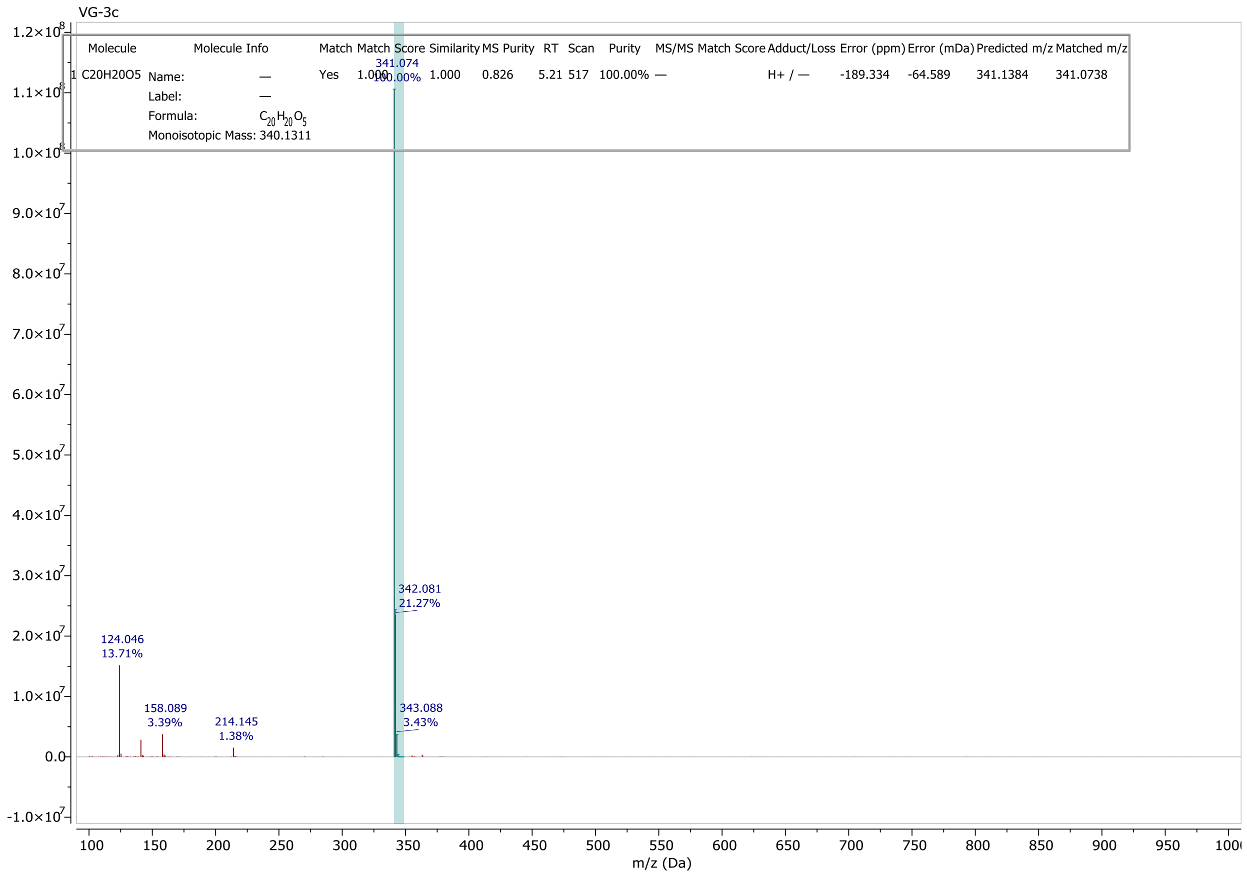

Figure S29. ESIMS spectrum of flavonoid (3c).

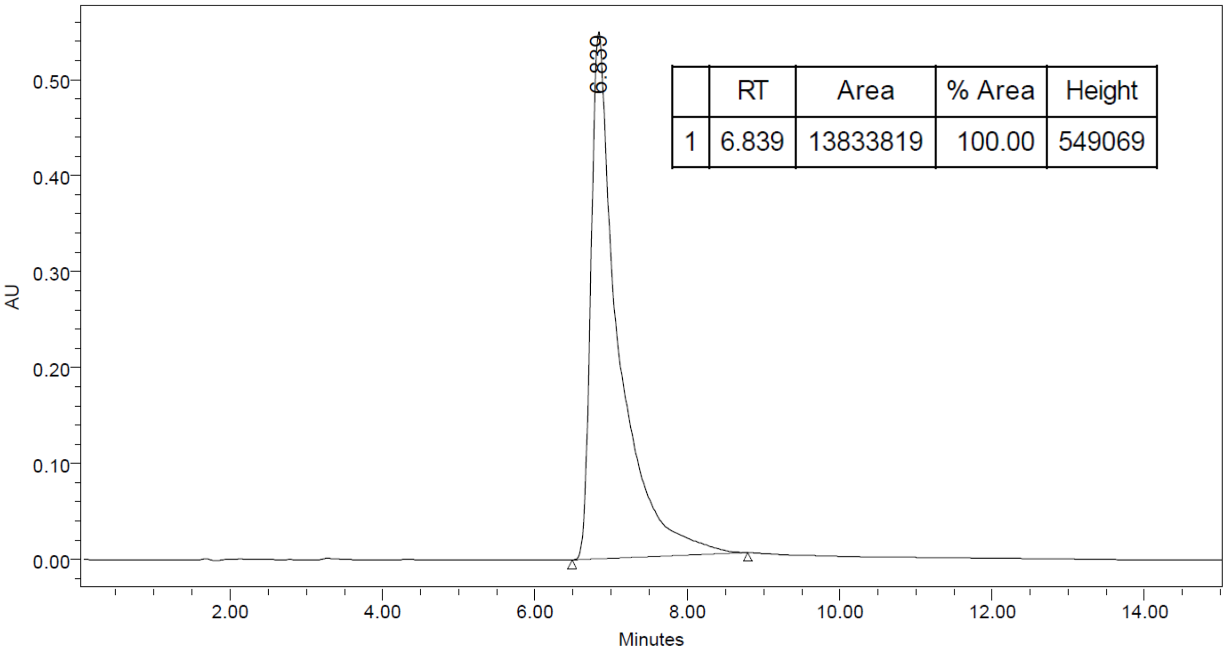

	RT	Area	% Area	Height
1	6.839	13833819	100.00	549069

Figure S30. HPLC analysis of flavonoid (3c).

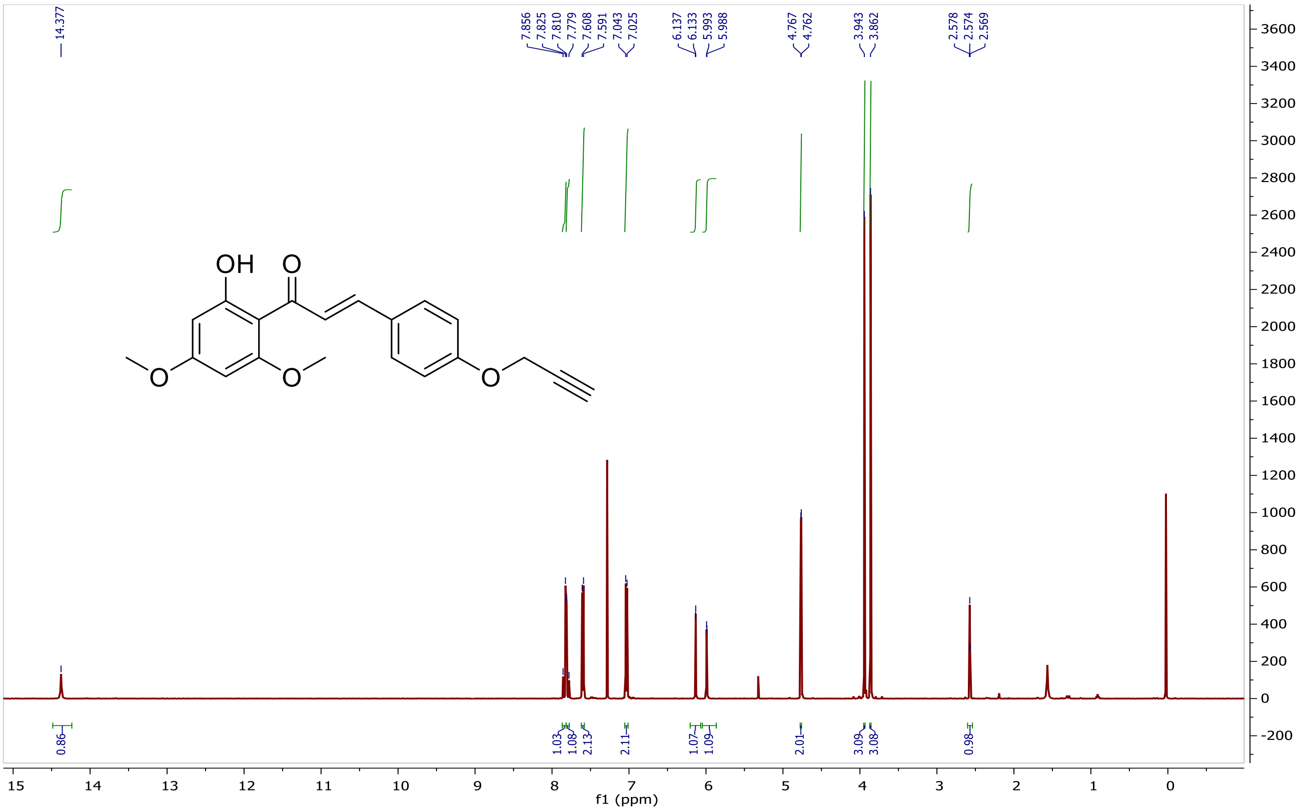

Figure S31. ^{1}H NMR spectrum of chalcone (4a) in $CDCl_3$, 500 MHz

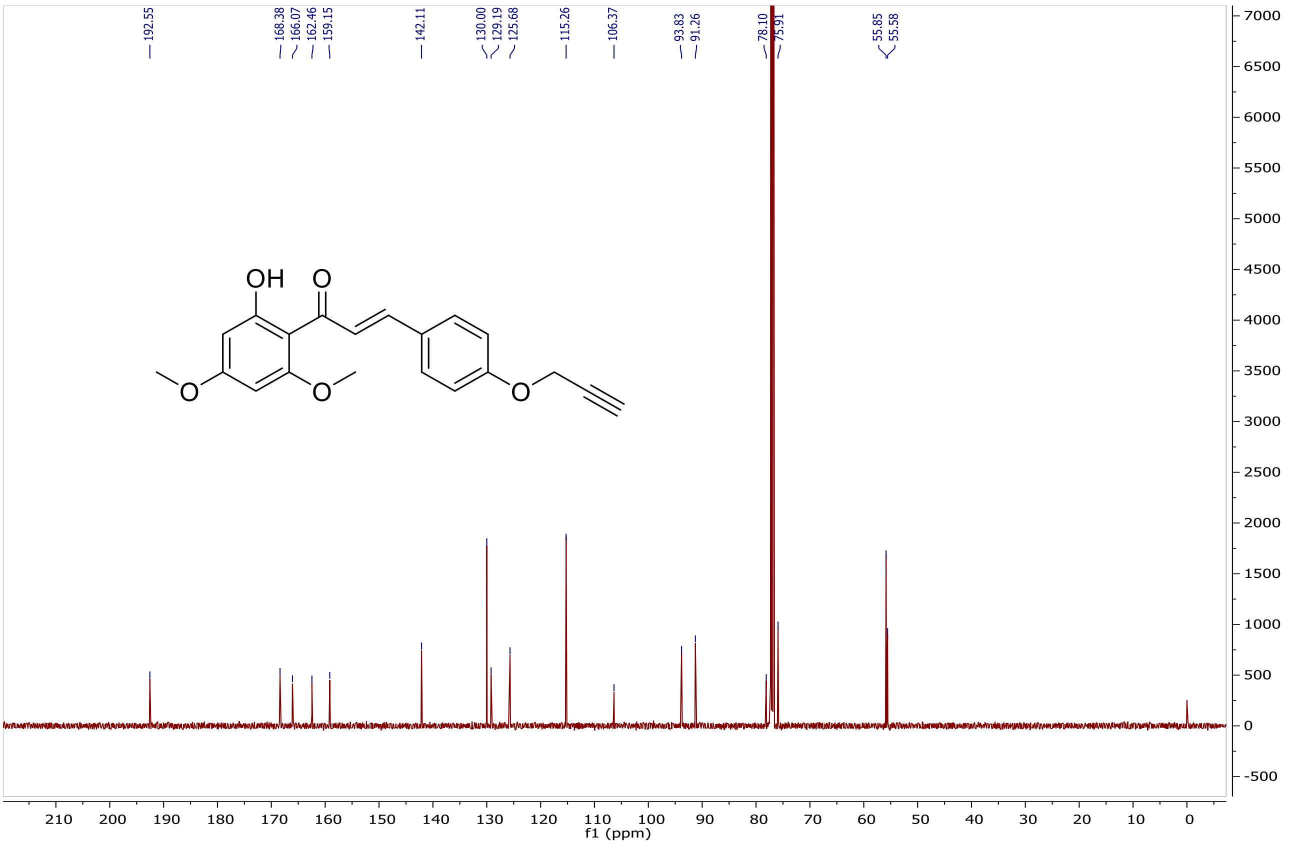

Figure S32. ^{13}C NMR spectrum of chalcone (4a) in $CDCl_3$, 125 MHz

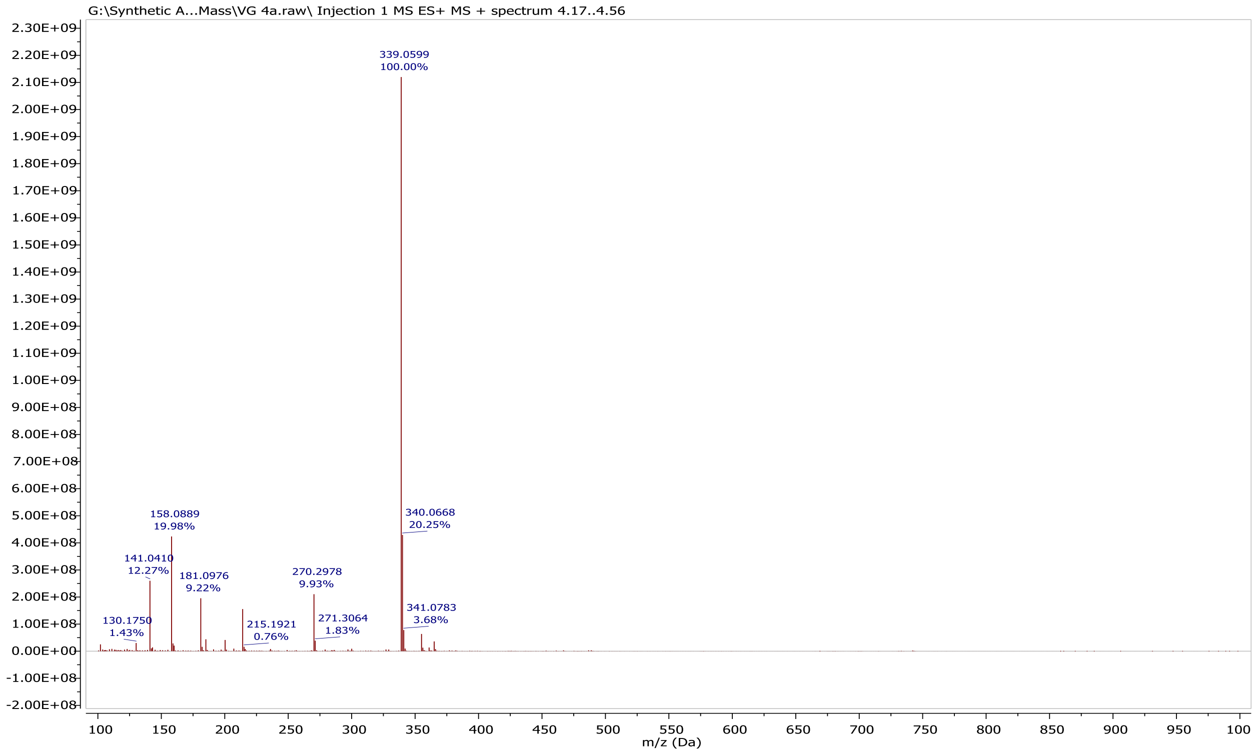

Figure S33. ESIMS spectrum of chalcone (4a).

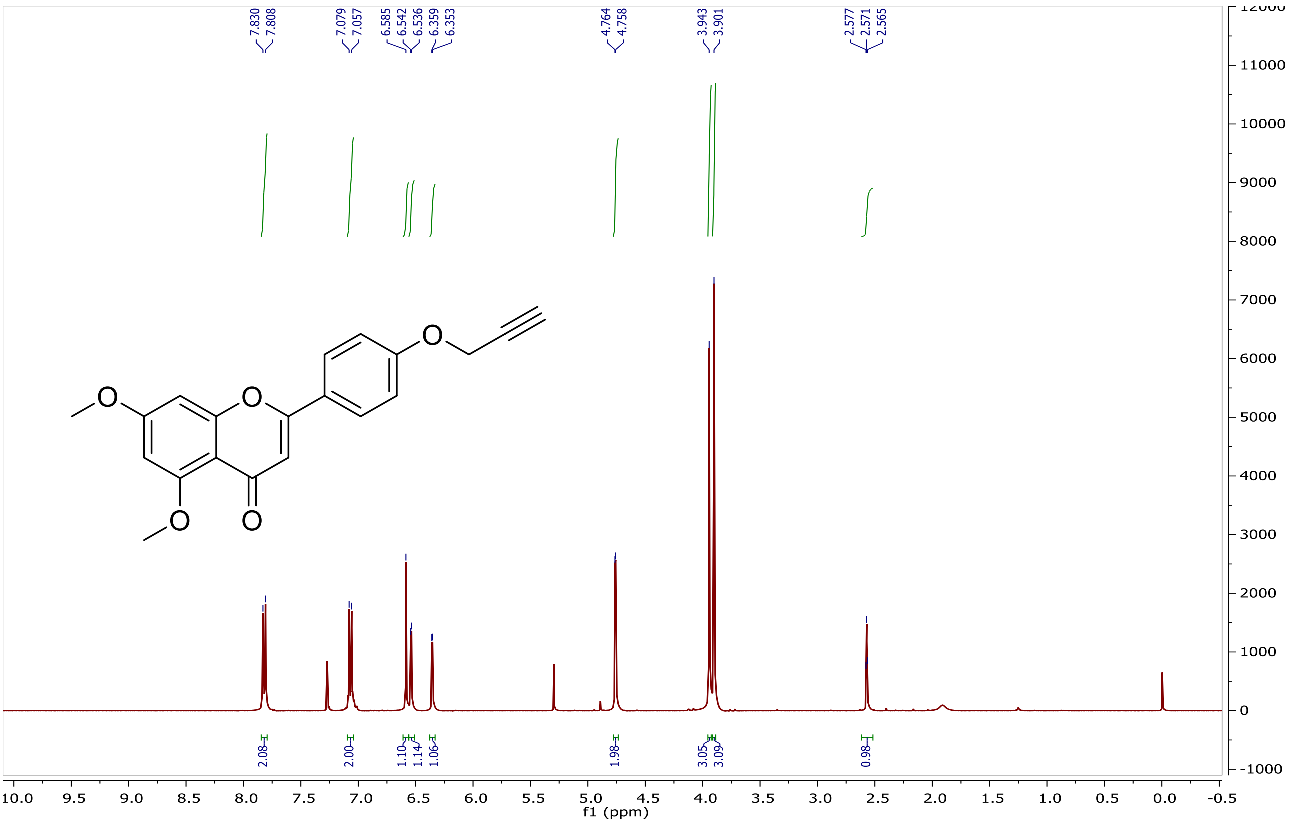

Figure S34. ^{1}H NMR spectrum of flavonoid (4b) in $CDCl_3$, 400 MHz

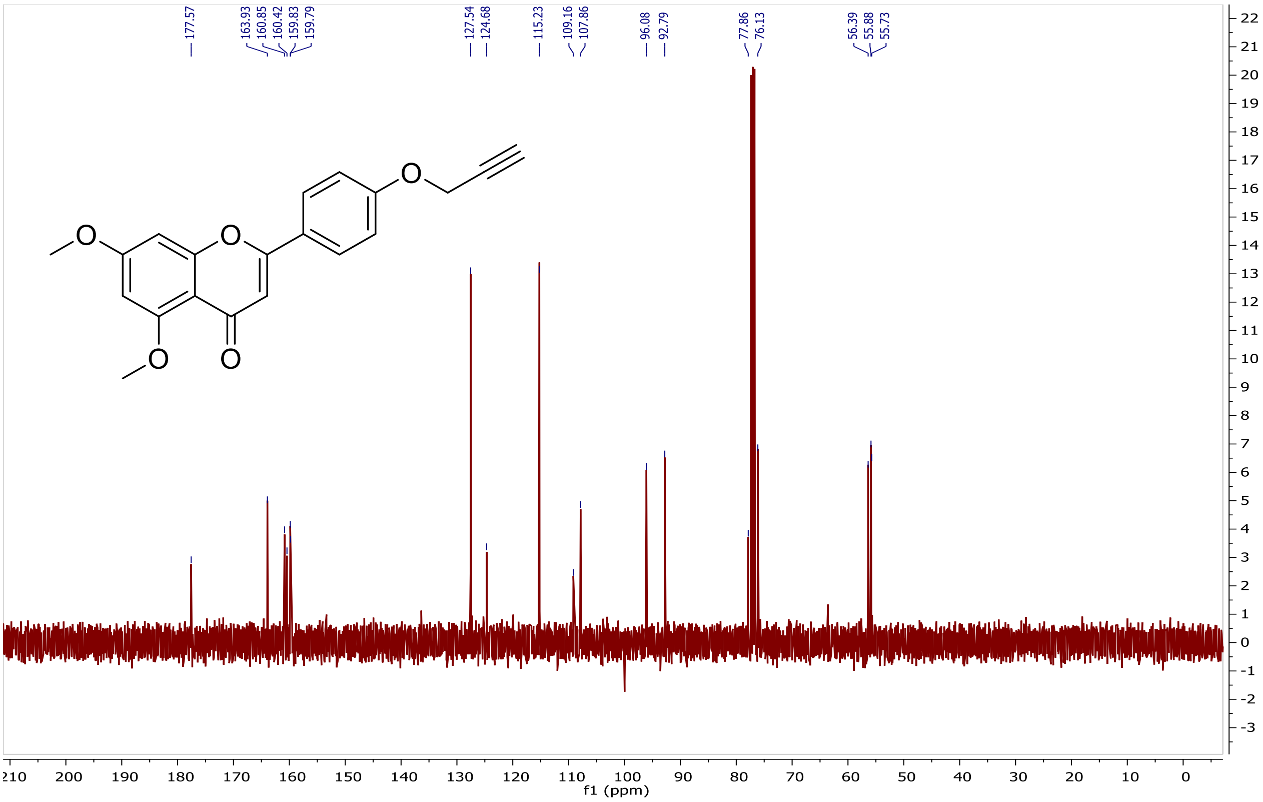

Figure S35. ^{13}C **NMR spectrum of flavonoid (4b) in** $CDCl_3$**, 100 MHz**

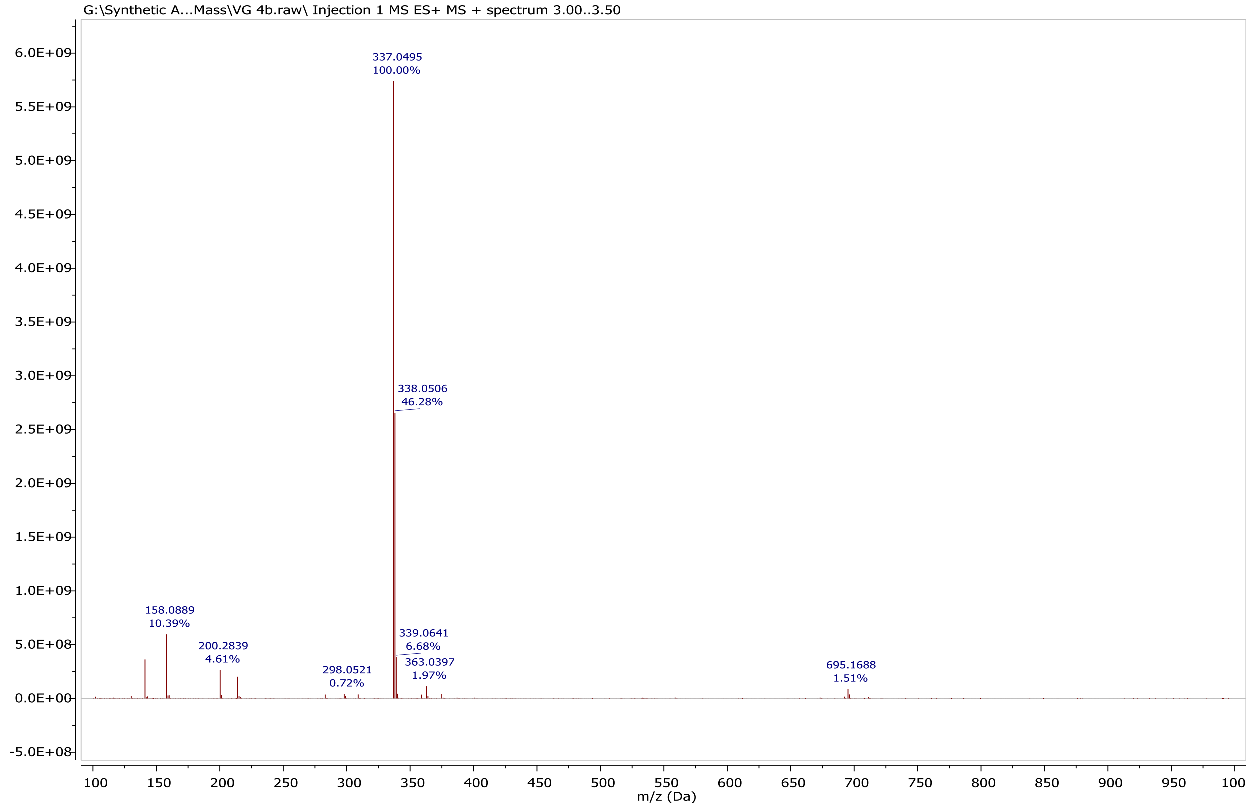

Figure S36. ESIMS spectrum of flavonoid (4b).

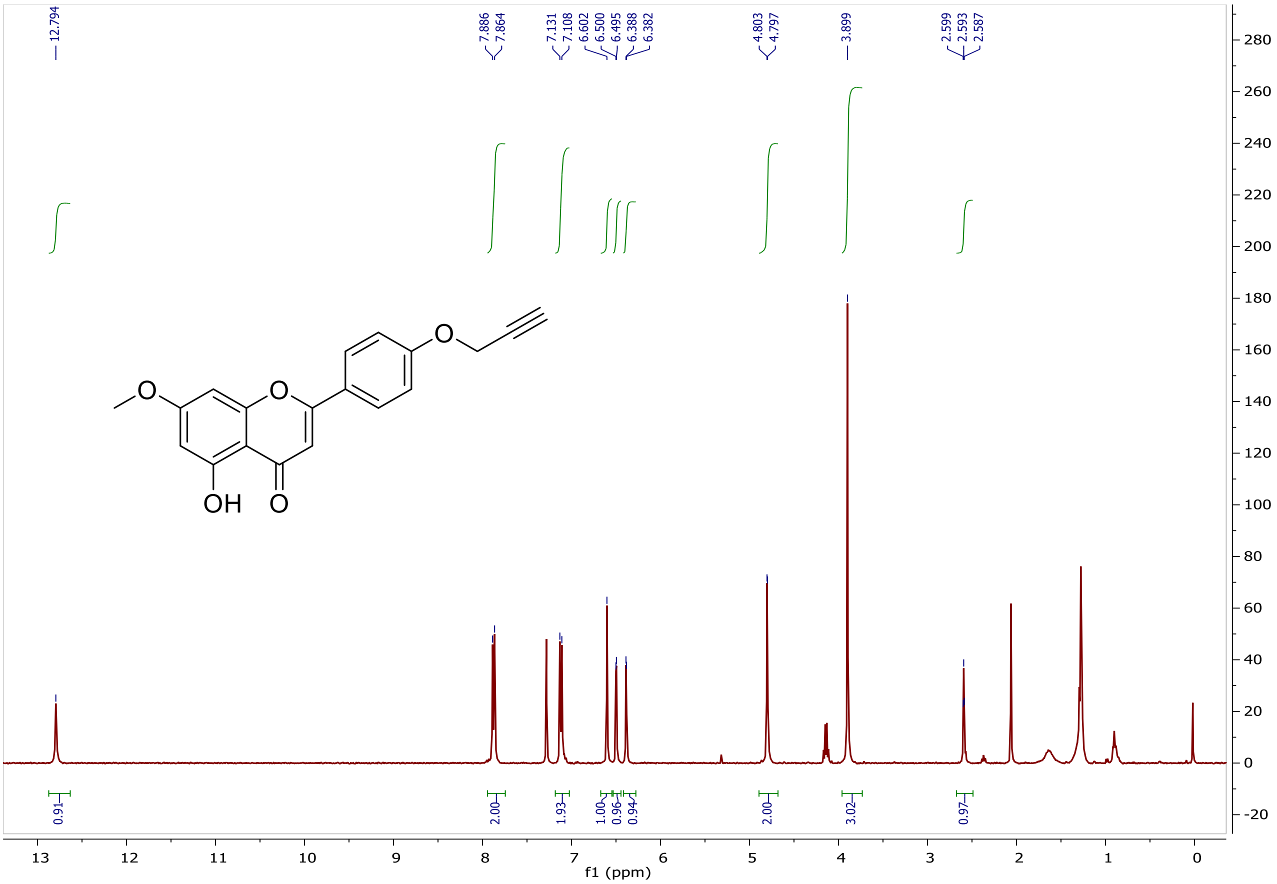

Figure S37. **^{1}H NMR spectrum of flavonoid (4c) in $CDCl_3$, 400 MHz**

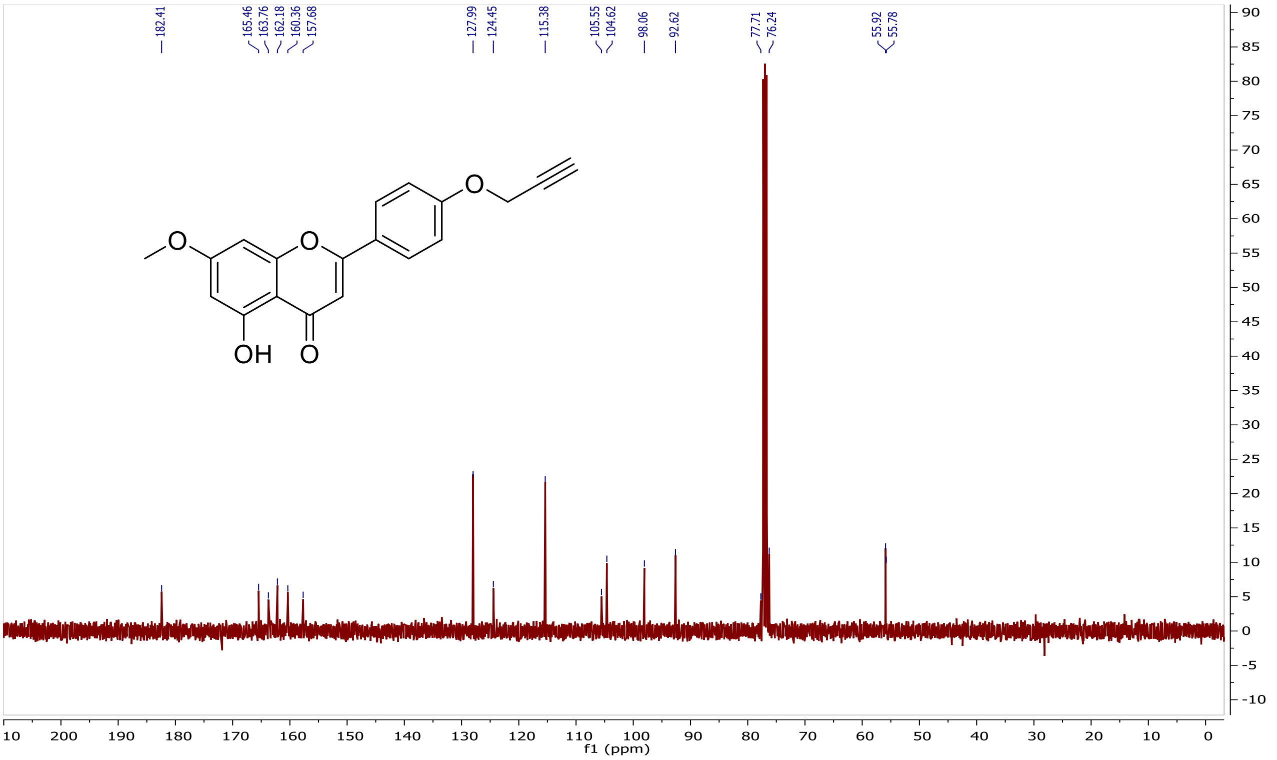

Figure S38. **^{13}C NMR spectrum of flavonoid (4c) in $CDCl_3$, 100 MHz**

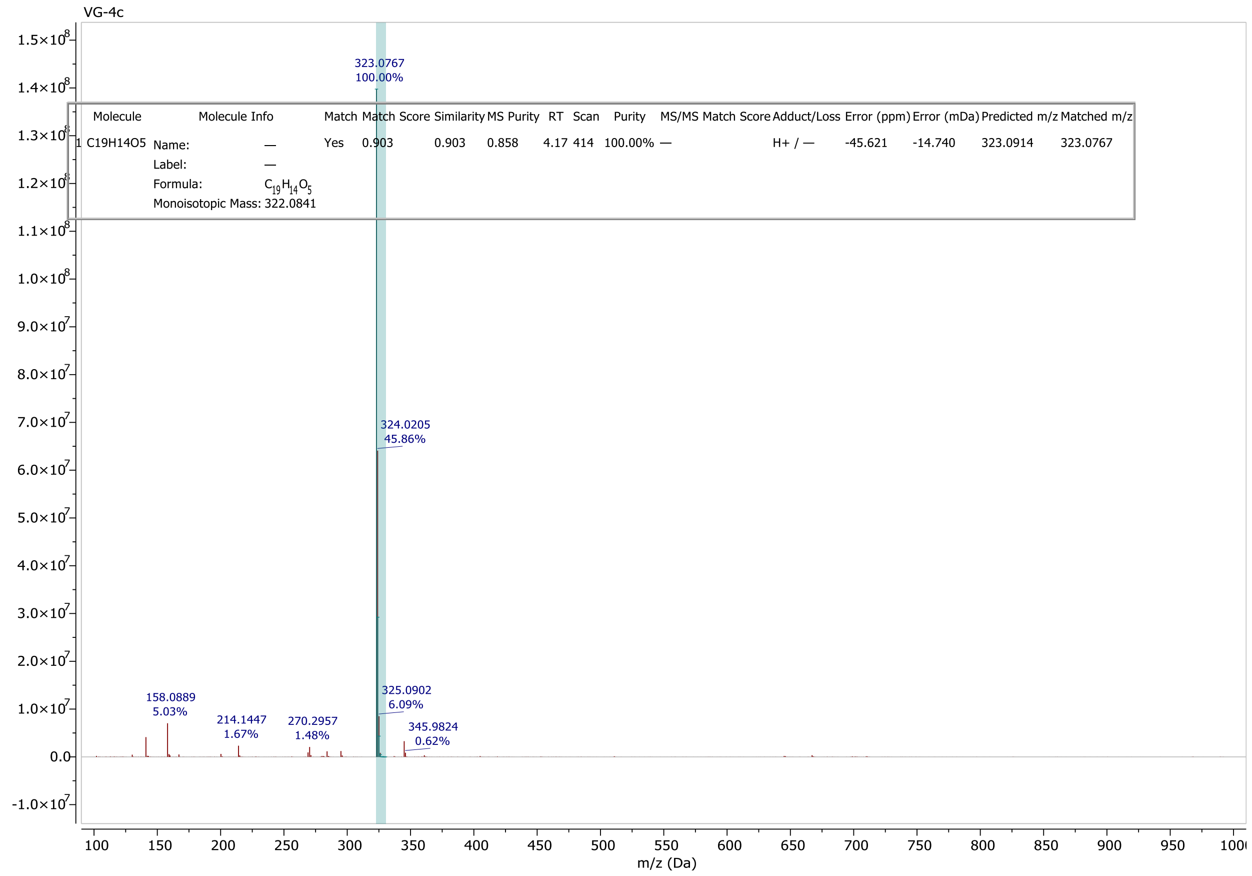

Figure S39. ESIMS spectrum of flavonoid (4c).

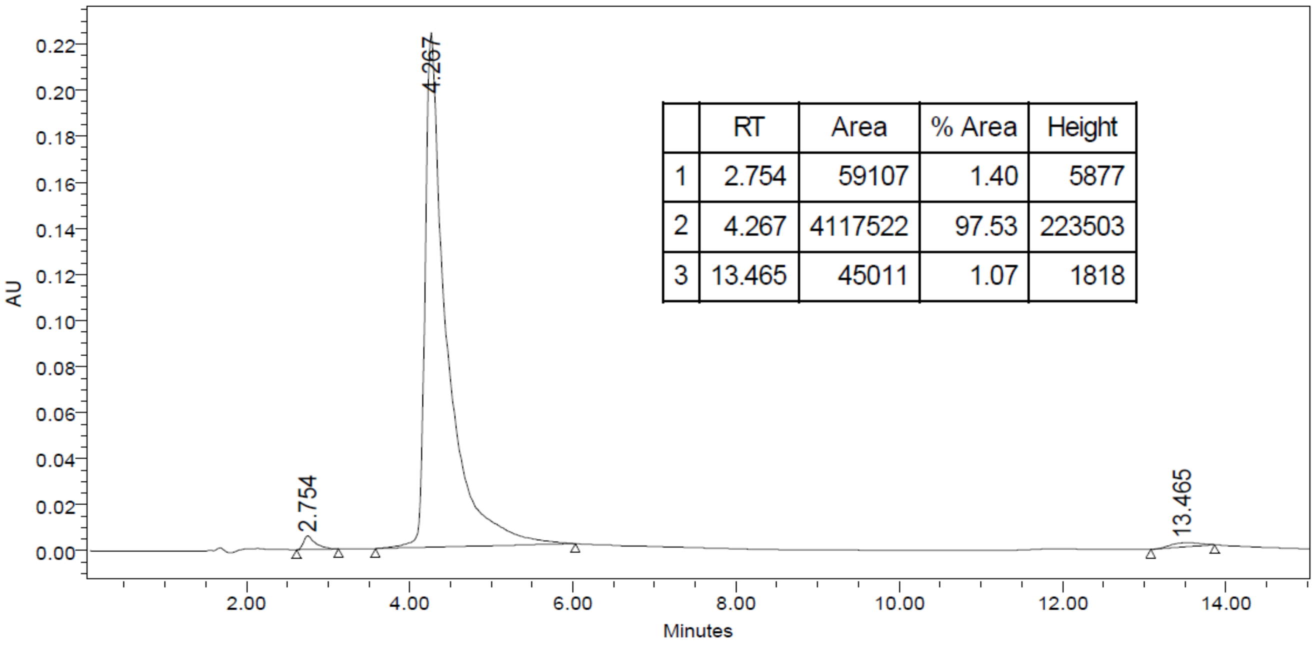

	RT	Area	% Area	Height
1	2.754	59107	1.40	5877
2	4.267	4117522	97.53	223503
3	13.465	45011	1.07	1818

Figure S40. HPLC analysis of flavonoid (4c).

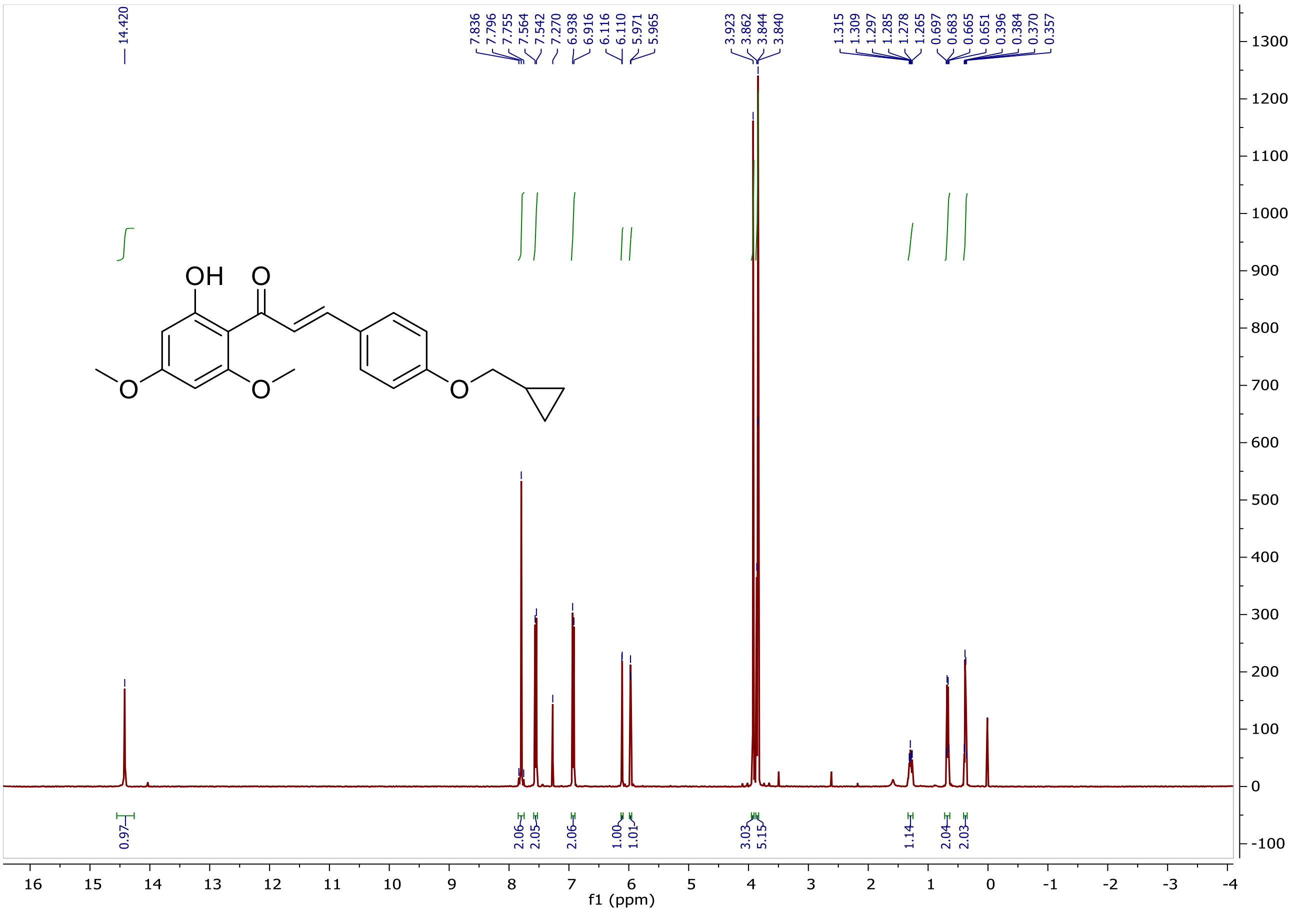

Figure S41. ^{1}H **NMR spectrum of chalcone (5a) in** $CDCl_3$**, 400 MHz**

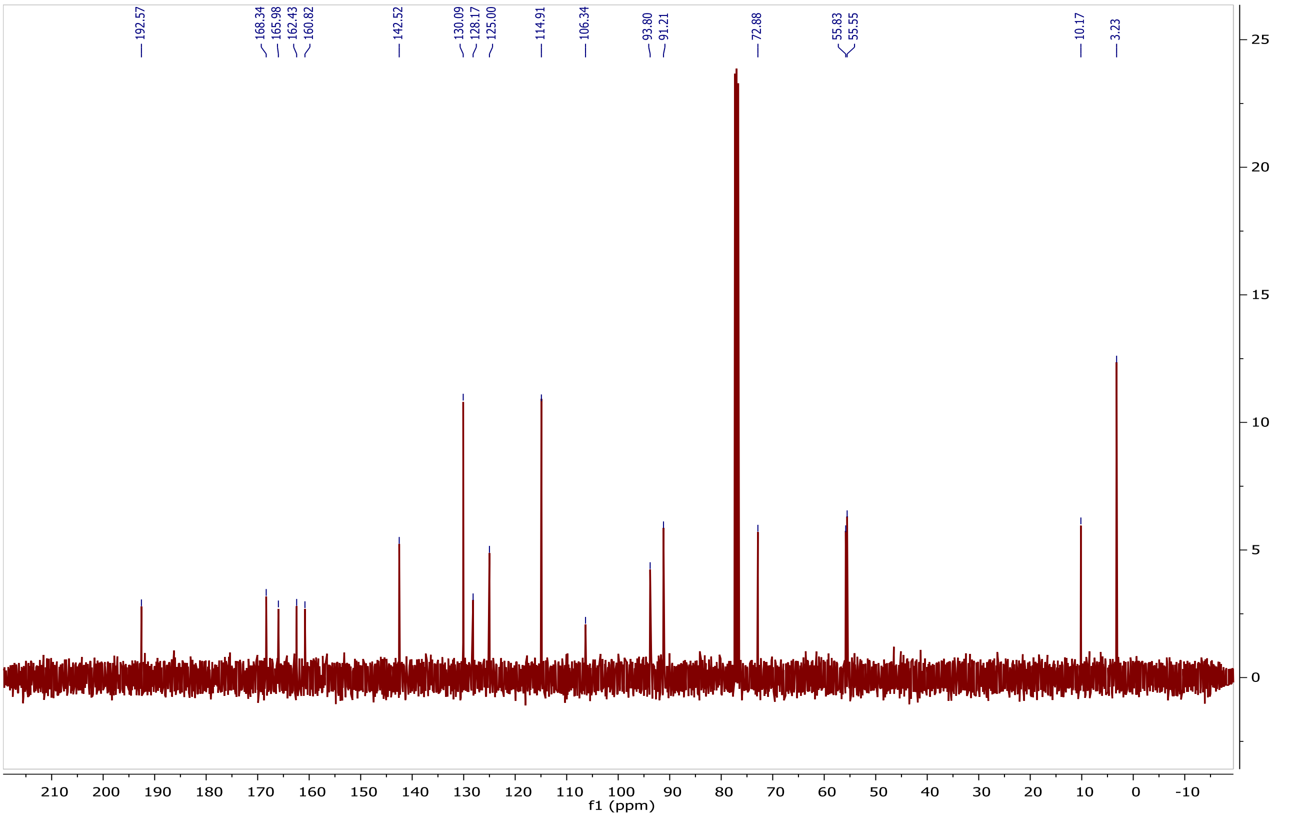

Figure S42. ^{13}C **NMR spectrum of chalcone (5a) in** $CDCl_3$**, 100 MHz**

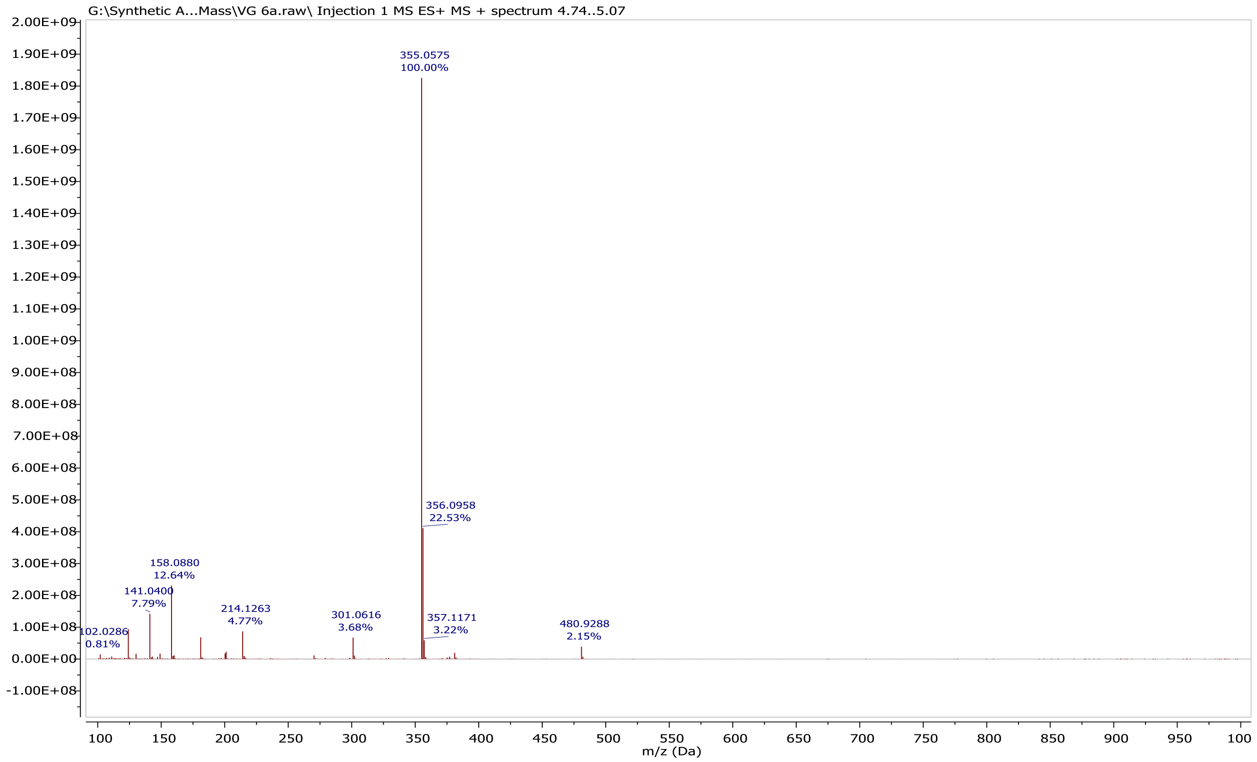

Figure S43. ESIMS spectrum of flavonoid (5a).

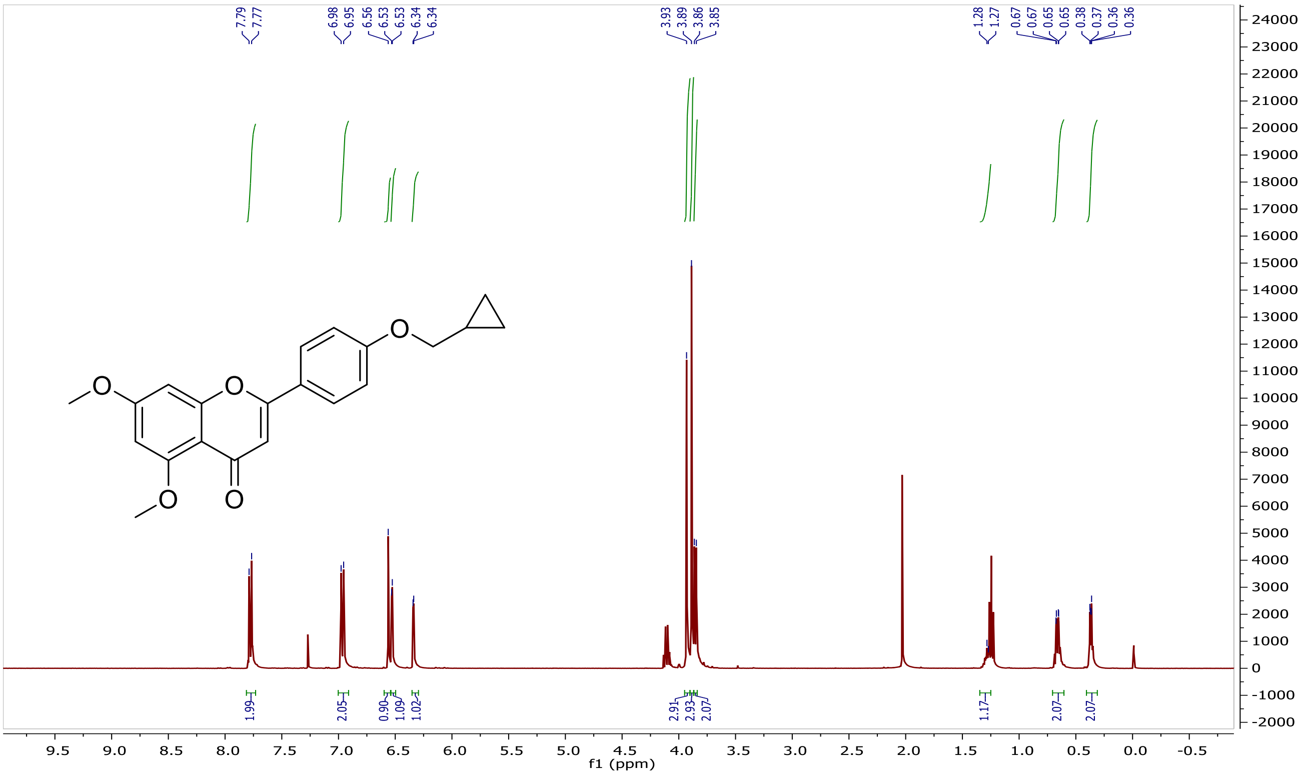

Figure S44. ^{1}H NMR spectrum of flavonoid (5b) in $CDCl_3$, 400 MHz

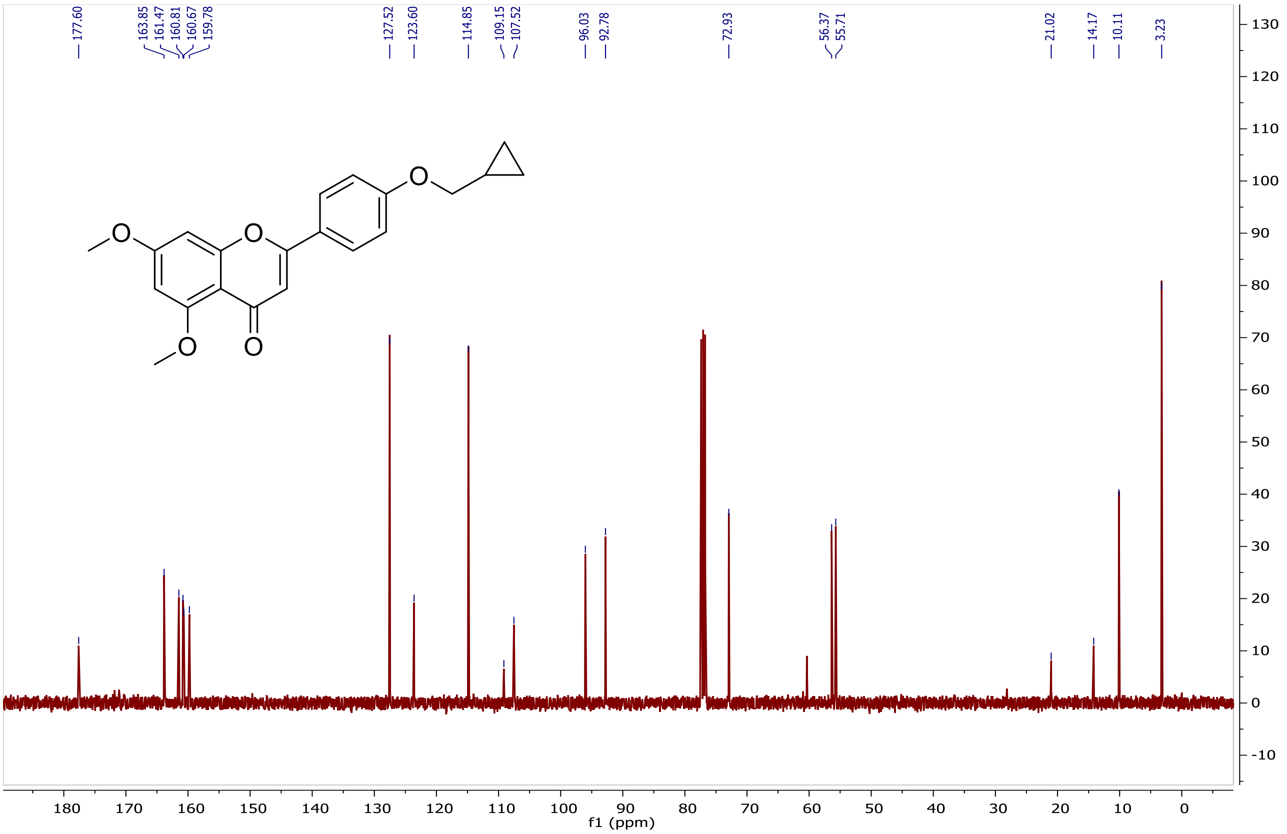

Figure S45. **^{13}C NMR spectrum of flavonoid (5b) in $CDCl_3$, 100 MHz**

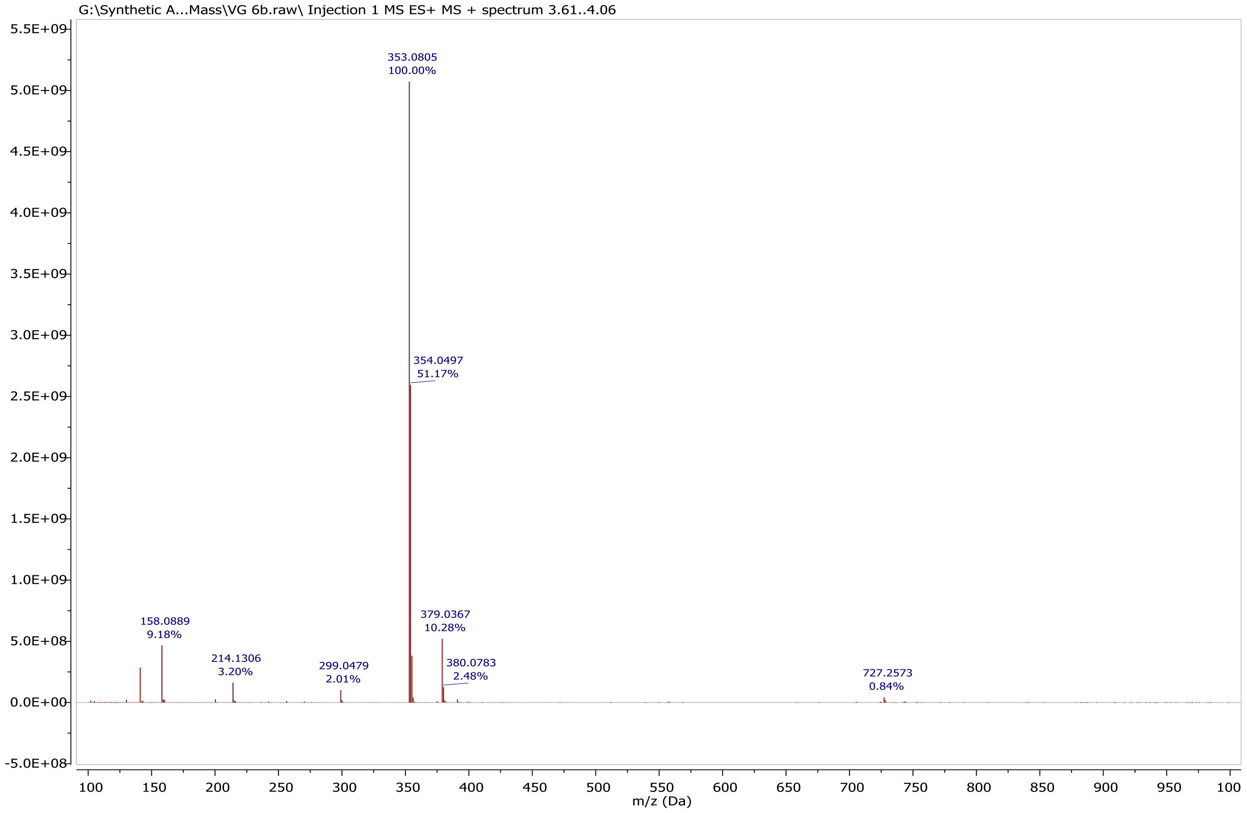

Figure S46. ESIMS spectrum of flavonoid (5b).

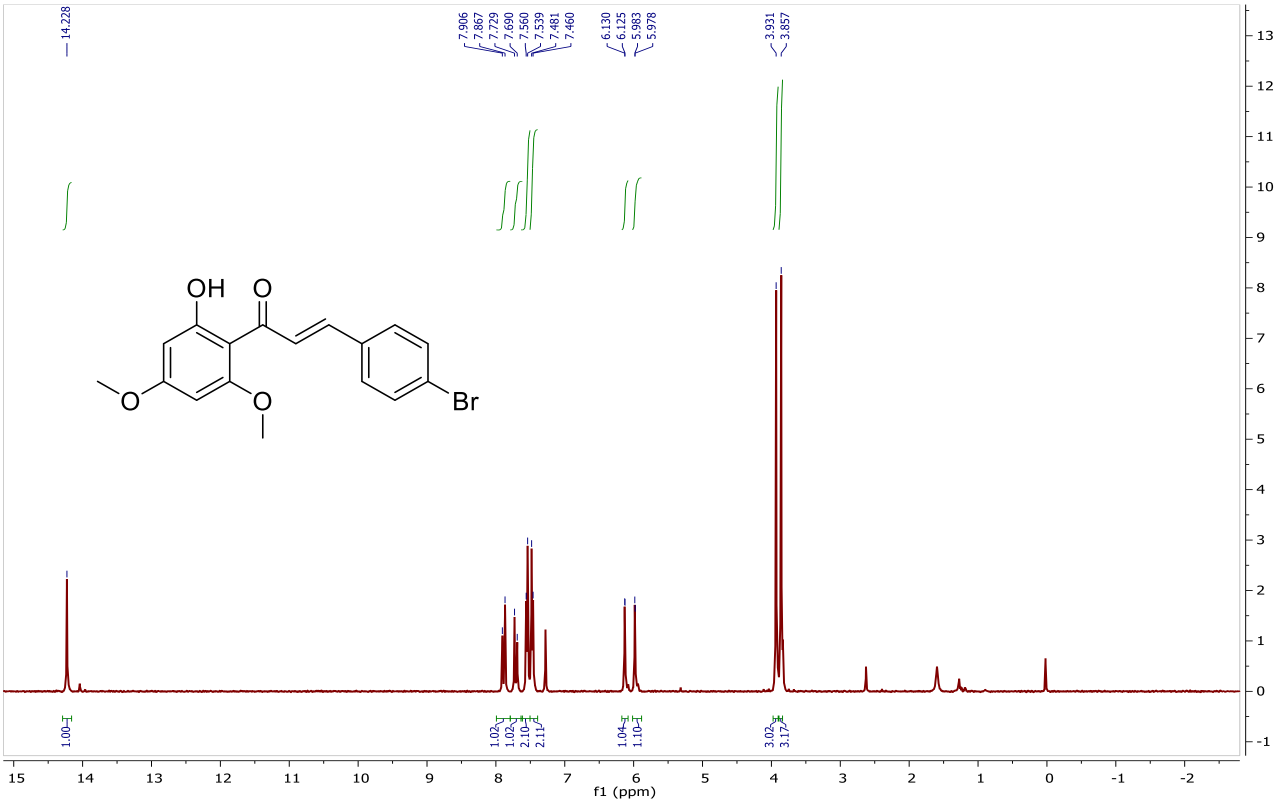

Figure S47. ^{1}H **NMR spectrum of chalcone (6a) in** $CDCl_3$**, 400 MHz**

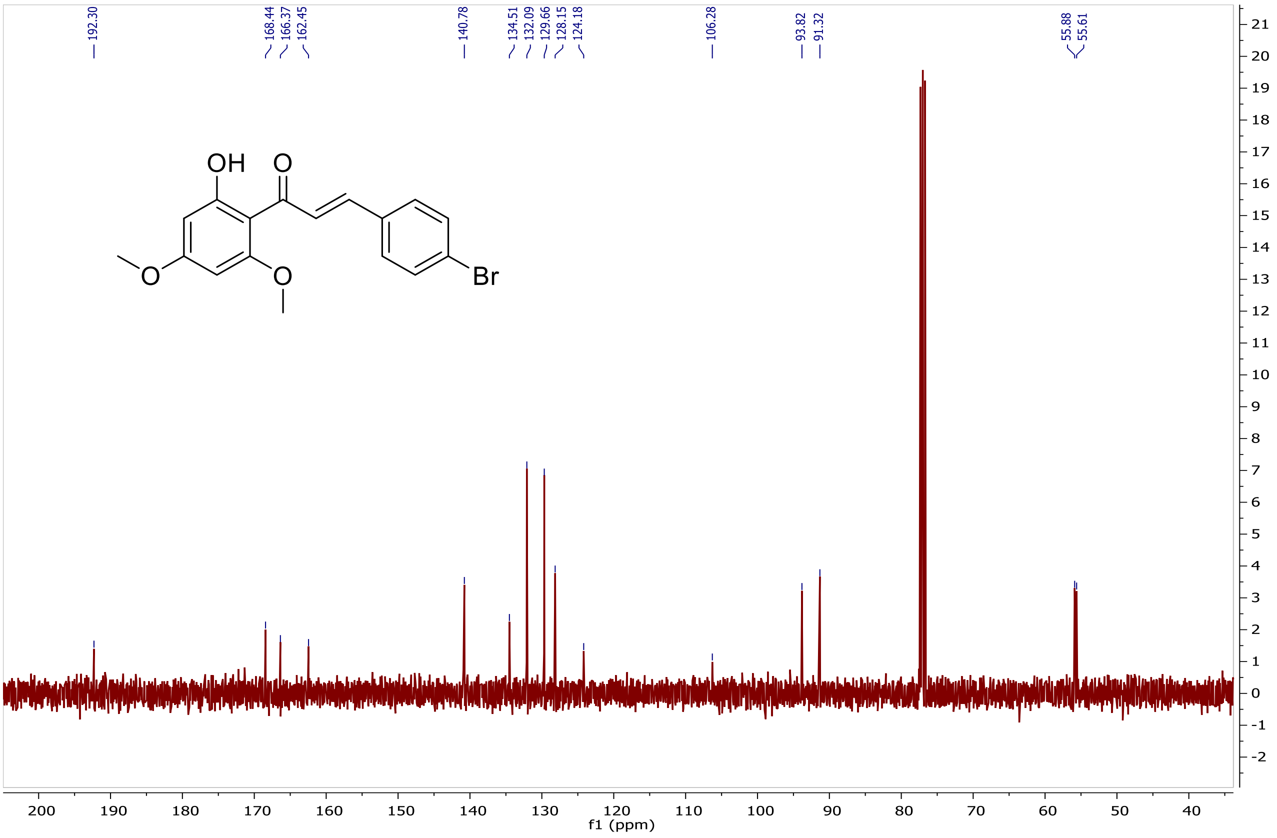

Figure S48. ^{13}C **NMR spectrum of chalcone (6a) in** $CDCl_3$**, 100 MHz**

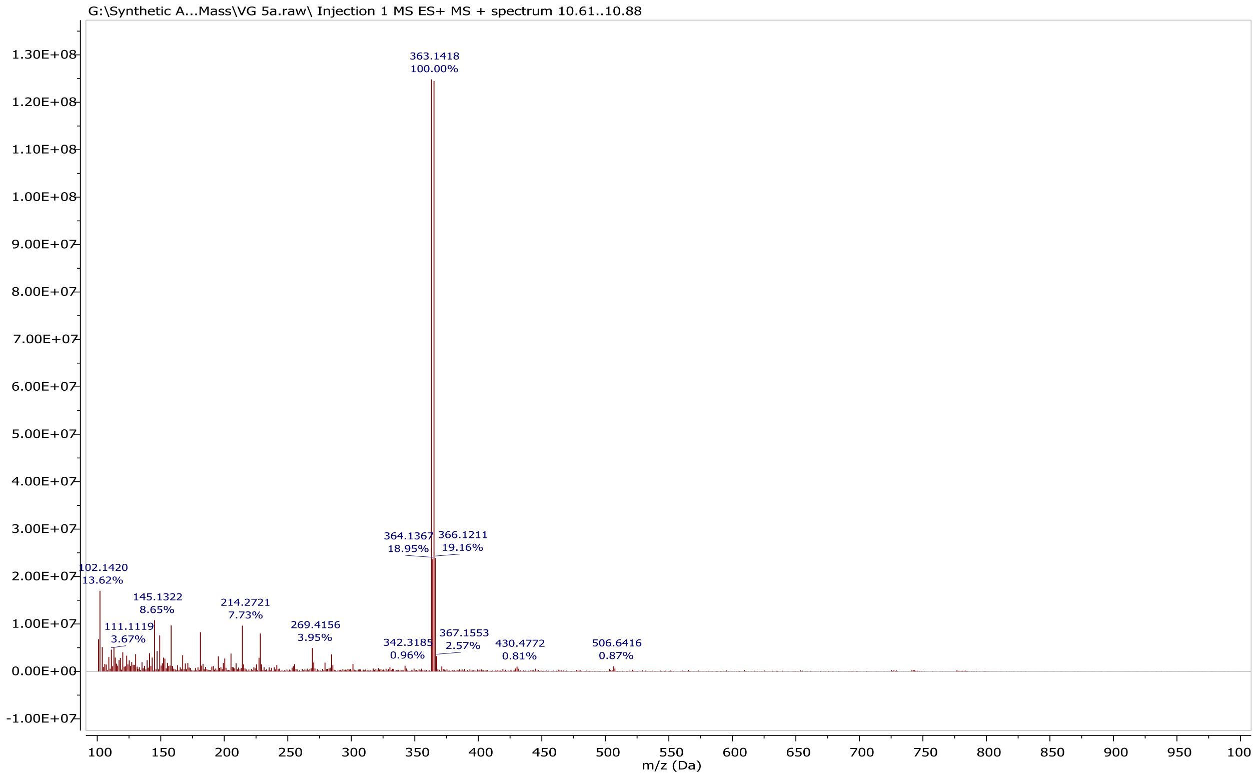

Figure S49. ESIMS spectrum of chalcone (6a).

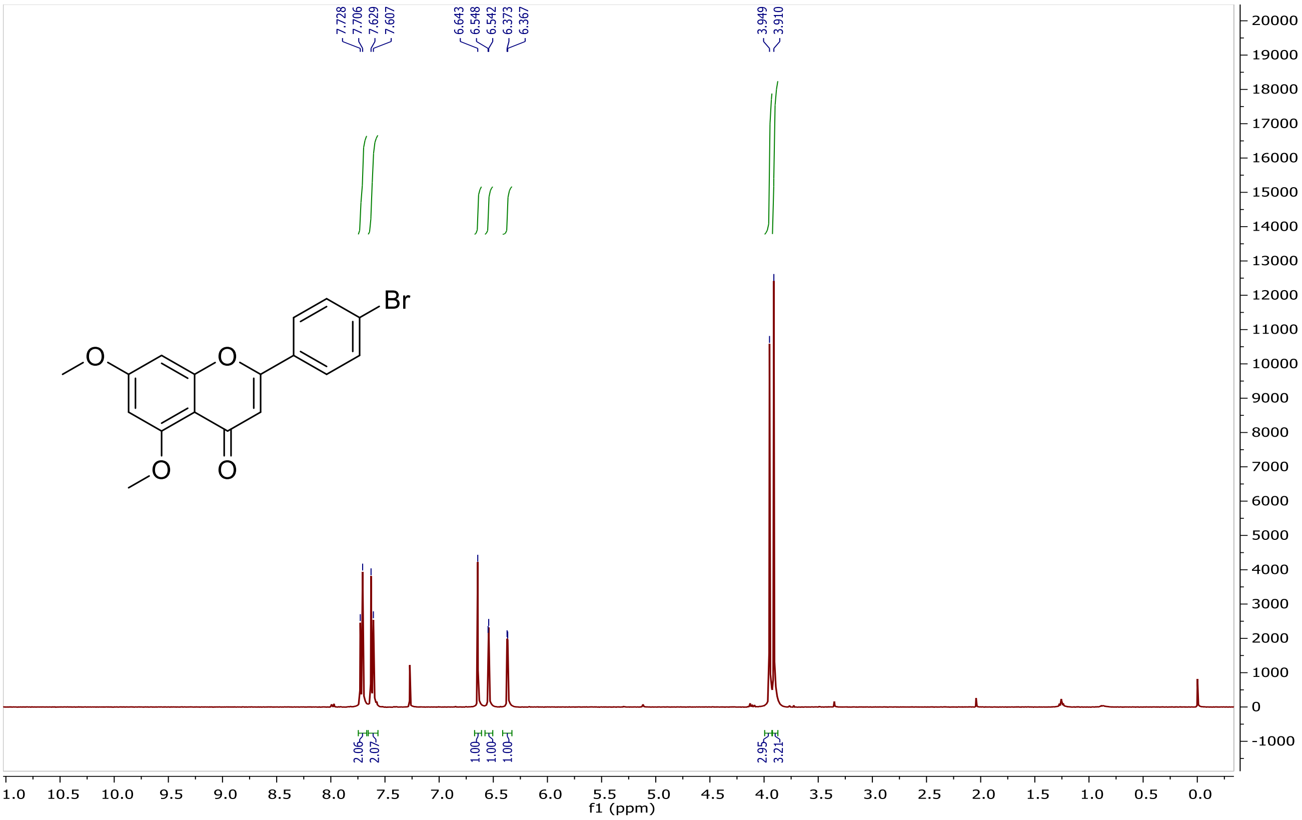

Figure S50. ^{1}H NMR spectrum of flavonoid (6b) in CDCl$_3$, 400 MHz

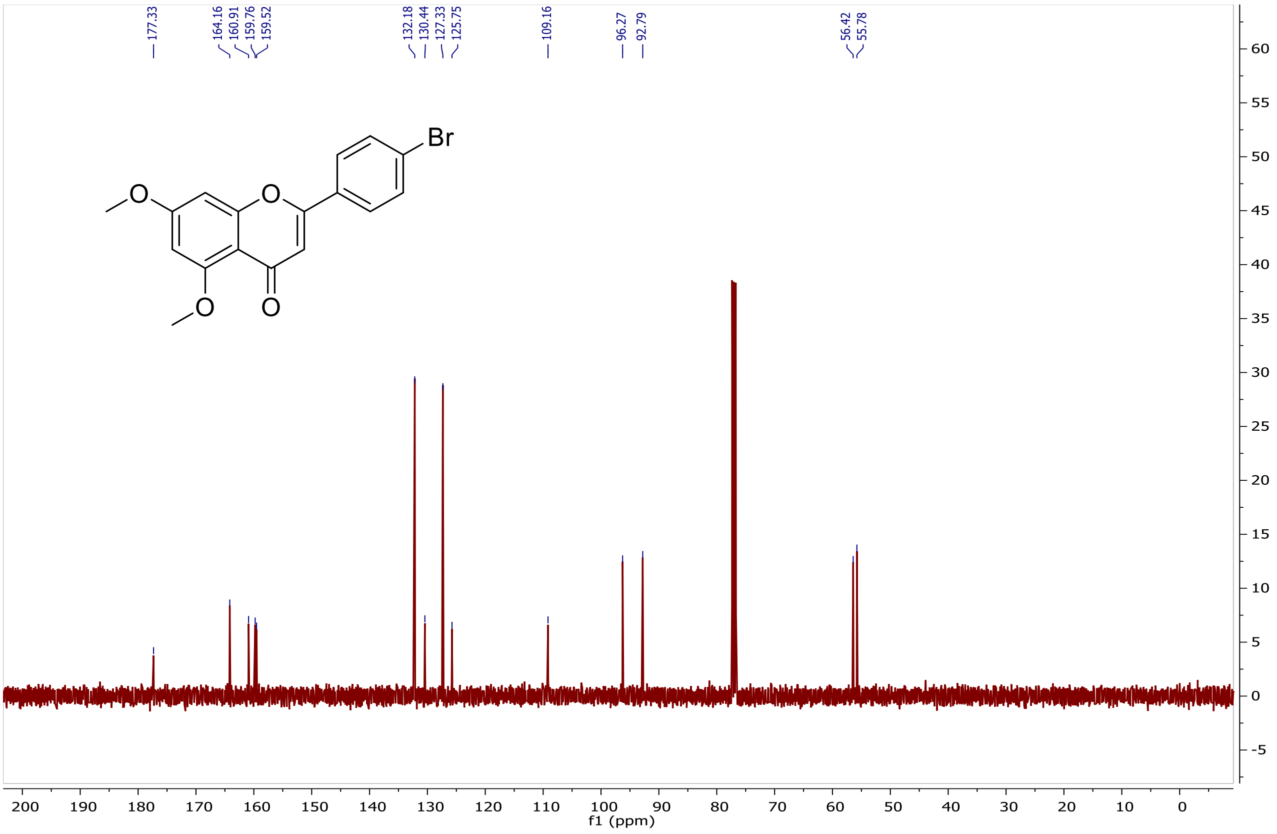

Figure S51. ^{13}**C NMR spectrum of flavonoid (6b) in CDCl**$_3$**, 100 MHz**

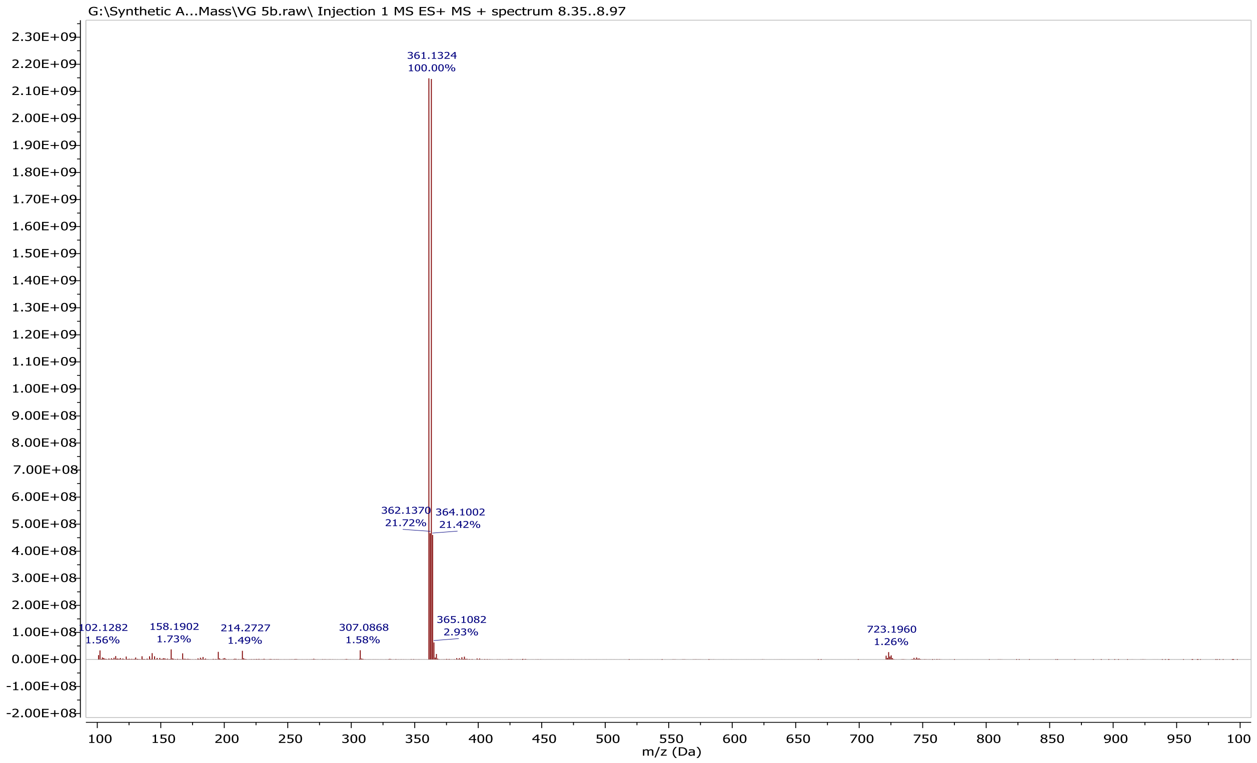

Figure S52. ESIMS spectrum of flavonoid (6b).

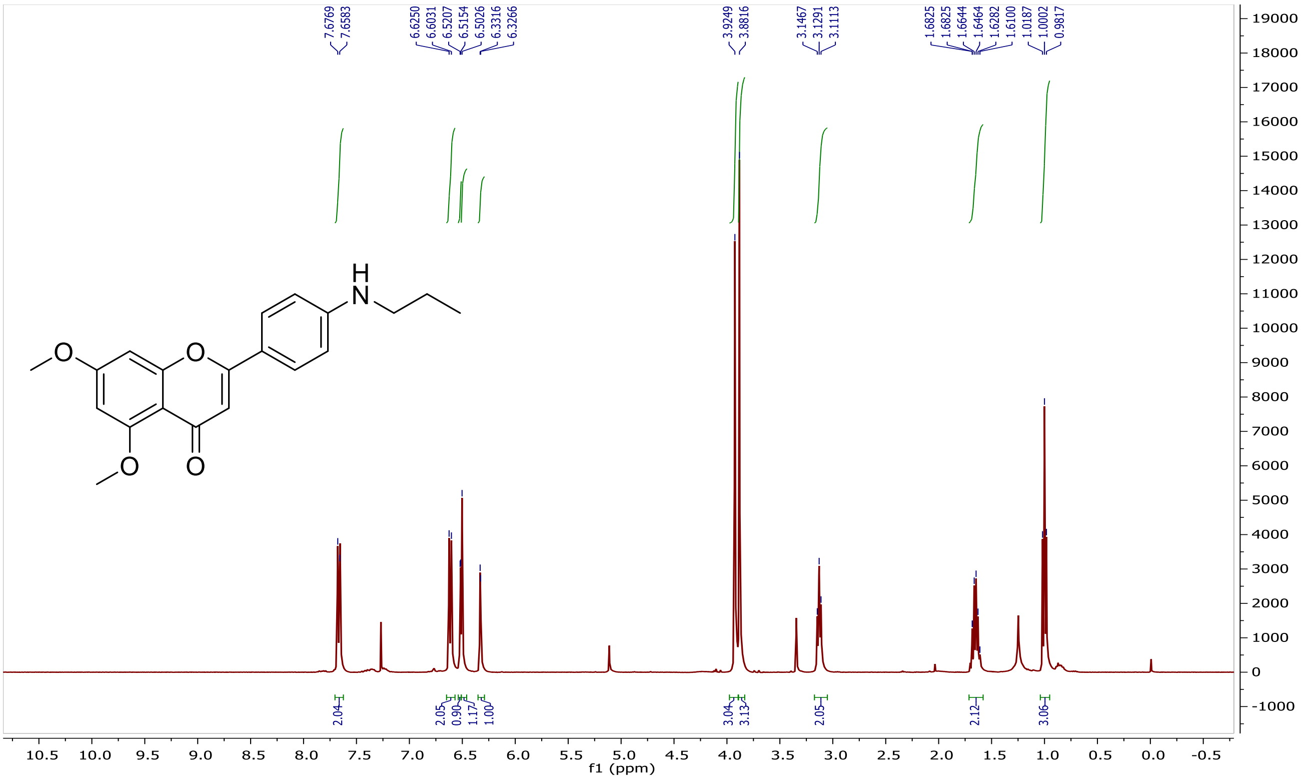

Figure S53. ^{1}H NMR spectrum of flavonoid (6c) in $CDCl_3$, 500 MHz

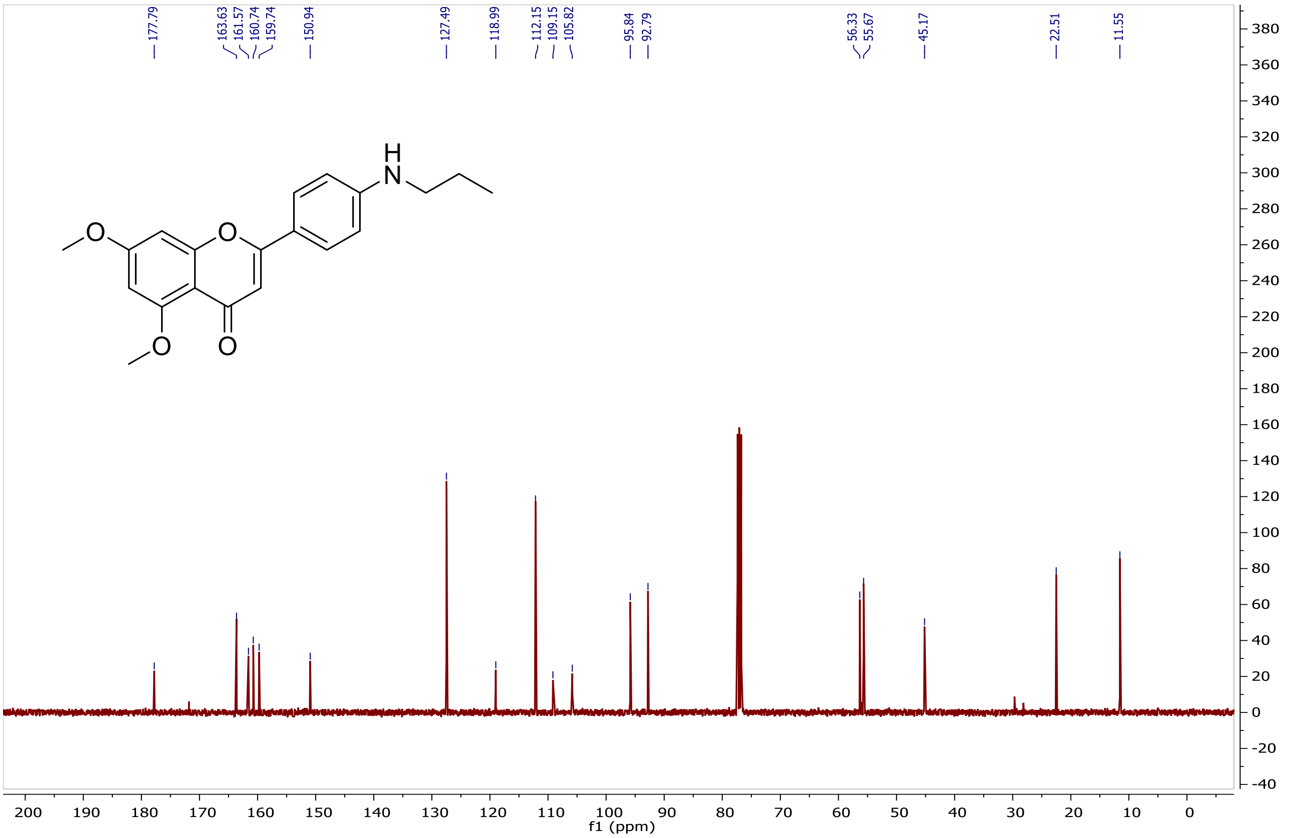

Figure S54. ^{13}C NMR spectrum of flavonoid (6c) in $CDCl_3$, 125 MHz

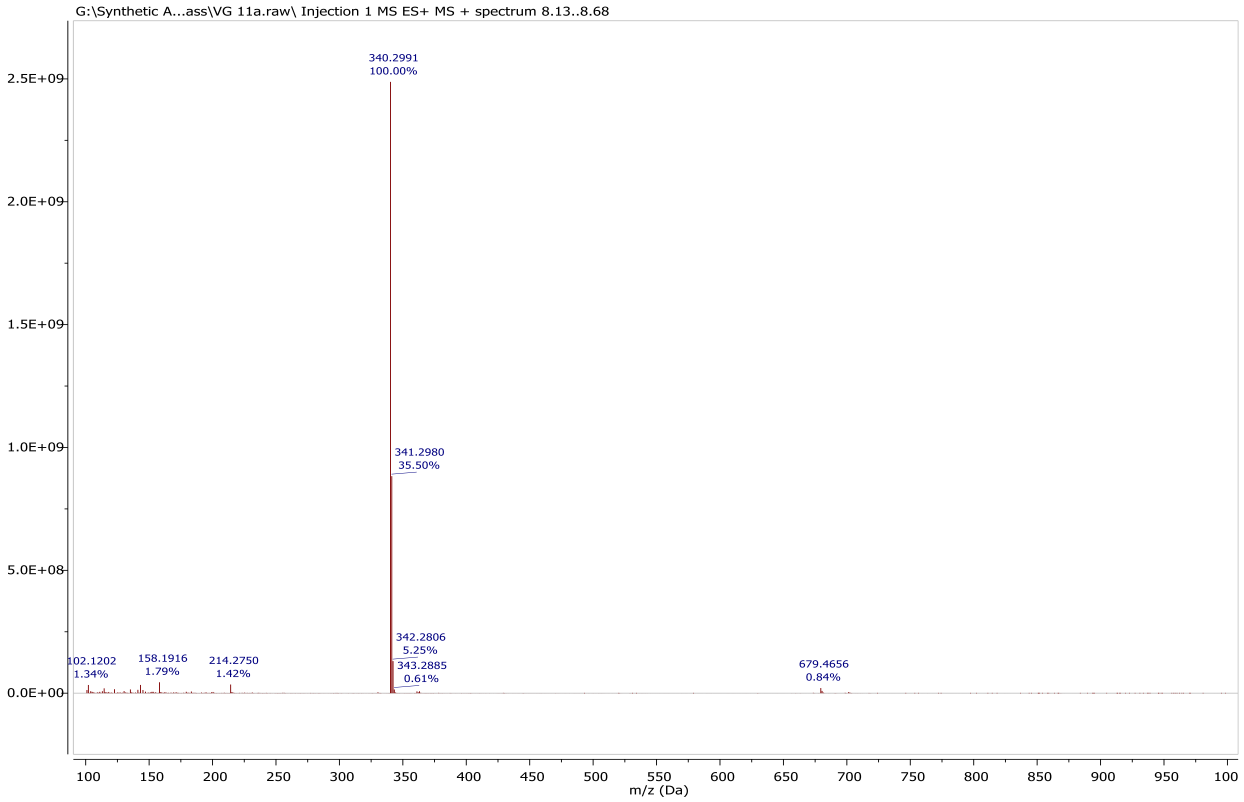

Figure S55. ESIMS spectrum of flavonoid (6c).

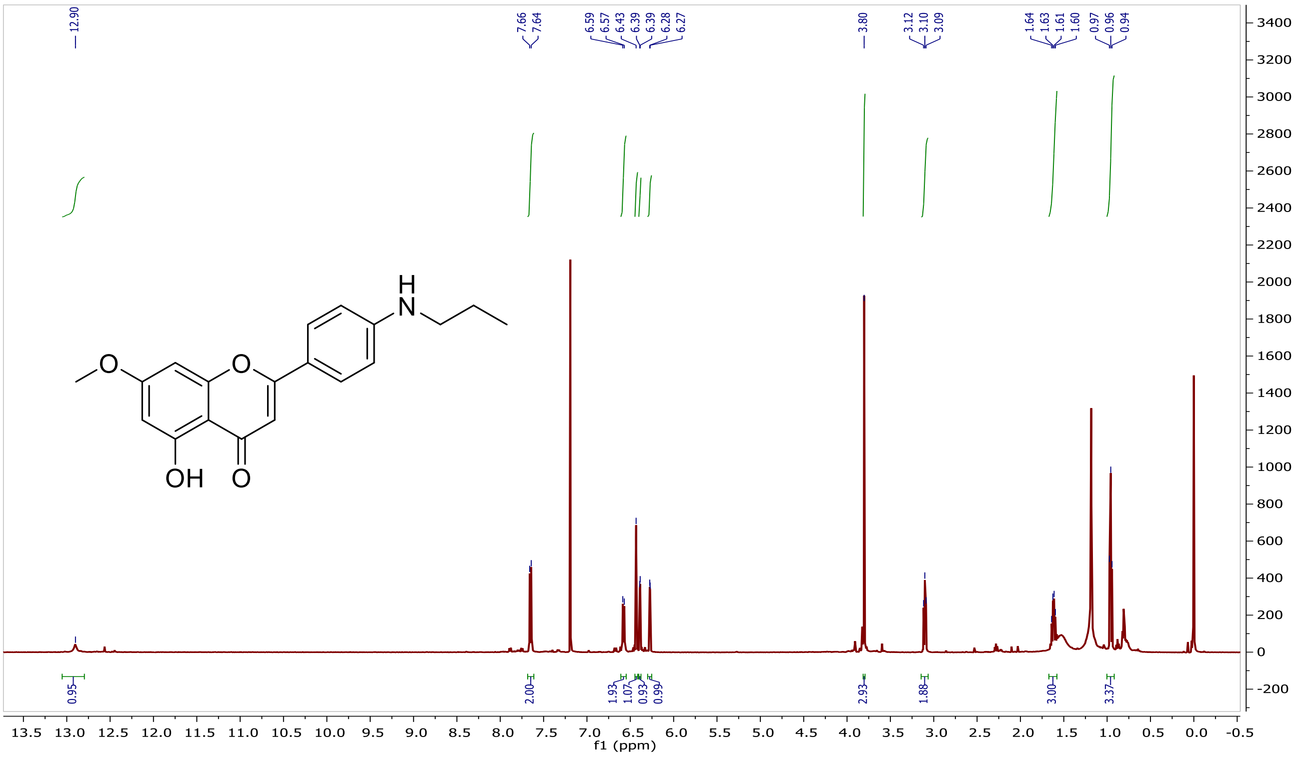

Figure S56. ^{1}H NMR spectrum of flavonoid (6d) in $CDCl_3$, 500 MHz

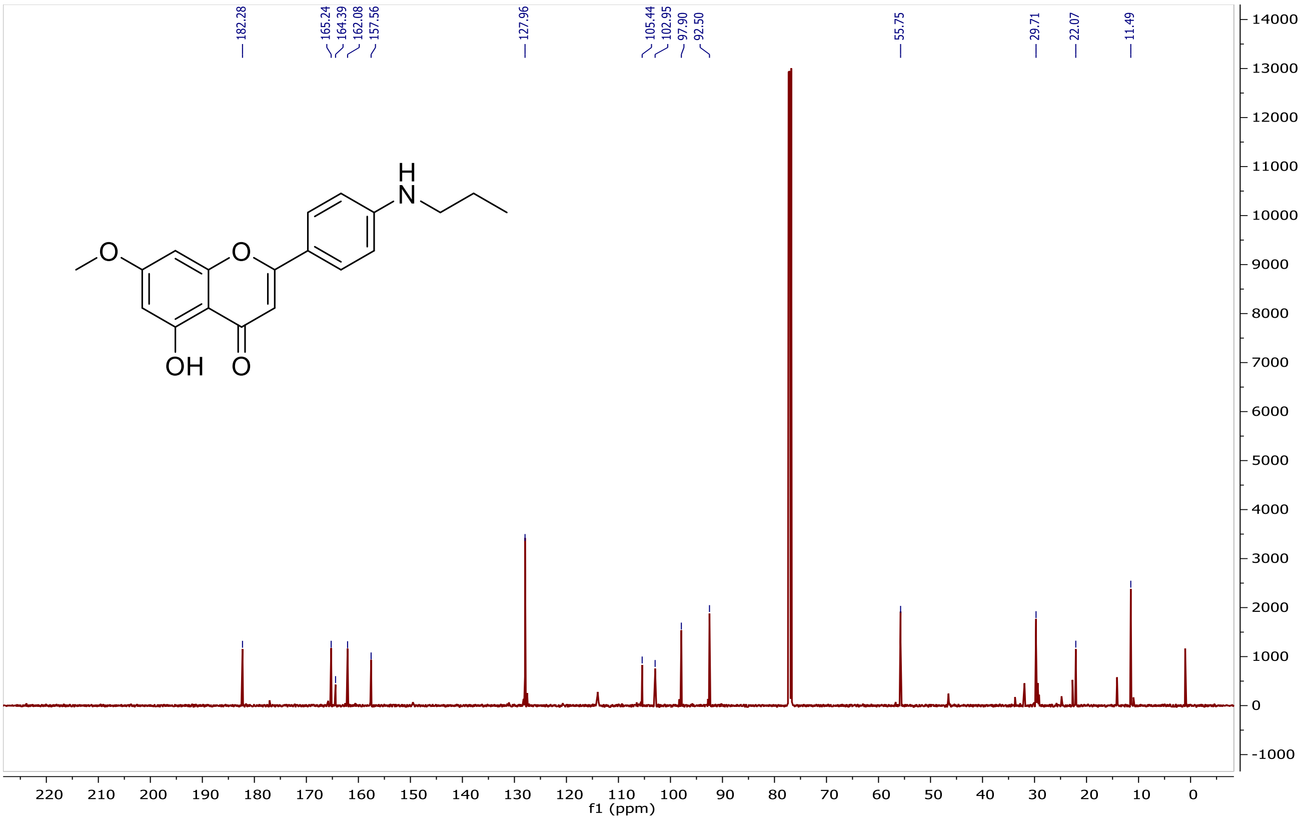

Figure S57. ^{13}**C NMR spectrum of flavonoid (6d) in CDCl**$_3$**, 125 MHz**

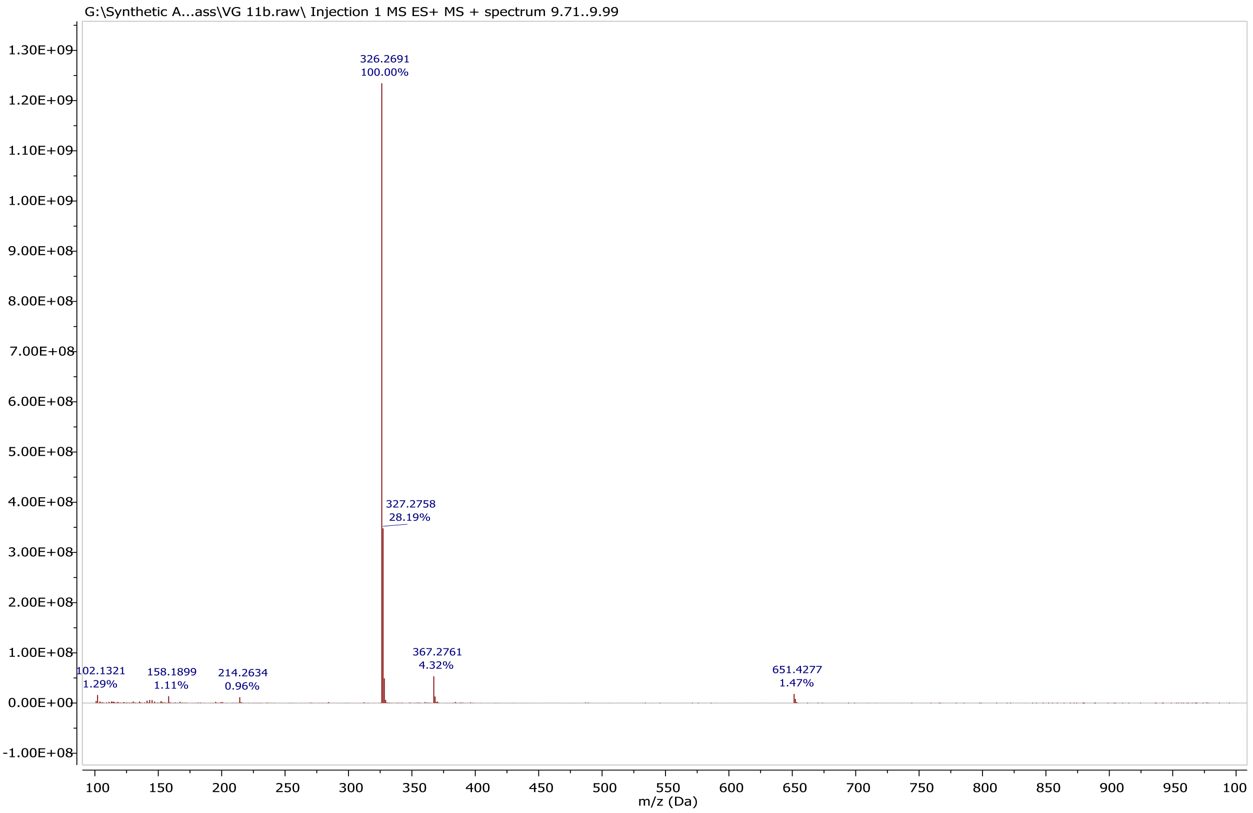

Figure S58. ESIMS spectrum of flavonoid (6d).

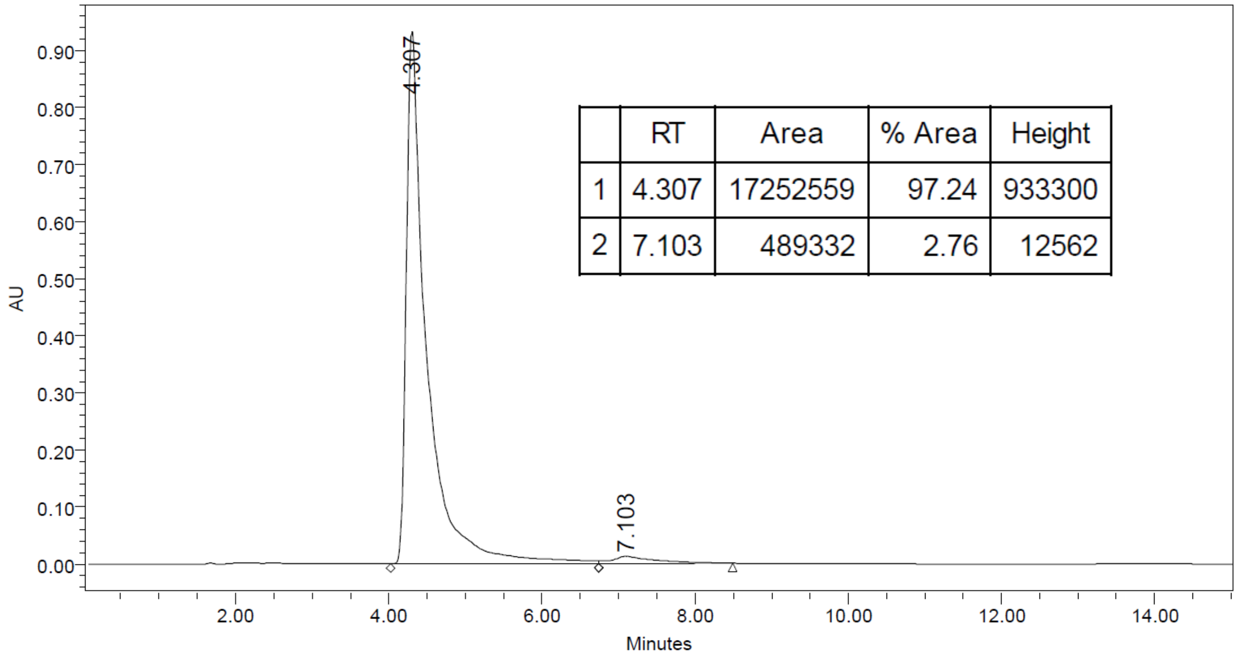

Figure S59. HPLC analysis of flavonoid (6d).

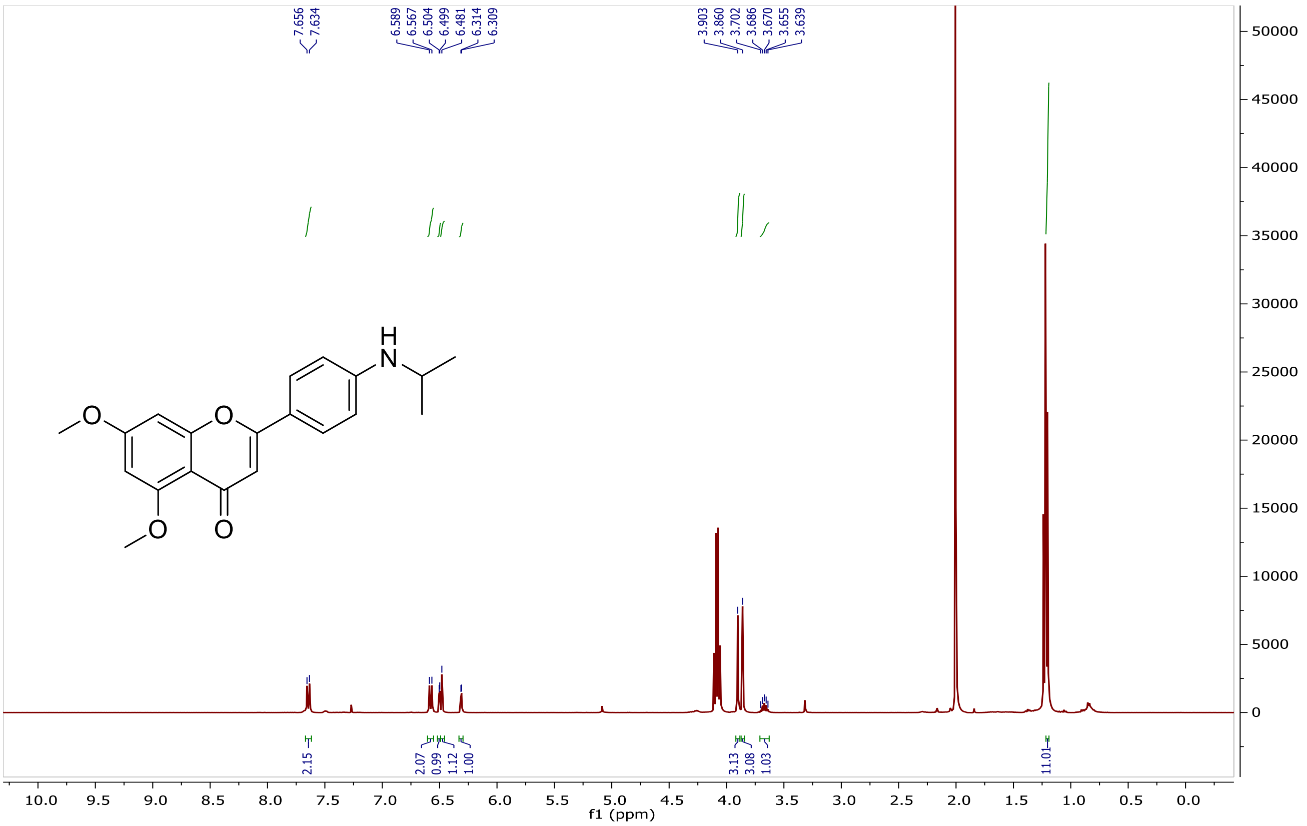

Figure S60. ^{1}H NMR spectrum of flavonoid (7c) in $CDCl_3$, 400 MHz

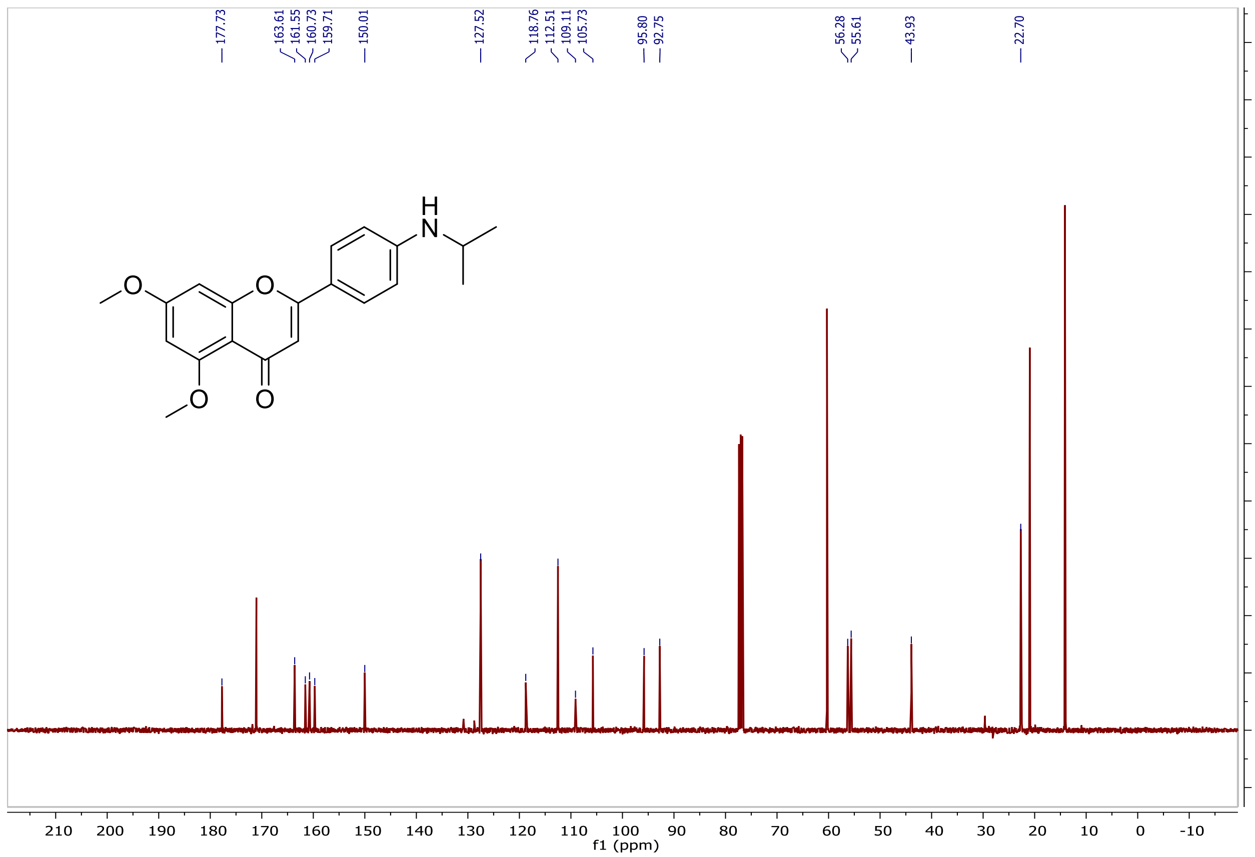

Figure S61. ^{13}C **NMR spectrum of flavonoid (7c) in** $CDCl_3$**, 100 MHz**

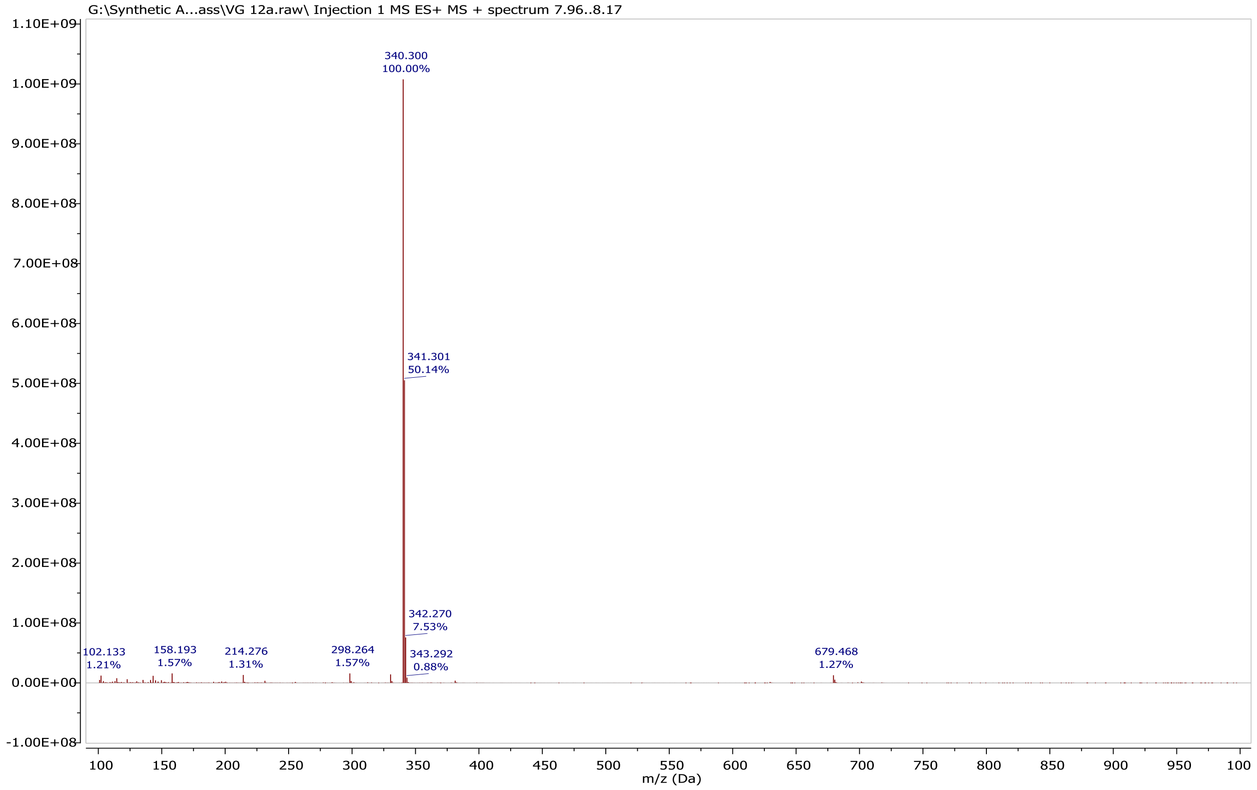

Figure S62. ESIMS spectrum of flavonoid (7c).

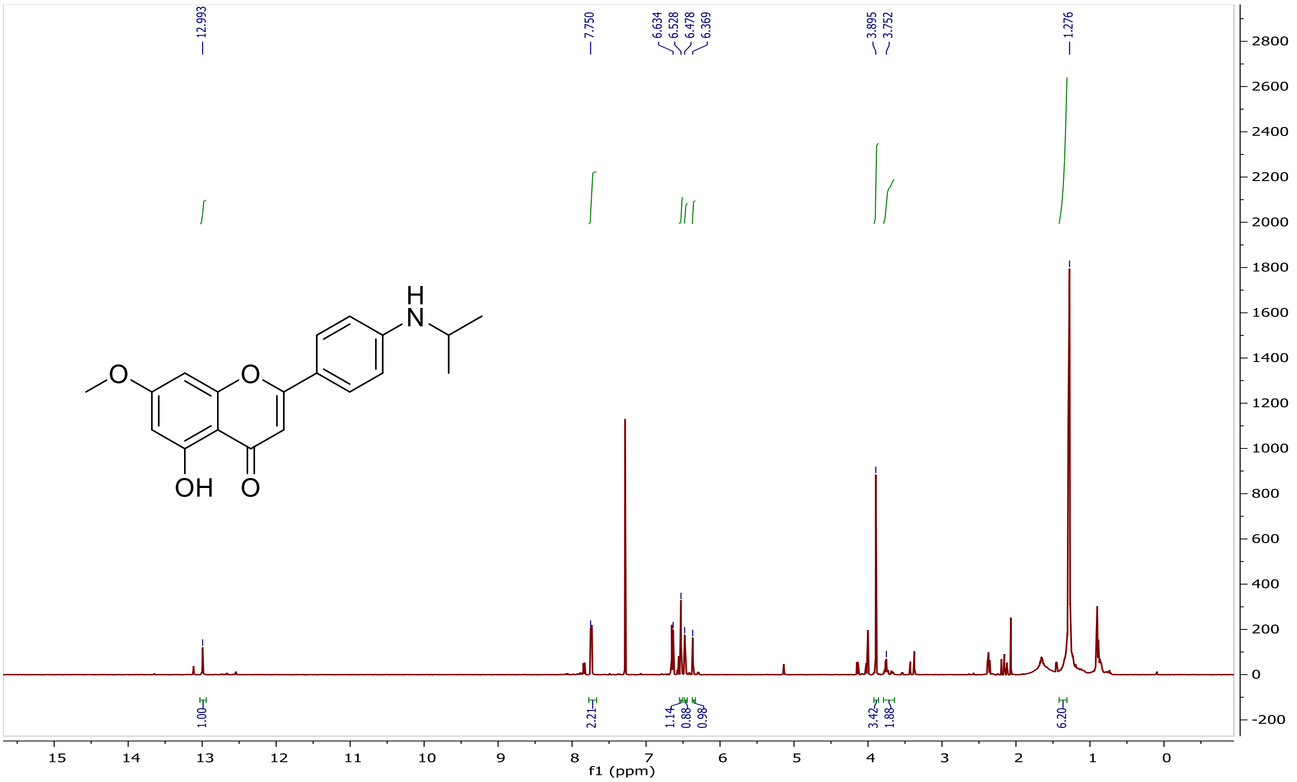

Figure S63. ^{1}H NMR spectrum of flavonoid (7d) in CDCl₃, 500 MHz

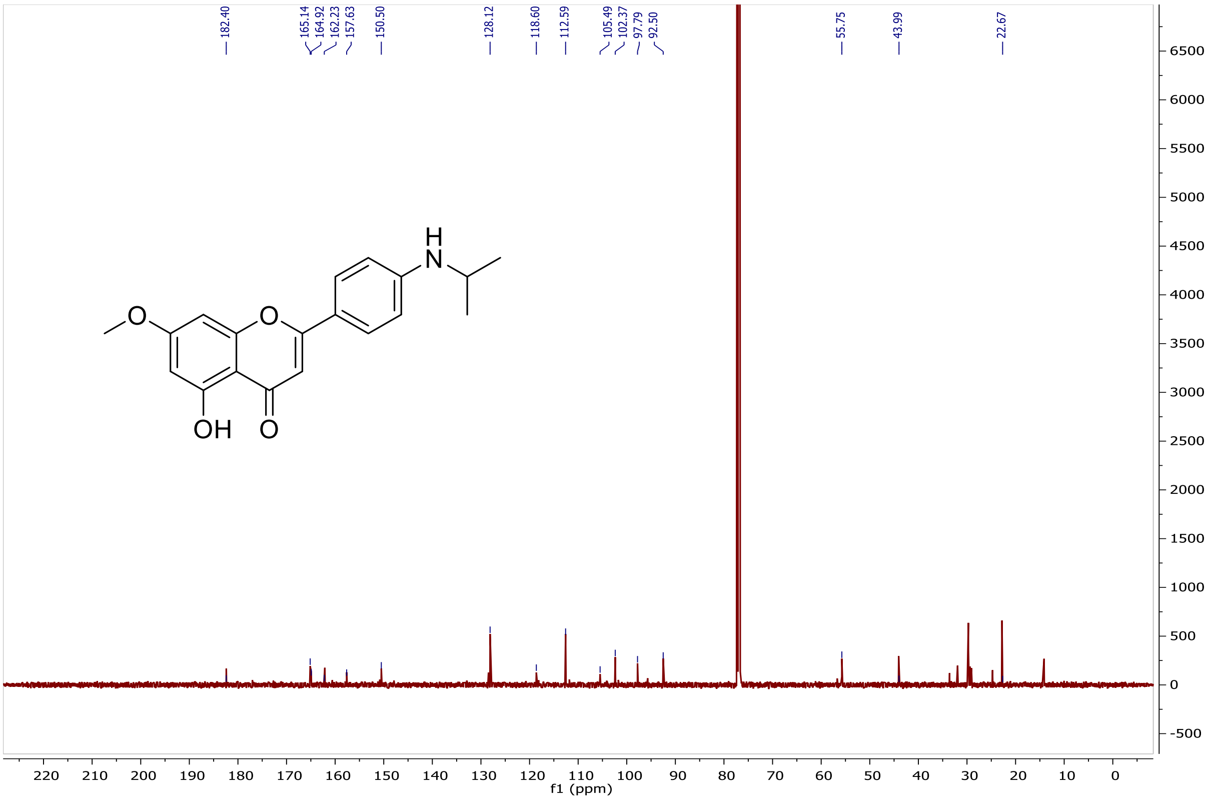

Figure S64. ^{1}H NMR spectrum of flavonoid (7d) in CDCl₃, 125 MHz

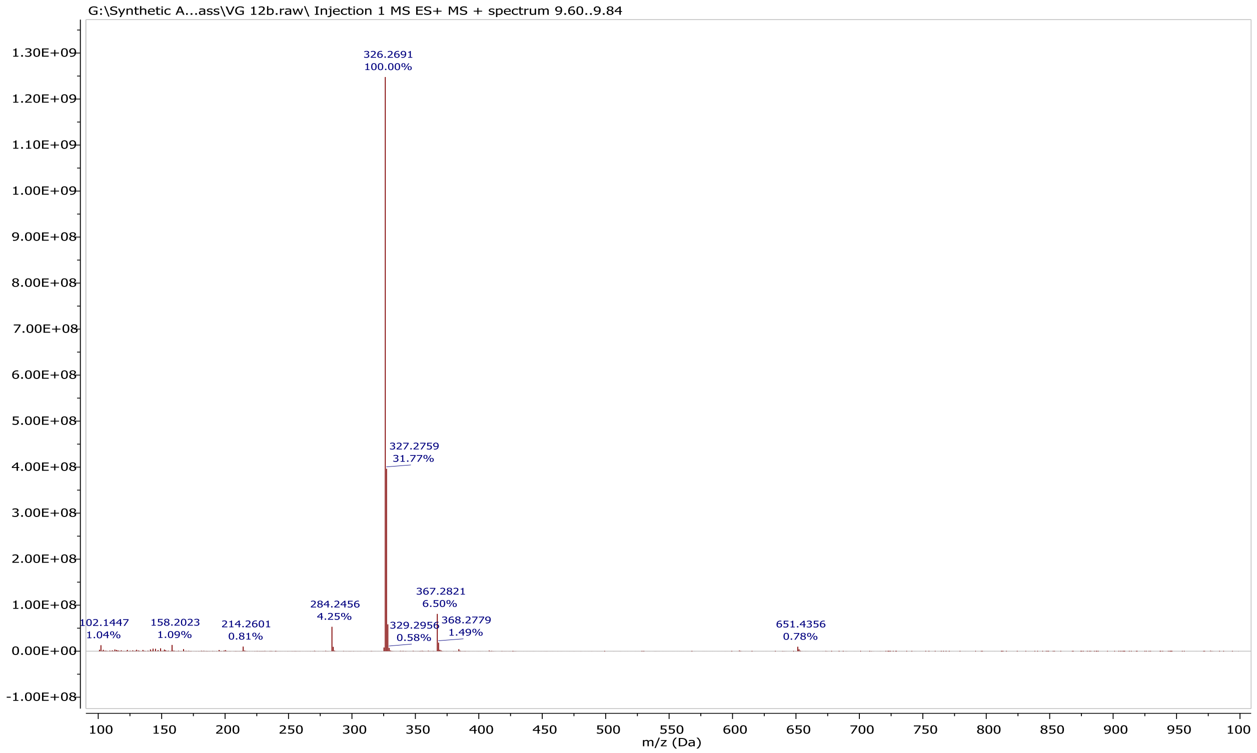

Figure S65. ESIMS spectrum of flavonoid (7d).

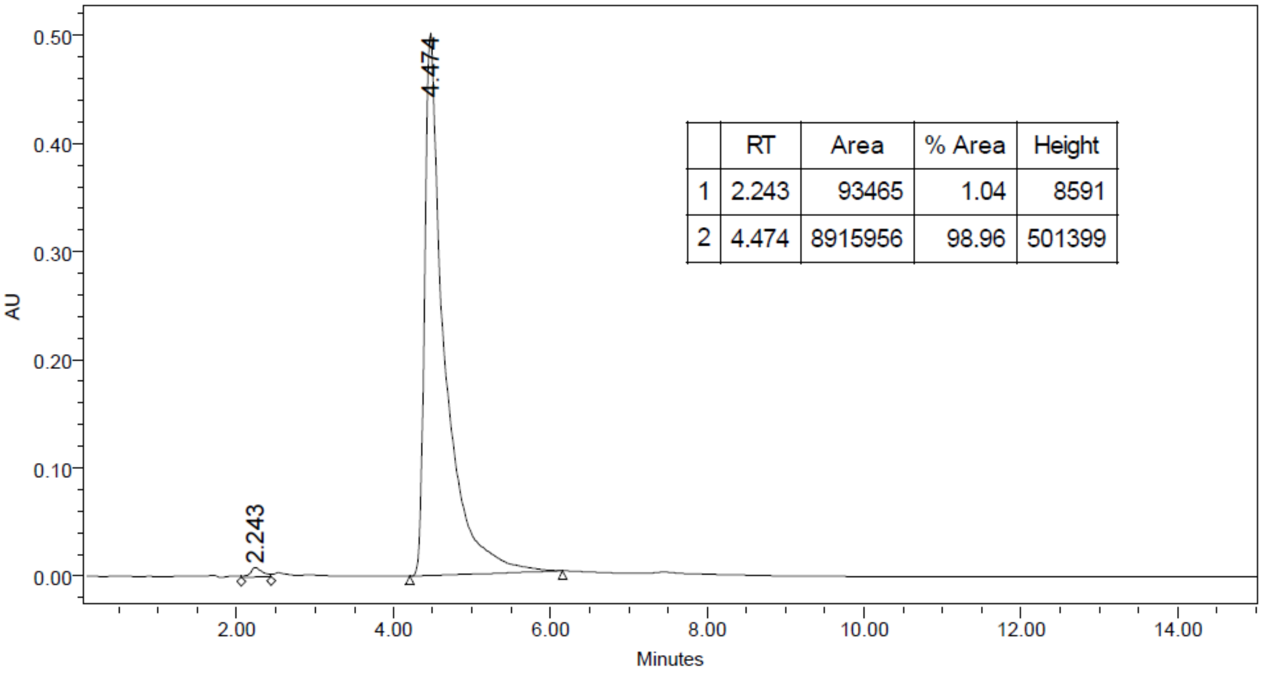

	RT	Area	% Area	Height
1	2.243	93465	1.04	8591
2	4.474	8915956	98.96	501399

Figure S66. HPLC analysis of flavonoid (7d).

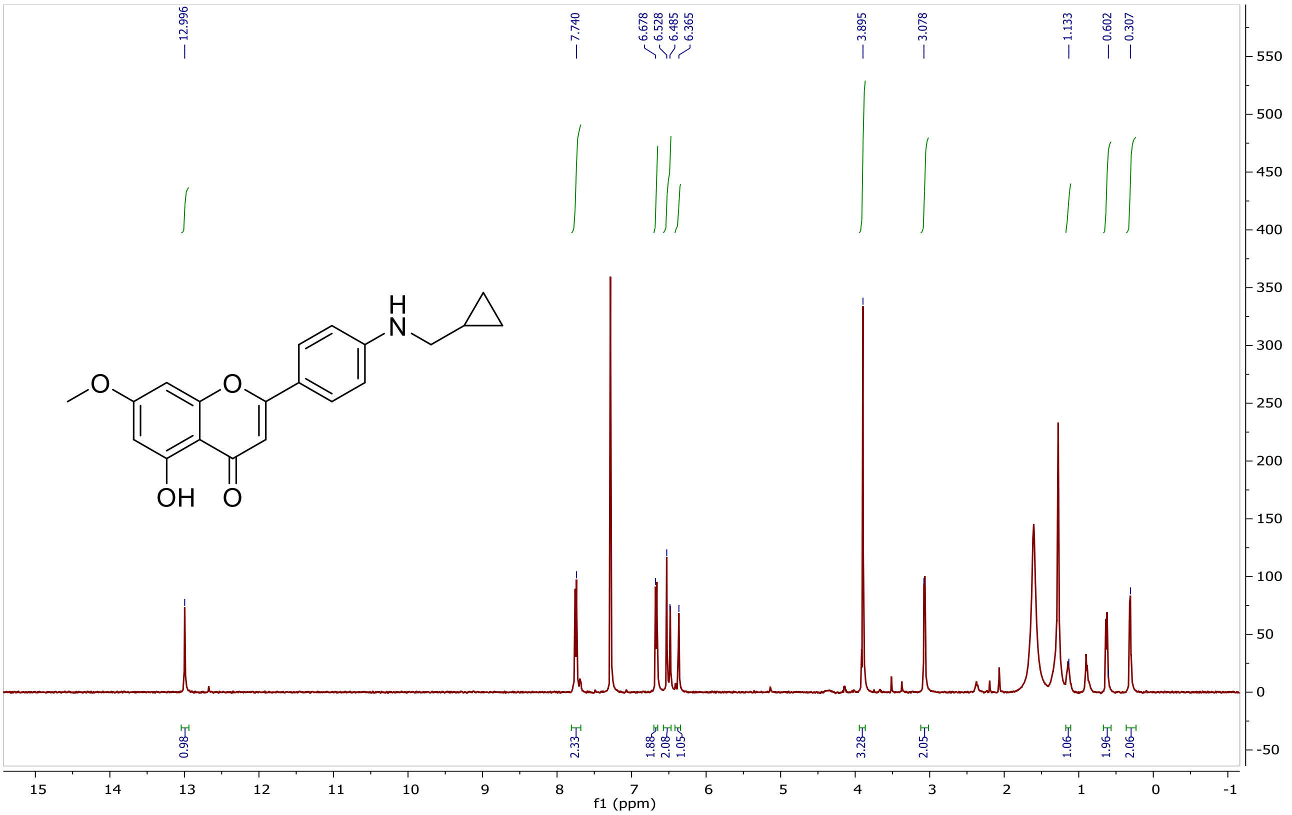

Figure S67. ^{1}H NMR spectrum of flavonoid (8d) in CDCl$_3$, 500 MHz

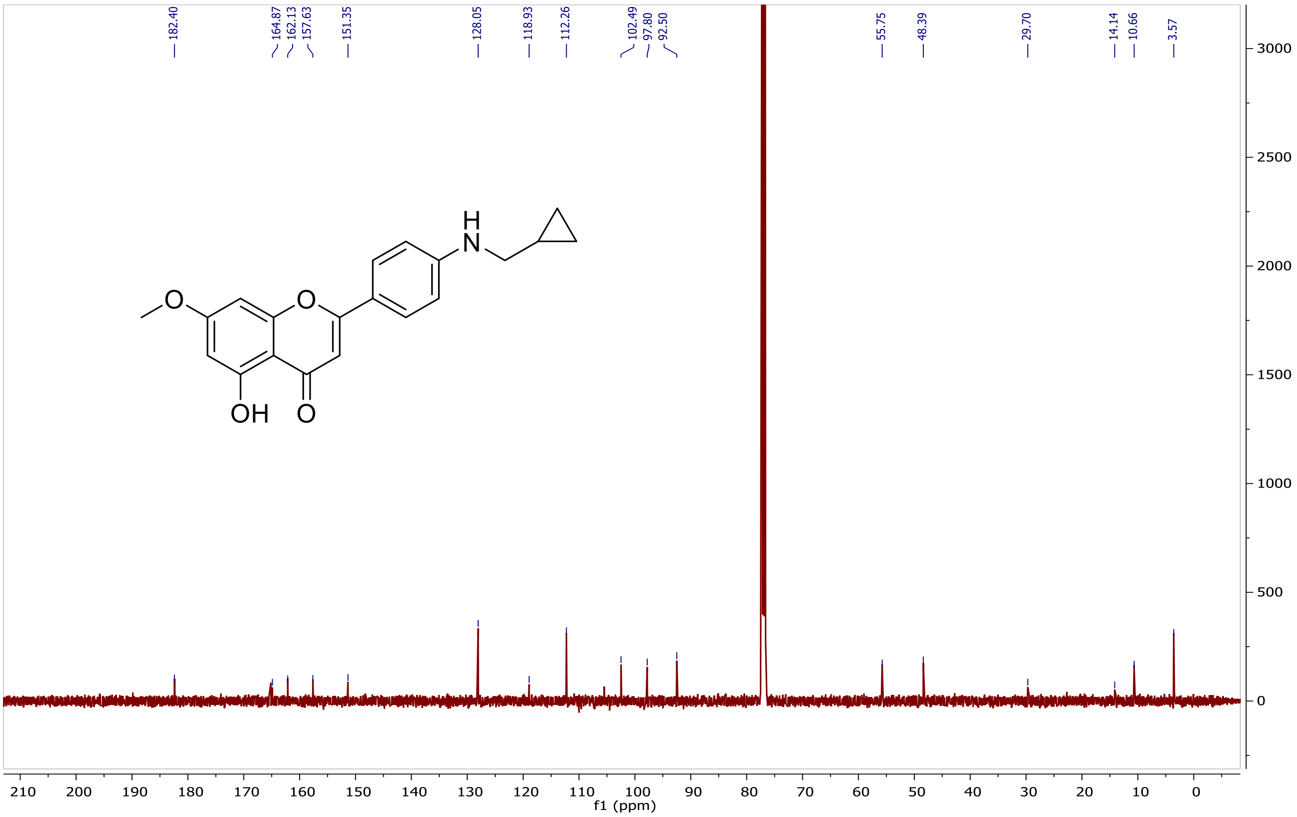

Figure S68. ^{1}H NMR spectrum of flavonoid (8d) in CDCl$_3$, 125 MHz

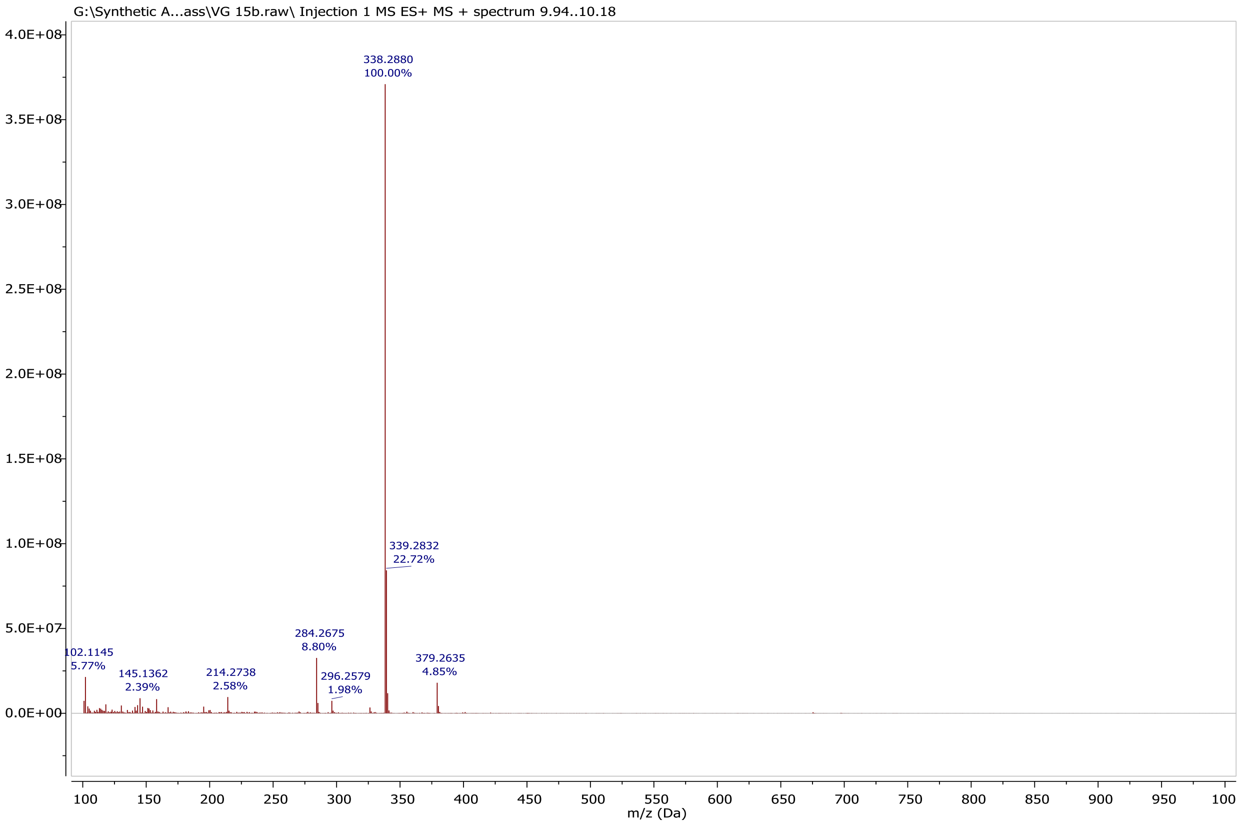

Figure S69. ESIMS spectrum of flavonoid (8d)

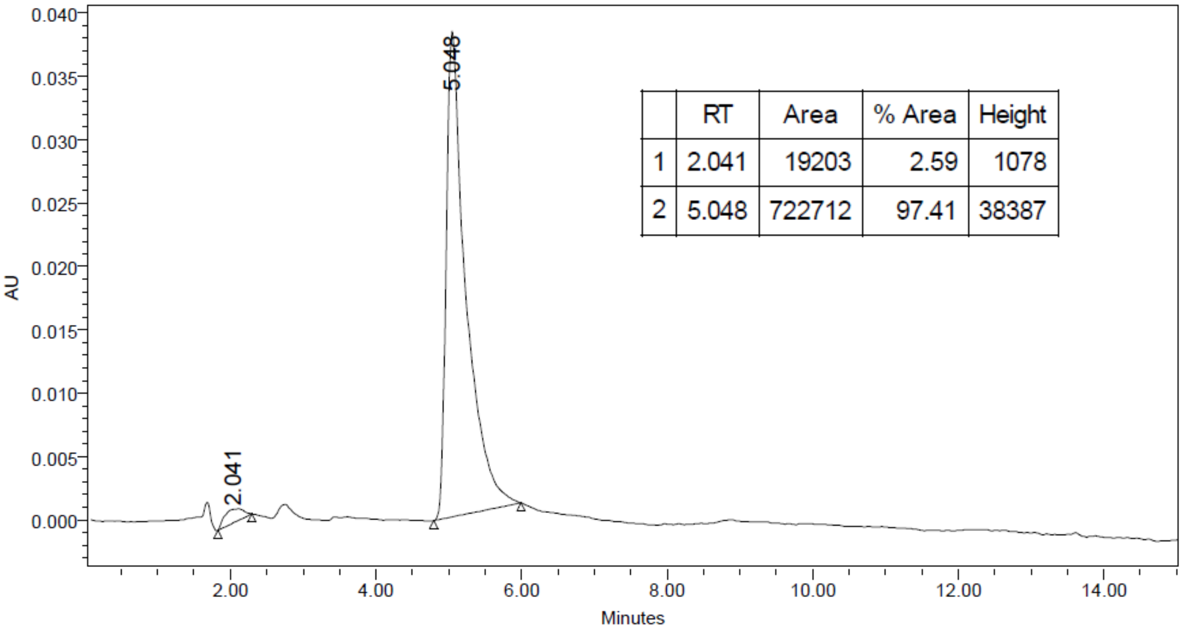

	RT	Area	% Area	Height
1	2.041	19203	2.59	1078
2	5.048	722712	97.41	38387

Figure S70. HPLC analysis of flavonoid (8d).

Computational Interactions of Acacetin and MAO-A and MAO-B

To understand the binding behavior of acacetin in MAO-A and MAO-B binding sites, a long molecular dynamics (MD) simulation study (200 ns) was conducted. The interaction profile and the average changes in the heavy atom displacement of acacetin (Figure S71) were monitored over the course of the MD simulations time with respect to the original frame using the following equation:

$$\boldsymbol{RMSD_x} = \sqrt{\frac{1}{N}\sum_{i=1}^{N}\left(r_i'(t_x) - r_i(t_{ref})\right)^2}$$

Frame x is recorded at time t_x; $RMSD_x$ is the root mean square deviation of selected atoms in frame x; N is the number of atoms; t_{ref} is the reference time; r_i' is the position of the selected atom (i) after aligning on the reference frame.

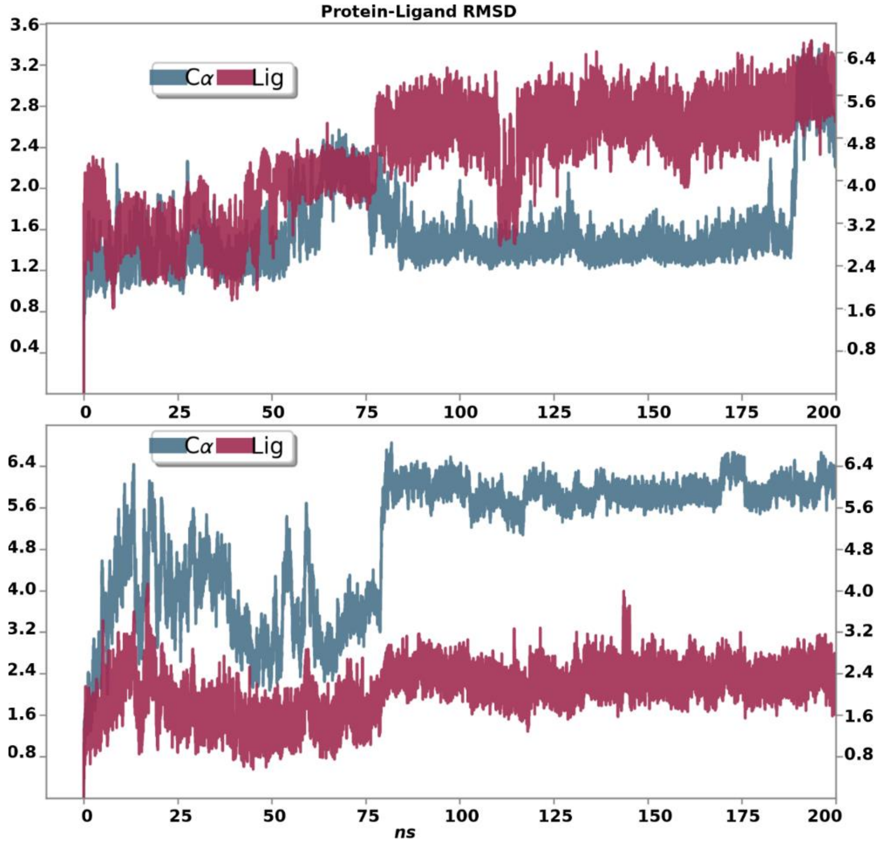

Figure S71. Protein-ligand RMSD. Top: RMSD of MAO-B. Bottom: RMSD of MAO-A.

Acacetin displayed stable binding in MAO-B with an average RMSD of 2.8 Å for the protein backbone and 6.0 Å for the ligand (Figure S71, top), while it has an average RMSD of 6.4 Å for MAO-A backbone and 2.4 Å for the ligand (Figure S71, bottom). Several prominent interactions were traced and averaged over the MD simulations. With MAO-A (Figure S72), there are two strong and highly abundant (over 95% of the MD simulations time) hydrogen bonds between the flavone hydroxyl group and Asn 181, and Tyr 444. These two hydrogen bonds are in the negative free energy

region of the water density calculations. Interrupting these interactions with bulkier and hydrophobic substituents would decrease binding to MAO-A. At the same position of MAO-B, there is one strong hydrogen bond with Thr 174 and π-π stacking with Tyr 398 (Figure S73). Modifying acacetin to have small hydrophobic groups at this position (R_1) should not alter MAO-B activity. In general, acacetin has stronger hydrogen bonds and more polar contacts with MAO-A, and more hydrophobic contacts with MAO-B (Figures S72-73).

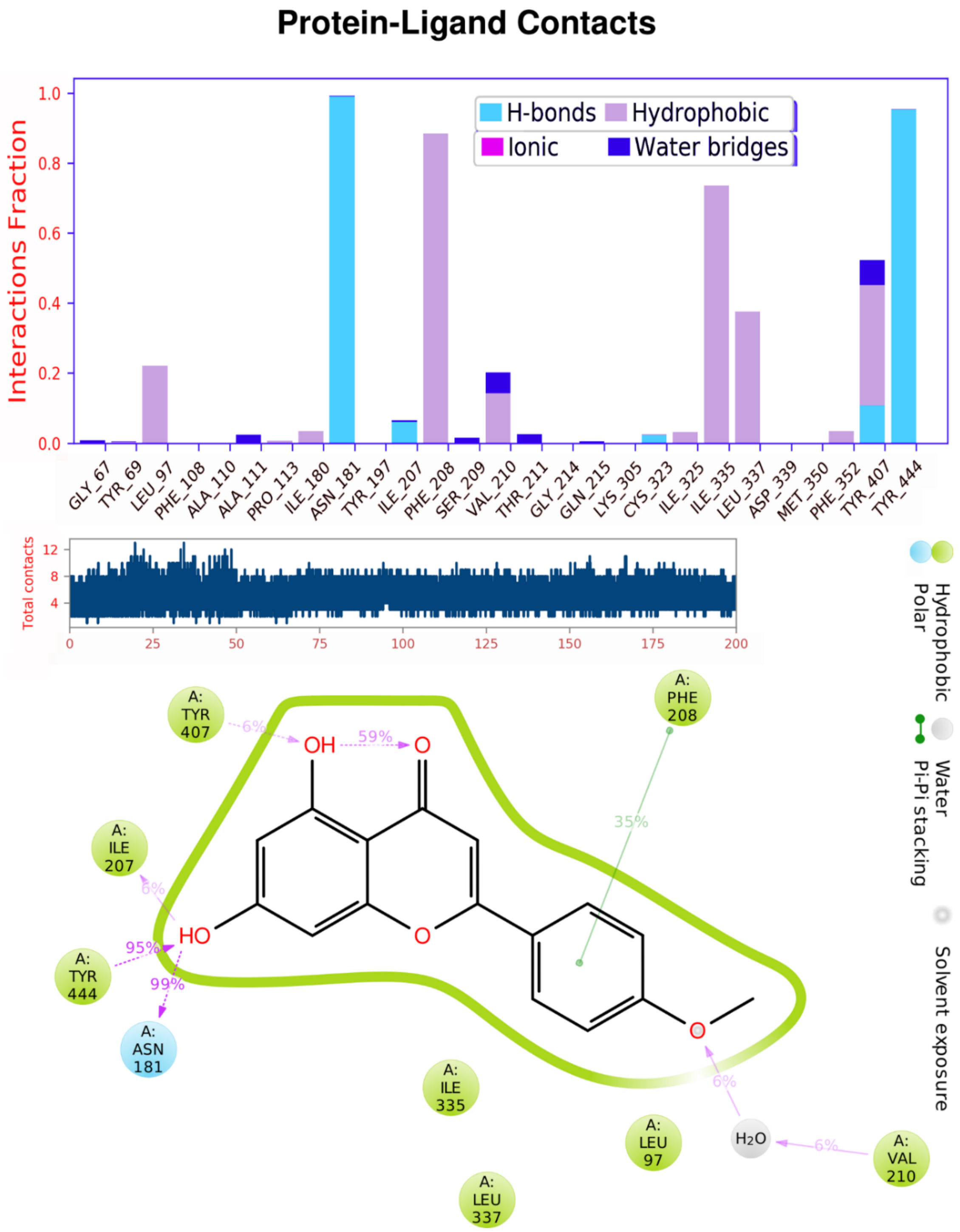

Figure S72 Interaction profile of acacetin in MAO-A.

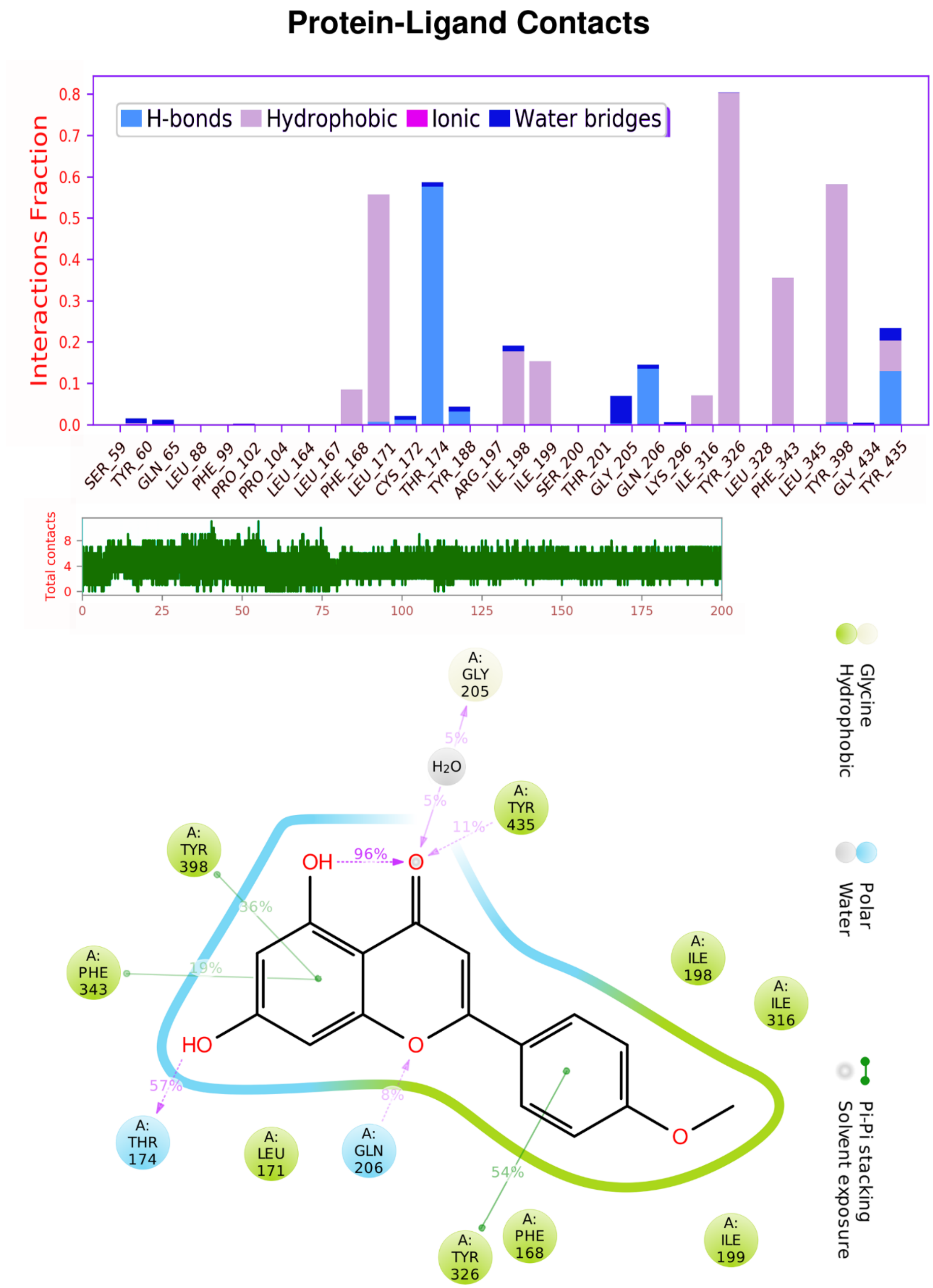

Figure S73. Interaction profile of acacetin in MAO-B.

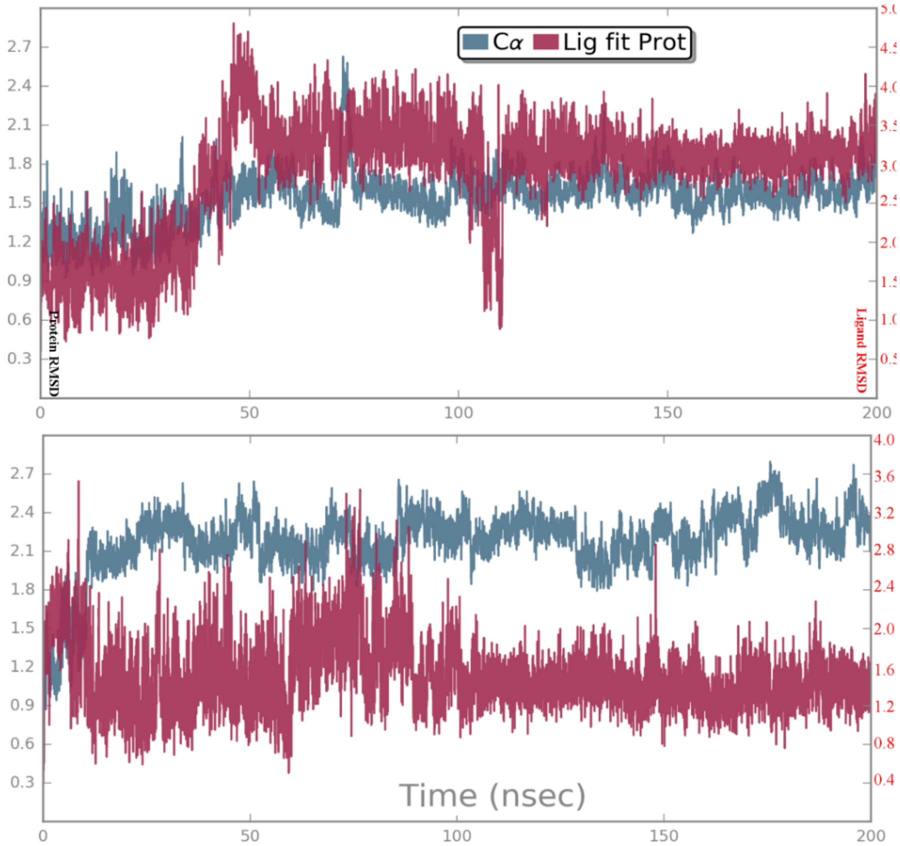

Figure S74. Protein and ligand RMSD. Top: RMSD of pose 1. Bottom: RMSD of pose 2 for acacetin 7-*O*-methyl ether.

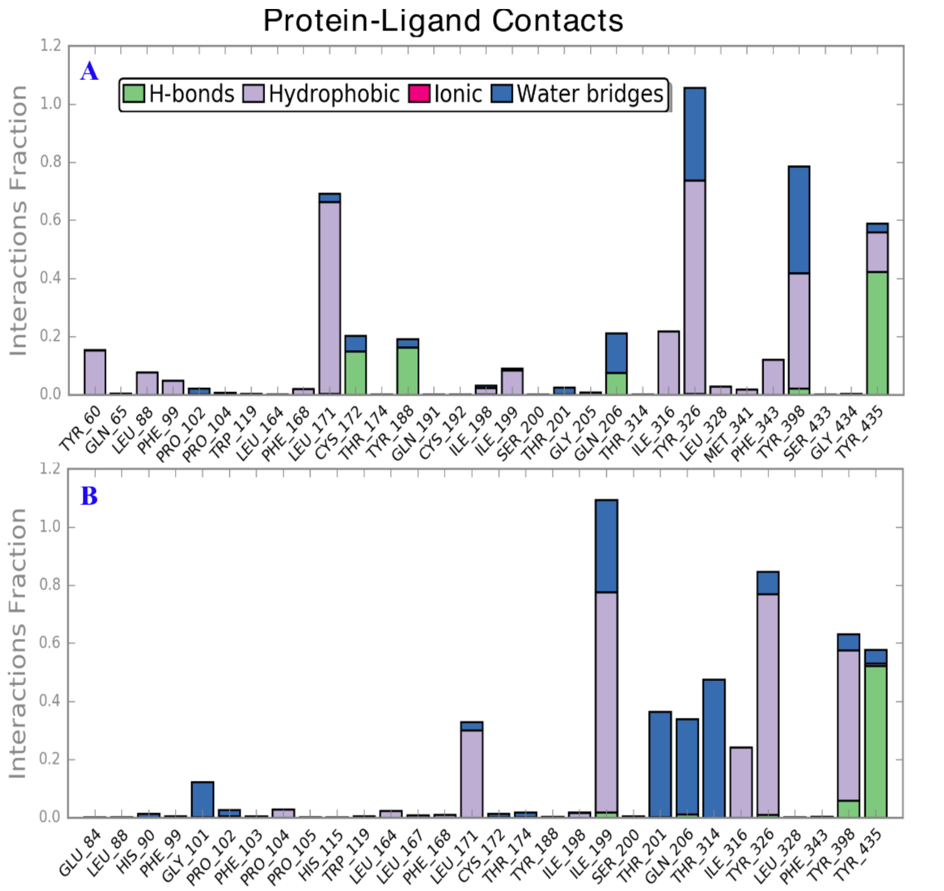

Figure S75. Protein-ligand contacts and their interaction fractions for pose 1 (A) and pose 2 (B) for acacetin 7-*O*-methyl ether.

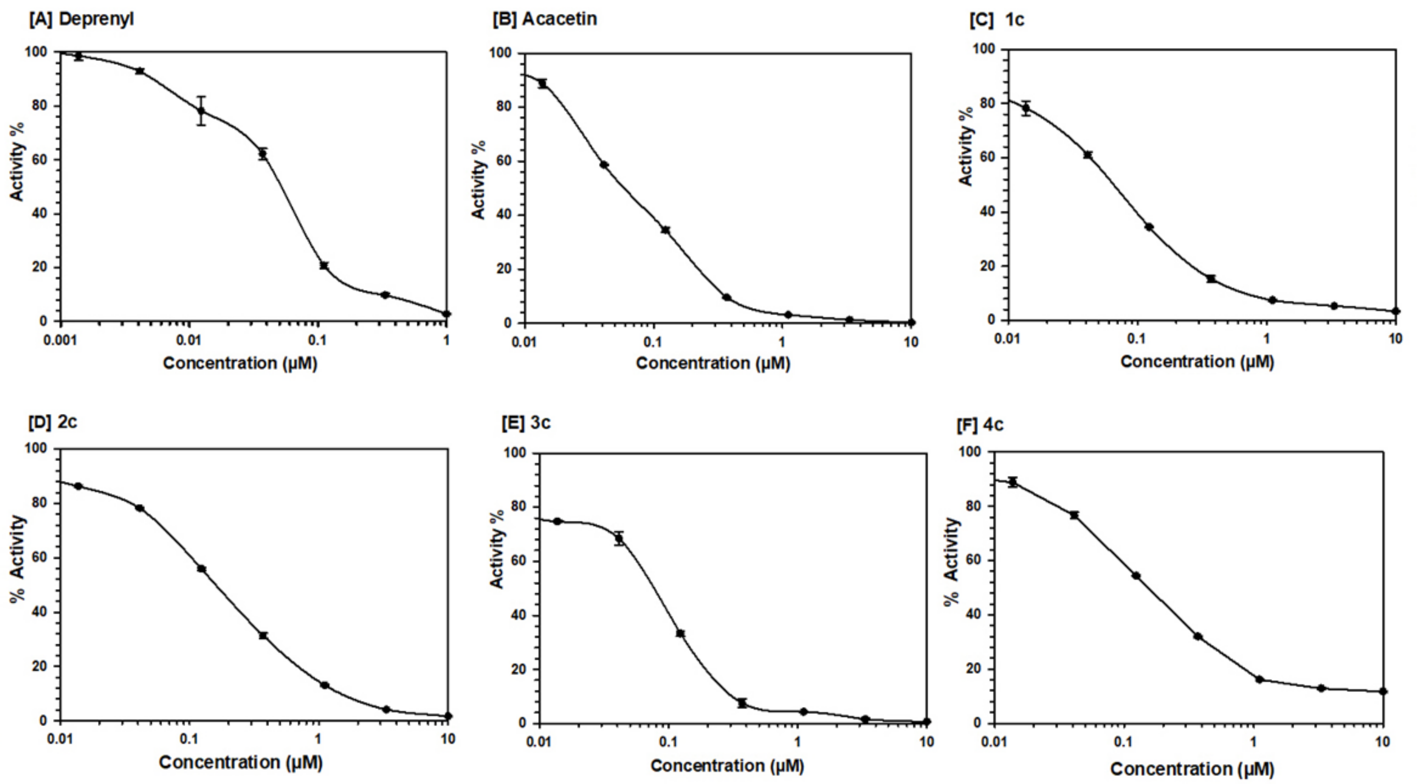

Figure S76. Dose-response profile for in vitro inhibition of recombinant human MAO-B by deprenyl, acacetin, and potent acacetin 7-*O*-methyl ether analogs (**1-4**) **c** (% activity vs concentration).

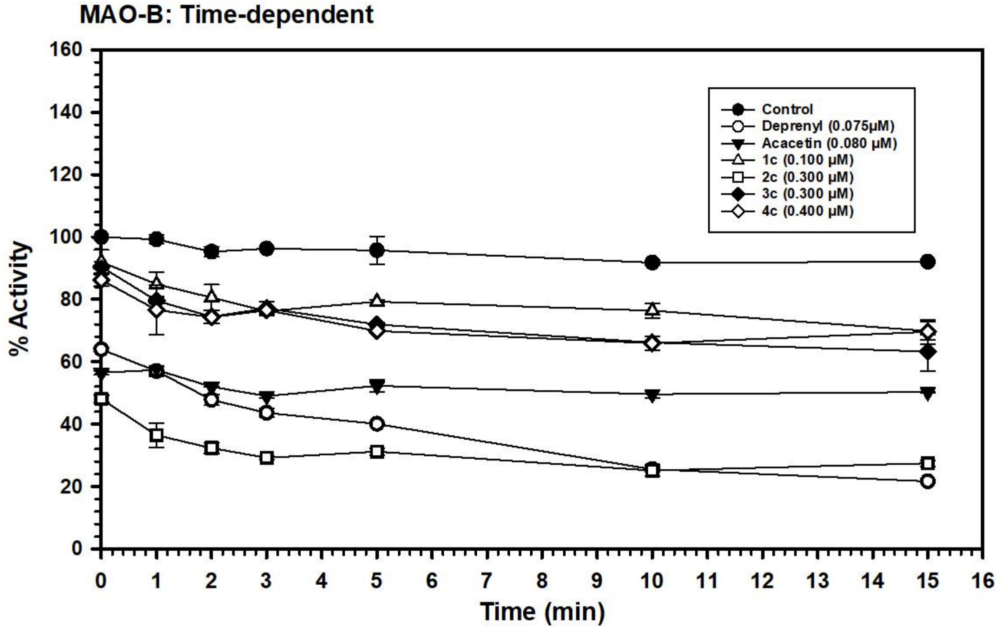

Figure S77. Time-dependent inhibition of recombinant human MAO-B by Deprenyl (0.075 μM), acacetin (0.080 μM), **1c** (0.100 μM), **2c** (0.300 μM), **3c** (0.300 μM), and **4c** (0.400 μM). Each point represents mean ± S.D. of triplicate values.

Table S1. Docking scores of acacetin 7-*O*-methyl ether analogs

Compound	MAO-A Docking score [kcal/mol]	MAO-B Docking score [kcal/mol]
**1c**	-3.511	-15.243
**2c**	-3.650	-15.102
**3c**	-2.732	-15.663
**4c**	-4.661	-15.545
**5c**	-5.434	-16.172
**6d**	-5.871	-15.451
**7d**	-3.221	-14.912
**8d**	-4.761	-16.084
**Acacetin**	-6.2	-8.1
**Acacetin 7-*O*-methyl ether**	-6.7	-8.9

Table S2. ADME Predictions for Acacetin Analogs

Compound	MW	SASA	QPlogPo/w	QPlogS	% Human oral absorption	PSA	#N and O	Rule of five	Rule of three
**1c**	326.348	618.778	4.020	-5.166	100.000	70.522	5	0	0
**2c**	326.348	611.087	3.963	-5.183	100.000	70.582	5	0	0
**3c**	340.375	640.381	4.359	-5.575	100.000	69.728	5	0	0
**4c**	322.317	592.458	3.835	-4.913	100.000	70.872	5	0	0
**5c**	338.359	637.061	4.249	-5.512	100.000	70.886	5	0	0
**6d**	325.363	621.141	3.834	-5.322	100.000	75.690	5	0	0
**7d**	325.363	612.923	3.797	-5.337	100.000	75.196	5	0	0
**8d**	337.374	639.249	4.059	-5.646	100.000	76.089	5	0	0
**Acacetin**	284.268	513.480	2.461	-3.824	87.477	85.418	5	0	0
**Acacetin 7-*O*-methyl ether**	298.295	537.958	3.141	-3.960	100.000	71.054	5	0	0
**Harmine**	212.251	444.308	3.082	-3.550	100.000	33.445	3	0	0
**Deprenyl**	187.284	454.187	2.993	-1.854	100.000	5.098	1	0	0
**Clorgyline**	272.174	547.929	4.044	-3.558	100.000	12.399	2	0	0

*MW: Molecular Weight; SASA: Solvent Accessible Surface Area; QPlogPo/w: Predicted octanol / water partition coefficient (Recommended values –2.0 to 6.5); QPlogS: Predicted aqueous solubility (log S Recommended values –6.5 to 0.5); PSA: Polar Surface Area.
